# Synthesis of Human Milk Oligosaccharides–Fluoroquinolone Conjugates and Investigation of Their Antimicrobial Activity

**DOI:** 10.1021/acs.bioconjchem.6c00334

**Published:** 2026-08-20

**Authors:** Liza Nguyen Van Sang, Emil Lindbäck, Magne O. Sydnes

**Affiliations:** † Department of Chemistry, Bioscience and Environmental Engineering, Faculty of Science and Technology, University of Stavanger, Stavanger NO-4036, Norway; ‡ Department of Chemistry, University of Bergen, Bergen NO-5020, Norway

## Abstract

The copper-catalyzed azide–alkyne cycloaddition reaction was applied to conjugate four human milk oligosaccharides (HMOs) with antimicrobial fluoroquinolones (FQs) (ciprofloxacin and sparfloxacin) to obtain a total of 12 per-*O*-acetylated and unprotected HMO–FQ conjugates. These compounds underwent antimicrobial testing against Gram-positive (*Streptococcus agalactiae*, *Enterococcus faecalis*, and *Staphylococcus aureus*) and Gram-negative (*Escherichia coli* and *Pseudomonas aeruginosa*) bacteria. The HMO–FQ conjugates exhibited the most significant antimicrobial activity against *E. coli* and *S. aureus* reaching minimum inhibitory concentration as low as 1.56 μM and 3.13 μM, respectively. A general trend was that per-*O*-acetylated HMO–FQ conjugates exhibited higher antimicrobial activity than their unprotected counterparts. The antimicrobial activity of the tested HMO–FQ conjugates is expected to arise from the ciprofloxacin and sparfloxacin moieties because per-*O*-acetylated and unprotected HMOs did not exhibit any antimicrobial activity. Additionally, the HMO–FQ conjugates displayed no cytotoxicity as evident from test on two cell lines (HepG2 and MRC5).

## Graphical abstract

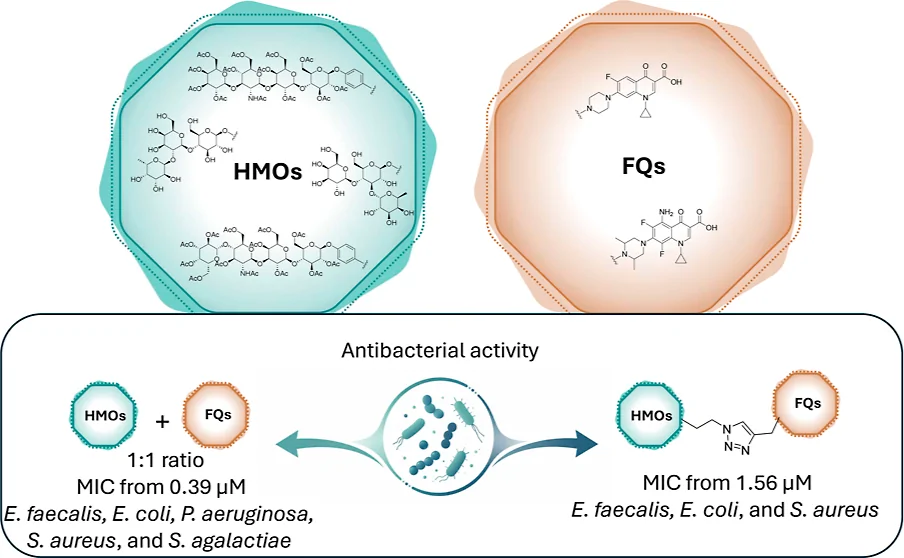

## Introduction

With the rising prevalence of antibiotic-resistant pathogens and the diminishing effectiveness of conventional antibiotics, multidrug-resistant (MDR) bacteria have emerged as a major global health threat.^1,2^ Traditional antibiotics, such as β-lactams and aminoglycosides, typically act through a single mechanism, targeting one specific site either on the cell membrane or within the bacterial cells.^3,4^ Although this single-target strategy was the main angle of approach during the golden age of antibiotic discovery and has continued to yield effective drug classes like fluoroquinolones (FQs) and carbapenems, its success is nowadays under threat.^5^ While bacteria can naturally acquire genetic mutations that confer resistance,^6^ MDR primarily emerges as a consequence of the inappropriate and excessive use of antibiotics in both clinical and agricultural settings.^7,8^ As the efficacy of commonly prescribed antibiotics continues to decline, there is an urgent need for innovative therapeutic strategies.^9^

In this context, dual-function antibacterial agents represent a promising alternative.^10–17^ By integrating multiple mechanisms of action and complementary pharmacokinetic properties into a single molecule, these agents can enhance antibacterial potency and expand the spectrum of activity.^10,18^ A key advantage of this approach is the simultaneous targeting of distinct bacterial pathways, which not only increases the likelihood of effective bacterial eradication but also significantly reduces the risk of resistance development to both mechanisms of action.^19^

Human milk oligosaccharides (HMOs) are known to promote the growth of beneficial gut bacteria while indirectly inhibiting the proliferation of harmful pathogens in infants.^20^ Beyond this prebiotic effect, HMOs can prevent the formation of bacterial biofilms, structured communities of bacteria encased in a protective matrix that contributes to antibiotic resistance.^21–25^ Additionally, it has been found that HMOs function as adjuvants by enhancing the activity of selected antifolate^15^ and ribosome targeting antibiotics.^16^

Mono- and disaccharide-functionalized conjugates of ciprofloxacin were synthesized and screened against a panel of bacteria. The results demonstrated that the activity of ciprofloxacin was retained, albeit lowered, when it was conjugated with a monosaccharide.^26^ Following this line, Meiers et al. developed a monosaccharide–ciprofloxacin antibacterial compound, which proved to possess both antibiofilm and bactericidal activity against *Pseudomonas aeruginosa*.^27^ By using a specific lectin-target probe, based on d-galactose, they observed an accumulation of the conjugate in the biofilm and a decrease of cytotoxicity compared to just ciprofloxacin alone. Further studies from the group demonstrated that *in vitro* chemical absorption, distribution, metabolism, excretion, and toxicity analysis displayed improved properties compared to ciprofloxacin, indicating that conjugates involving carbohydrates can be beneficial against bacterial infection.^28^

Building on this potential, and using the DNA-interacting properties of FQs, the objective of this study was to modify HMOs via Schmidt glycosylation, followed by copper-catalyzed azide–alkyne cycloaddition (CuAAC) to generate HMO–FQ conjugates (Figure 1).^29^ These conjugates were designed with the aim of maintaining the antimicrobial activity of FQs. Given that biofilm formation is a mechanism of antimicrobial resistance and that HMOs already have been found to exhibit such activity, we argue that our proposed compounds have the potential to contribute to the fight against MDR bacteria.

**Figure 1:**
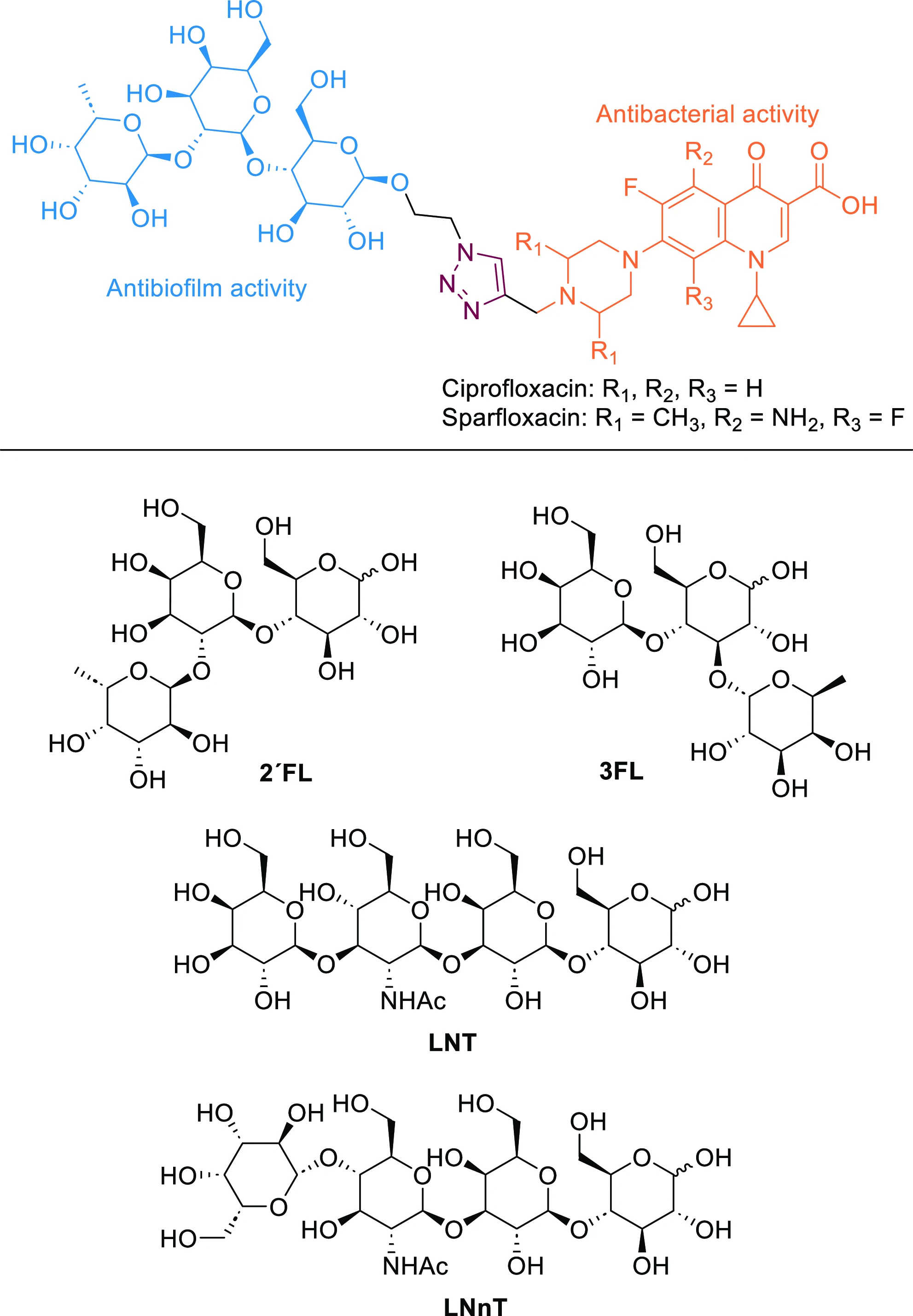
Top: model structure of target compounds. Blue: oligosaccharide (HMO), purple: triazole linker, orange: bactericidal moiety (ciprofloxacin or sparfloxacin). Bottom: structure of the oligosaccharides of interest (**2′FL**, **3FL**, **LNT**, and **LNnT**).

## Result and Discussion

### Synthesis

Four specific HMOs were chosen for this study: 2′-fucosyllactose (**2′FL**), 3-fucosyllactose (**3FL**), lacto-*N*-tetraose (**LNT**), and lacto-*N*-neotetraose (**LNnT**) (Figure 1) that exhibit promising antibiofilm activity.^22^ 2′-Fucosyllactose (**2′FL**) and 3-fucosyllactose (**3FL**) were initially selected as antibiofilm pharmacophores for the design of dual-function antimicrobial/antibiofilm, candidate compounds. Two FQs, viz. ciprofloxacin (cipro) and sparfloxacin (spar), were chosen for their well-known antimicrobial efficacy.

Since the configuration of the anomeric position can influence the biological activity of oligosaccharides,^30^ we intended to attach the antimicrobial pharmacophores to the HMOs via β-glycosidic bonds, whereas the α-anomers will be presented in a separate campaign. To enable β-selective 1,2-*trans* glycosylation via neighboring group participation, **2′FL** and **3FL** were subjected to per-*O*-acetylation using acetic anhydride, yielding the corresponding protected trisaccharides **1a** and **1b** in excellent yields (Scheme 1). The anomeric position was selected for chemical modification due to its synthetic reliability.^31–33^ Subsequent selective anomeric deacetylation of **1a** and **1b** with hydrazine acetate in tetrahydrofuran (THF) at room temperature gave the corresponding reducing sugars **2a** and **2b** in 18 and 20 h, respectively. These compounds were converted into the trichloroacetimidate donors **3a** and **3b** in high α-selectivity upon reaction with trichloroacetonitrile in the presence of cesium carbonate giving the desired products in 62% and 69% yield, respectively (Scheme 1).^34^ The glycosyl donor, 2-azidoethanol (**5b**), was obtained upon treatment of 2-bromoethanol (**5a**) with sodium azide.^35^

**Scheme 1:**
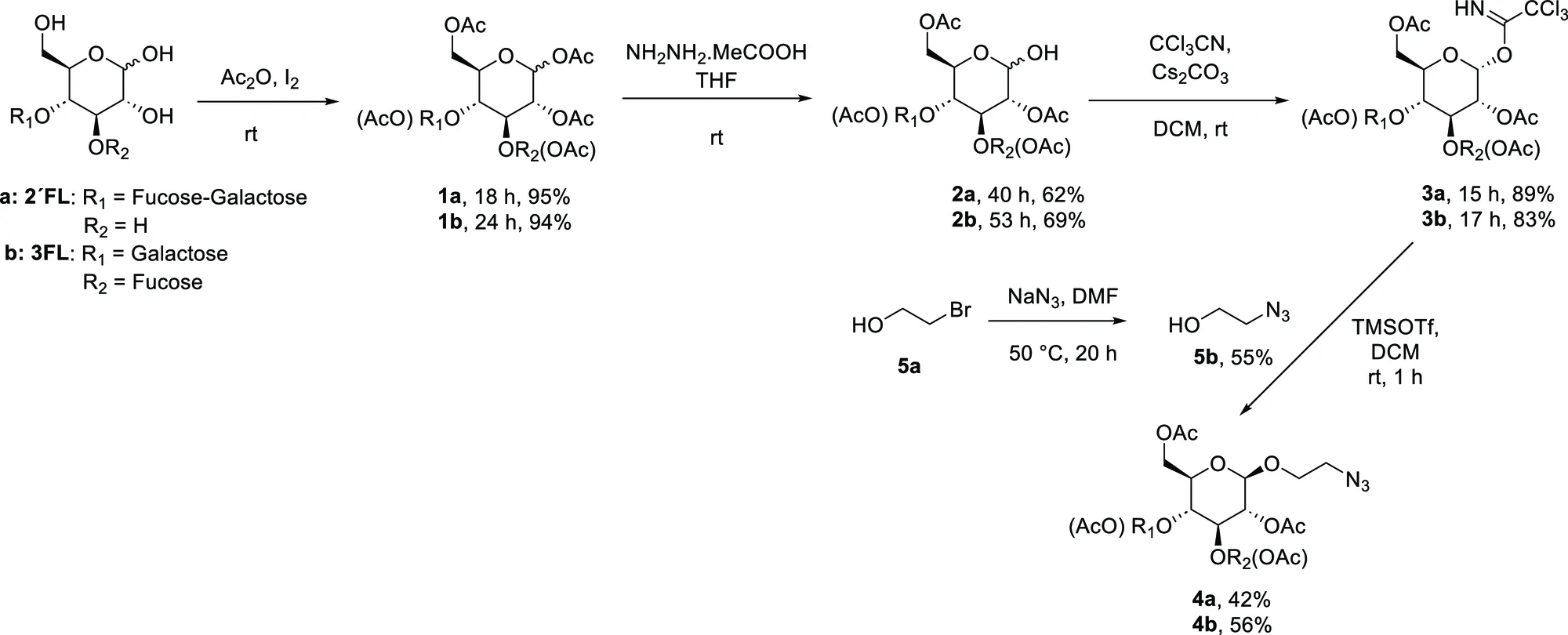
Synthesis of Azido Compounds from **2́FL** and **3FL**

Glycosylation of **3a** and **3b** with **5b** was originally intended to be carried out using boron trifluoride diethyl etherate (BF_3_·OEt_2_) since this reagent is known to yield selective *O*-glycosylation.^36^ However, the reaction utilizing BF_3_·OEt_2_ failed, resulting in a complex mixture of products and likely damaging the oligosaccharide as evident from crude ^1^H NMR analysis.^37,38^ Switching to trimethylsilyl trifluoromethanesulfonate (TMSOTf) as a promoter enabled successful Schmidt glycosylation, affording products **4a** and **4b** in good yields after 1 h at room temperature under an inert atmosphere. The exclusive formation of the β glycosidic bond in both **4a** and **4b** was confirmed by ^1^H NMR analysis where the signal at 4.53 ppm (*J* ≈ 8.1 Hz) was attributed to the β-anomer proton (Scheme 1). Supporting this assignment, the ^13^C NMR and HSQC ^1^H–^13^C spectra showed the characteristic signal of an anomeric carbon at 100.5 ppm, consistent with the formation of β-glycosidic linkages.^39^

To prepare the alkyne-functionalized fragments, the corresponding FQs were *N*-propargylated using Damera’s procedure,^40^ involving nucleophilic substitution with propargyl bromide in the presence of K_2_CO_3_ in DMF (Scheme 2). The overall yield for compound **6** did not exceed 25%, while compound **7** was obtained in yields ranging from 45% to 50%. This difference in yield may be attributed to the electron-donating methyl group on the piperazine ring of sparfloxacin, which increases the nucleophilicity of the nitrogen. In addition, the poor solubility of ciprofloxacin in acetonitrile may also have influenced the outcome of the reaction.

**Scheme 2:**
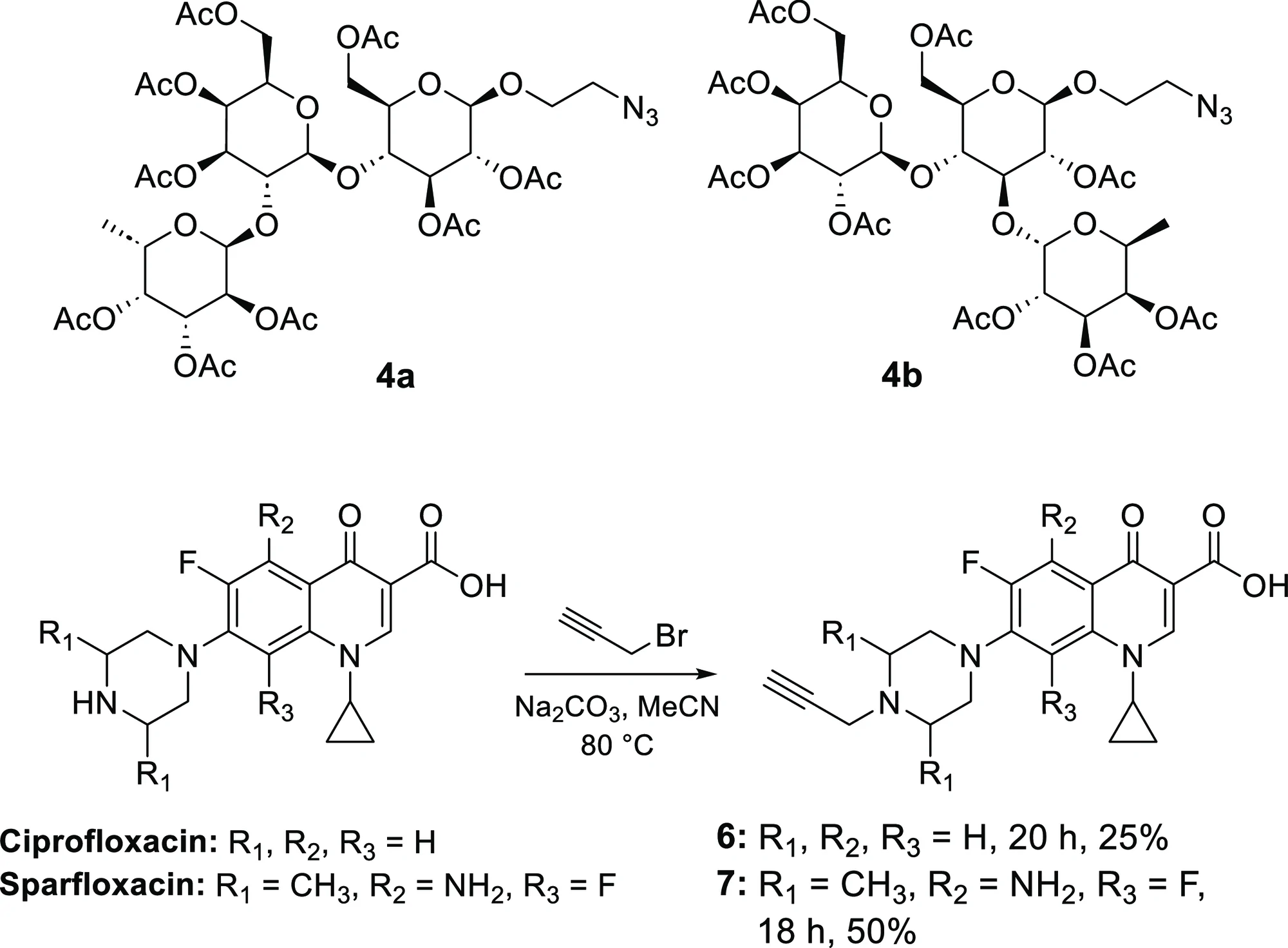
Synthesized Fragments for Constructing Dual Compounds

With the antibiofilm elements **4a** and **4b** and the antimicrobial pharmacophores **6** and **7** (Scheme 2) in hand, we were set up for the [3 + 2] cycloaddition to afford the desired conjugates.^41^

To this end, catalytic amounts of copper­(II) sulfate pentahydrate (CuSO_4_·5H_2_O) and sodium ascorbate were added to a mixture of azide **4a** and alkyne **6** in DMF, and the reaction mixture was stirred at room temperature. The CuAAC is widely reported and generally proceeds smoothly.^42–44^ However, due to the bidentate coordination ability of the FQ diketone moiety with copper ions (Figure 2),^45^ it appeared necessary to increase the catalyst loading.

**Figure 2:**
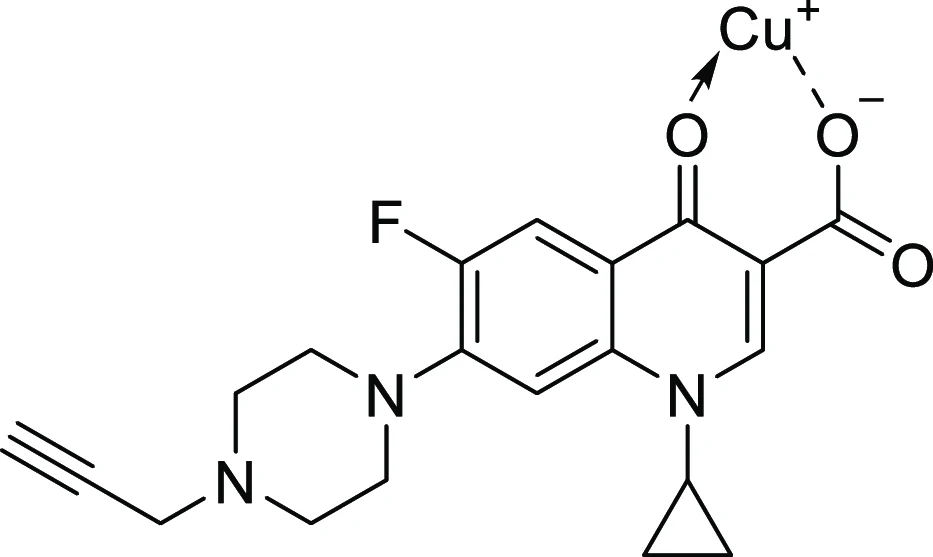
Plausible structure of the copper complex with ciprofloxacin analogue **6**.

Specifically, 1.2 equiv of CuSO_4_ and 1.6 equiv of sodium ascorbate were required for efficient conversion, compared to the standard 0.2 equiv of both reagents. Under these optimized conditions, the corresponding triazole-linked conjugates **8a**–**8d** were successfully synthesized. Copper residues in the product mixture were removed by washing the reaction mixture with 10% w/w Na_2_S_2_O_3_ (see the Experimental Section for details), which reduces Cu^2+^ to Cu^+^ followed by formation of a copper­(I) thiosulfate complex that is soluble in an aqueous solution.^46^ Subsequent deprotection of the acetate groups using sodium methoxide (MeONa)^47^ afforded the final compounds **9a**–**9d** in yields ranging from 47% to 65% (Scheme 3).

**Scheme 3:**
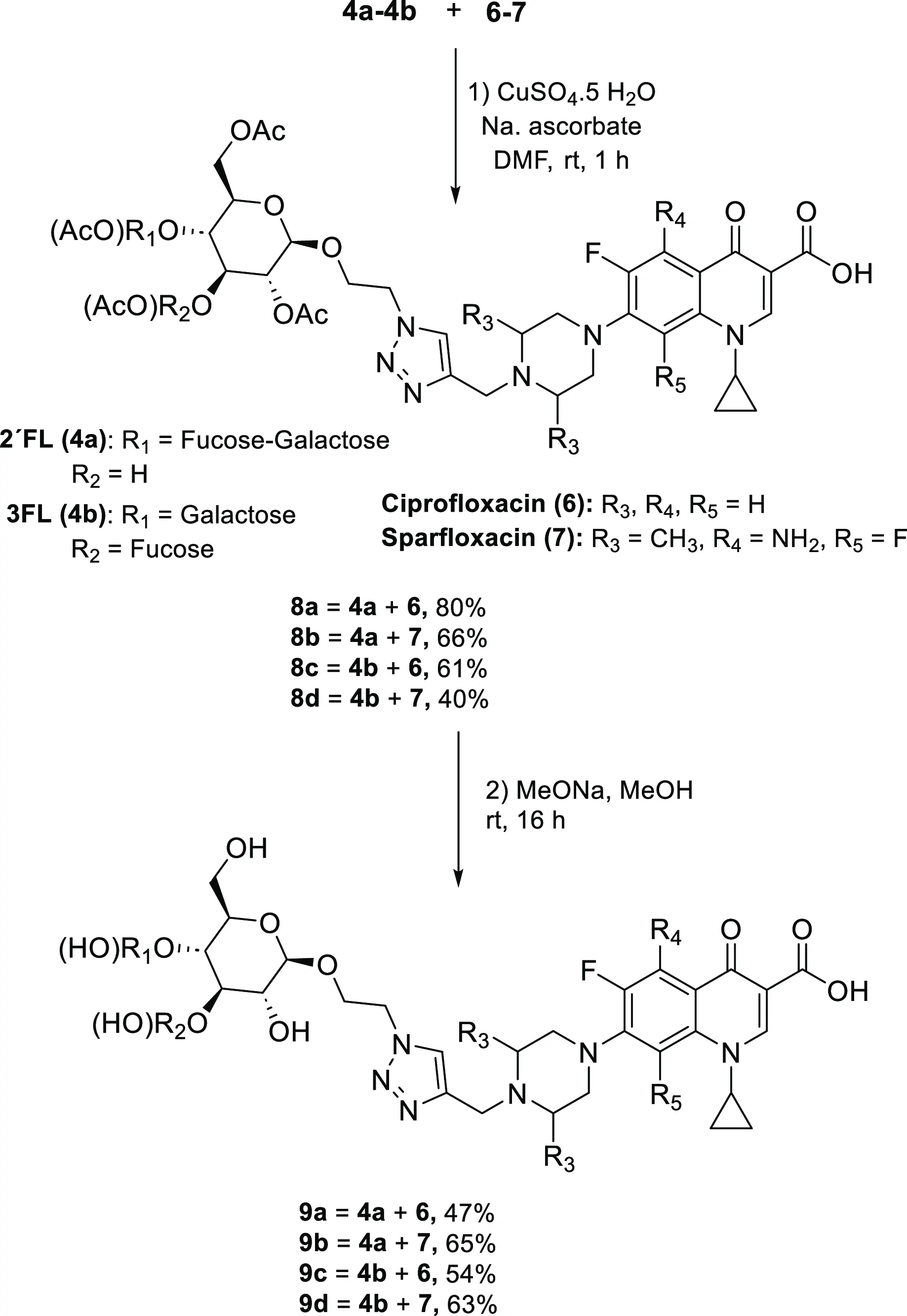
Two-Step Synthesis of **8a**–**8d** and **9a**–**9d**

Attempts to attach the azide linker to **LNT** and **LNnT** using the strategy outlined in Scheme 1 proved to be less effective compared to **2′FL** and **3FL**. During the trichloroacetimidate step, no α-selectivity was observed, resulting in a mixture of α- and β-anomers, possibly caused by the difference in flexibility between the tri and tetra saccharides.^48^ Fucosylated HMOs **2́FL** and **3FL** are structurally more constrained^49^ compared to **LNnT** or **LNT**, which have been proven to exist in two conformations in solution with a rotational bond Ψ at the GlcNAc-β-1 → 3-Gal-β linkage (Figure 3).^50^

**Figure 3:**
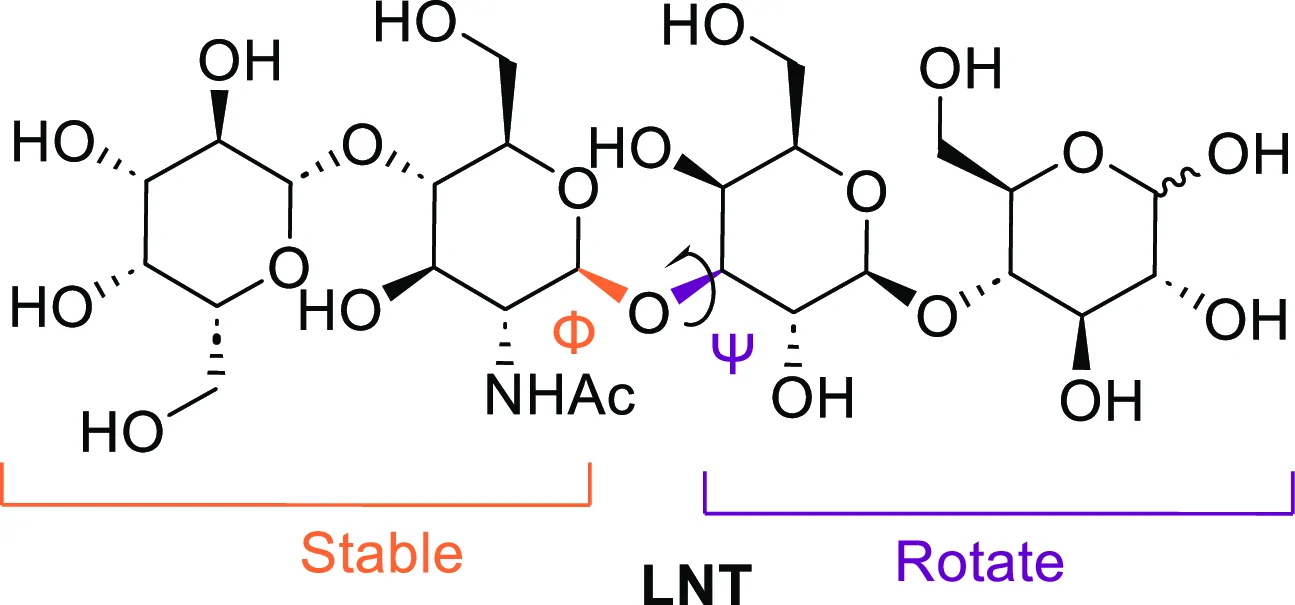
Structure of **LNT** highlighting the rotational bond Ψ.

Subsequent glycosylation attempts with this mixture were unsuccessful, causing a revision of the synthetic approach. Starting from the *O*-acetylated oligosaccharides, a new strategy was employed in which the hydroxyl group of compound **5b** was attempted to be directly coupled to the oligosaccharide using a BF_3_·OEt_2_-mediated glycosylation procedure (Scheme 4).^51^

**Scheme 4:**
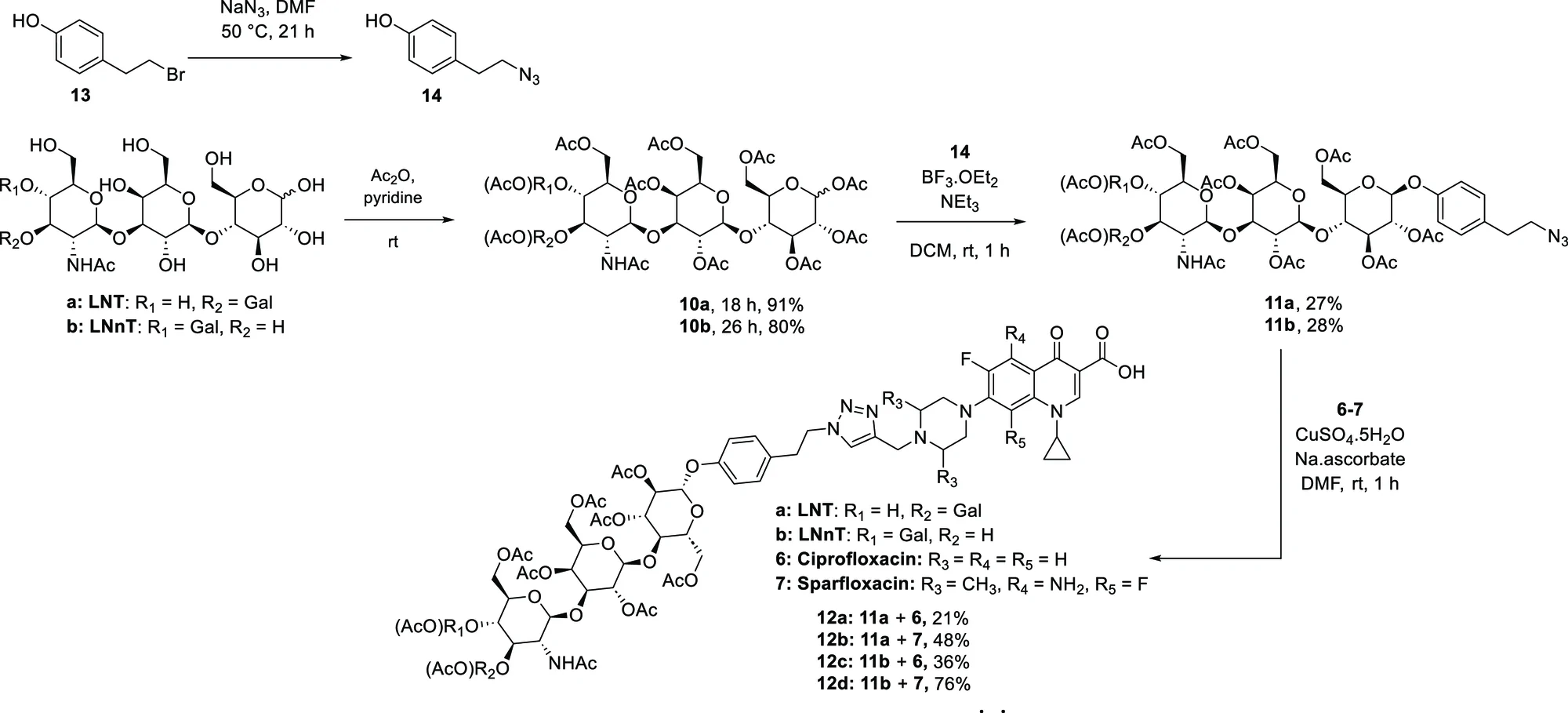
Synthetic Pathway for Compounds **12a**–**12d**

Although **LNT** and **LNnT** do not contain a fucose unit and showed no signs of degradation, the glycosylation attempt between OAc-protected **LNT** and compound **5b** once again failed. Previous studies by Li et al.^52^ demonstrated that BF_3_·OEt_2_ is the preferred catalyst for phenol *O*-glycosylation, while TMSOTf is generally more effective for reactions involving aliphatic alcohols. Based on this, a phenol was selected to be coupled via the BF_3_·OEt_2_-mediated approach. While the reported method involved a glycosyl trichloroacetimidate, 4-(2-azidoethyl)­phenol (**14**), synthesized from 4-(2-bromoethyl)­phenol (**13**), was reacted with **10a**. This yielded compound **11a** in 27% yield with exclusive β-selectivity (Scheme 4). Applying the same protocol to **10b** afforded **11b** in 28% yield. Analysis of both ^1^H and ^13^C NMR data confirmed the β-configuration at the terminal anomeric position.

The final CuAAC reactions between **11a**–**11b** and compounds **6** and **7**, using adjusted equivalents of copper and sodium ascorbate, successfully yielded the desired hybrids **12a**–**12d** in a broad range of yields (21%–76%) (Scheme 4). Deacetylation was attempted under similar conditions as previously used to form compounds **9a**–**9d**; however, the additional sugar unit renders the compounds extremely polar, requiring more than 50% water in the eluent for the silica gel column chromatography. Despite this, a very short column, expected to result in slow elution, was tested using MeCN/H_2_O (6:4) as a solvent system; however, this solvent system was not polar enough to eluate the product, and a more polar system was not tested since that would wash out the silica gel and contaminate the product. Given that the acetylated products, viz. **8a**–**8c**, showed significantly better activity compared to the free hydroxyl analogues (**9a**–**9c**) (*vide infra*), further purification efforts were abandoned at this stage.

### Biological Evaluation

A total of 12 target HMO–FQ conjugates, viz. **8a**–**8d**, **9a**–**9d**, and **12a**–**12d**, were subjected to screening against Gram-positive (*Streptococcus agalactiae*, *Enterococcus faecalis*, and *Staphylococcus aureus*) and Gram-negative (*Escherichia coli* and *P. aeruginosa*) bacteria. Through an established protocol,^53^ bacteria were grown on a corresponding medium incubated at 37 °C overnight (see the Supporting Information for details), and compounds **8a**–**8d**, **9a**–**9d**, and **12a**–**12d** were solubilized in a serial dilution with final concentrations of 100 μM, 50 μM, 25 μM, 12.5 μM, 6.25 μM, 3.13 μM, and 1.56 μM and were tested in order of decreased concentration on the bacterial strains listed above. The minimum inhibition concentrations (MIC, μM) are summarized in Table 1.

**Table 1:** Summary of the MIC (μM) Results of Compounds **8a**–**8d**, **9a**–**9d**, and **12a**–**12d** against 3 Strains of Bacteria (*E. faecalis*, *E. coli*, and *S. aureus*)

	MIC (μM)			toxicity (μM)	
#	E. faecalis	E. coli	S. aureus	HepG2	MRC5
**8a**	25	**1**.**56**	**3**.**13**	I	I
**8b**	25	**1**.**56**	**3**.**13**	I	I
**8c**	50	3.13	6.3	I	I
**8d**	I	50	100	I	I
**9a**	I	75	I	I	I
**9b**	I	6.3	50	I	I
**9c**	I	6.3	50	I	I
**9d**	I	25	I	I	I
**12a**	I	6.3	25	I	I
**12b**	I	12.5	I	I	I
**12c**	I	100	I	I	I
**12d**	50	6.3	12.5	I	I
**Cipro** ^a^	1.51 ^58^	0.024 ^59^	1.51 ^60^	/	/
**Spar** ^a^	0.64 ^61^	0.64 ^62^	0.64 ^62^	/	/

a The MIC of ciprofloxacin (cipro) and sparfloxacin (spar) were extracted from the literature in μg/mL and converted to μM for comparison.^59–63^ I = Inactive at 100 μM.

The best activity was found for **8a** and **8b** against *E. coli* with a MIC of 1.56 μM, while the least active compound was **12c** that inhibited the growth of *E. coli* at 100 μM. The **2′FL**-based compounds are generally more active than **3FL** (**8a** and **8b** compared to **9c** and **9d**) against all the bacterial strains displayed in Table 1. In contrast, no such trend can be applied to the **LNT** and **LNnT** series with **12a** and **12d**. Both exhibited a MIC value of 6.3 μM against *E. coli*, and **12c** only has a MIC of 100 μM (Table 1).

It is also interesting to note that the protected versions (AcO−), viz. compounds **8a**–**8d**, based on **2́FL** or **3FL**, are more active against the tested strains of bacteria than their unprotected (HO−) homologue (**9a**–**9d**), with the only exception being compound **8d** that did not follow the trend. Direct acetylation is a method used for prodrug formulation.^54^ Acetyl groups are reported to mask polar functionalities, typically hydroxyl, and improving pharmacokinetic properties, including absorption, solubility, or stability. Because membrane permeation is regulated by a balance between hydrophobicity and lipophobicity,^55^ the internalization of the compounds might be facilitated by the *O*-acetylation. If Gram-negative bacteria are notably resistant to hydrophobic antibiotics,^56^ the number of free hydroxyl groups in **9a**–**9d** is quite high, and the uptake of **8a**–**8d** is possibly improved compared to the unprotected version. However, over-acetylation of antibacterial compounds is not necessarily the ultimate solution. In a study from Mellegård, chitosan with a higher degree of acetylation has been found to be less active than those with fewer acetyl groups.^57^

Three strains of bacteria showed sensitivity, but two of them (one Gram-positive and one Gram-negative, viz. *S. agalactiae* and *P. aeruginosa*, respectively) were unaffected. Even though ciprofloxacin and sparfloxacin have strong activity toward *P. aeruginosa*,^64,65^ it seems that the oligosaccharide unit significantly reduces their potency, and none of the compounds prepared prevented the growth of this bacterium. On the other hand, the activity of FQs against *S. agalactiae* is less unanimous with a MIC reported to vary from 0.06 μg/mL, up to 16 μg/mL for ciprofloxacin;^65,66^ hence, it is not so surprising that no inhibition has been demonstrated for this strain.

To evaluate the contribution of each moiety and assess the potential benefit of linking the two fragments, the individual fragments and mixtures were tested against the same bacterial strains (*E. faecalis*, *E. coli*, *P. aeruginosa*, *S. aureus*, and *S. agalactiae*) (Table 2). Protected and unprotected HMOs were tested independently, and no activity was detected. The presence of the azido linker did not improve the potency, and **4a**–**4b** were found to be inactive. On the contrary, products **6** and **7** showed inhibition of almost every bacterial strain with activity approaching those found for ciprofloxacin and sparfloxacin. Against *S. aureus*, compounds **6** and **7** showed activity at the same order of magnitude as ciprofloxacin and sparfloxacin (compound **6**, 1.5 μM vs ciprofloxacin, 1.51 μM and compound **7**, 1.5 μM vs sparfloxacin, 0.64 μM) (Table 1 and 2). Most likely, this confirms that the activity observed by the conjugates is due to the FQs mode of action and that the HMOs do not possess bactericidal properties for the strains tested in this study.

**Table 2:** Summary of the MIC (μM) Results of Compounds **8a**–**8d**, **9a**–**9d**, and **12a**–**12d** against Five Strains of Bacteria (*E. faecalis*, *E. coli*, *P. aeruginosa*, *S. aureus*, and S. agalactiae)^a^

	MIC (μM)				
#	*E. faecalis*	*E. coli*	*P. aeruginosa*	*S. aureus*	*S. agalactiae*
**2′FL**	I	I	I	I	I
**3FL**	I	I	I	I	I
**1a**	I	I	I	I	I
**1b**	I	I	I	I	I
**10a**	I	I	I	I	I
**10b**	I	I	I	I	I
**4a**	I	I	I	I	I
**4b**	I	I	I	I	I
**6**	12.5	3.1	I	1.5	50
**7**	6.25	1.5	25	1.5	25
**2′FL** + **Cipro**	1.56	I	18.75	18.75	I
**2′FL** + **Sparflo**	25	0.79	I	6.25	I
**3FL** + **Cipro**	0.78	18.75	I	18.75	I
**3FL** + **Sparflo**	18.75	1.56	I	6.25	I
**1a** + **Cipro**	**3**.**13**	**0**.**39**	**0**.**78**	**0**.**78**	**6**.**25**
**1a** + **Sparflo**	**6**.**25**	**0**.**39**	**6**.**25**	**0**.**78**	**12**.**5**
**1b** + **Cipro**	**6**.**25**	**0**.**39**	**1**.**56**	**1**.**56**	**12**.**5**
**1b** + **Sparflo**	6.25	0.39	12.5	1.56	6.25
**10a** + **Cipro**	**6**.**25**	**0**.**39**	**1**.**56**	**1**.**56**	**12**.**5**
**10a** + **Sparflo**	**0**.**78**	**0**.**39**	**1**.**56**	**0**.**39**	**3**.**13**
**10b** + **Cipro**	12.5	1.56	3.13	3.13	25
**10b** + **Sparflo**	3.13	1.56	6.25	1.56	12.5
**4a** + **6**	12.5	1.56	I	1.56	100
**4a** + **7**	3.13	1.56	25	1.56	25
**4b** + **6**	25	3.13	I	3.13	I
**4b** + **7**	6.25	1.56	25	1.56	25
**Cipro**	1.51 ^58^	0.024 ^59^	0.75 ^67^	1.51 ^60^	1.89 ^68^
**Sparflo**	0.64 ^61^	0.64 ^62^	2.55 ^69^	0.64 ^62^	1.27 ^70^

a I = Inactive at 100 μM.

Different equimolar mixtures of the fragments were also tested under the same conditions as the parent conjugates. First, unprotected oligosaccharide **2′FL** or **3FL** and their protected analogues **1a** or **1b** were combined in a ratio of 1:1 with ciprofloxacin and sparfloxacin. In correlation with previous observation, this revealed that the presence of the OAc group enhances the overall antibacterial activity. The mixture “**2′FLL**+**Cipro**” did not inhibit *E. coli* or *S. agalactiae*, while its OAc-homologue “**1a**+**Cipro**” eliminated both strains with a MIC of 0.39 μM and 6.25 μM, respectively (Table 2). The activity against *P. aeruginosa* and *S. aureus* was also improved with a MIC below 1 μM for “**1a** + **Cipro**” compared to 18.75 μM for “**2′FL** + **Cipro**”. Similar observations are made between “**3FL** + **Sparflo**” and “**1b** + **Sparflo**” with general activity being improved when the oligosaccharide is OAc protected (Table 2).

Second, the biological data for the combination of azido-HMOs (**4a**–**4b**) and alkylated-FQs (**6**–**7**) demonstrated that mixtures of fragments with azido/alkyne linkers are less active than those without linkers. For example, “**4b** + **6**” inhibited *E. faecalis* with a MIC of 25 μM and was inactive against *P. aeruginosa* and *S. agalactiae*, while the activity of “**1b**+**Cipro**” against *E. faecalis* was improved to a MIC of 6.25 μM and attained a MIC of 1.56 μM and 12.5 μM against *P. aeruginosa* and *S. agalactiae*, respectively.

Conjugates were compared to the mixture of their respective moieties. In the case of **2′FL** and **3FL**, the conjugates were less active than the mixtures with the most important MIC difference between **8d** and “**1b** + **Sparflo**”. Conjugate **8d** exhibited very low activity against *E. coli* and *S. aureus*, whereas the 1:1 ratio mixture of the two moieties, “**1b** + **Sparflo**”, was able to eradicate not only those two strains but also *E. faecalis*, *P. aeruginosa*, and *S. agalactiae*. Even in the case of the conjugates displaying the overall best activity, namely **8a**–**8b**, the corresponding mixtures were considerably more potent. Both **8a** and **8b** inhibited *E. coli* with a MIC of 1.56 μM and *S. aureus* at 3.13 μM, which was an improvement of up to 4-fold compared to the mixtures “**1a** + **Cipro”** and **“1a** + **Sparflo”** inhibiting the two strains with a MIC of 0.39 μM and 0.78 μM, respectively (Table 2). The difference in bactericidal activity was all the more significant with **LNT** and **LNnT**. The conjugates based on those two HMOs displayed modest activity, with the best MIC at 6.3 μM against *E. coli* (Table 1). Most of them were inefficient against *E. faecalis* and *S. aureus* with only minor activity found for **12d** (MIC of 50 μM and 12.5 μM, respectively). In comparison, the mixtures of **10a**–**10b** and **Cipro**/**Sparflo** were able to suppress all five strains with MICs ranging from 12.5 μM down to 0.39 μM.

The contrast of activity between **9a**–**9d** and their respective mixtures suggests that the linkage is not optimal for antibacterial activity. While **9b** is only weakly active against *E. coli* and *S. aureus* (MIC of 6.3 μM and 50 μM, respectively), the combination of “**2′FL** + **Sparflo**” displayed an improved MIC of 0.79 μM and 6.25 μM, respectively. Although the linkage between FQs and HMOs does not enhance the antibacterial activity, the mixtures in a 1:1 ratio are more interesting and performed better than their FQs parents against some bacterial strains. For example, ciprofloxacin inhibits *E. faecalis* at MIC 1.51 μM, while the mixture “**3FL** + **Cipro**” is efficient at 0.78 μM, suggesting that the HMOs have a beneficial impact on the overall activity, despite not being bactericidal. Against *E. coli*, ciprofloxacin is better than the mixture, but “**1a** + **Sparflo**”, “**1b** + **Sparflo**”, and “**10a** + **Sparflo**” all exhibit improved activity compared to sparfloxacin. Similar observations are made for *S. aureus* with ciprofloxacin inhibiting the strain at 1.51 μM and sparfloxacin at 0.64 μM, while the mixtures “**1a** + **Cipro**”, “**1a** + **Sparflo**”, and “**10a** + **Sparflo**” prevent the growth of the bacteria at 0.78 μM and 0.39 μM, respectively.

Lastly, the toxicity of the conjugates, individual moiety, and mixtures was tested on two cell lines, HepG2 and MRC5, tumorigenic liver cells, and normal human lung cells, respectively. None of the compounds or mixtures displayed toxicity against either cell line (Table 1), suggesting their potential safety for use and opening possibilities for their application.

## Conclusion

New antibacterial HMO-based compounds were successfully synthesized linking four oligosaccharides (**2′FL**, **3FL**, **LNT**, and **LNnT**) to two FQs (ciprofloxacin and sparfloxacin), via a triazole linker, employing a CuAAC reaction. The target molecules **8a**–**8d**, **9a**–**9d**, and **12a**–**12d** exhibiting activity with MIC values ranging from 1.56 μM for the most potent compounds, viz. **8a** and **8b**, up to 100 μM for those with a weaker activity against *E. coli* and *S. aureus*. Only compounds **8a**–**8c** and **12d** showed some activity toward *E. faecalis*, while *S. agalactiae* and *P. aeruginosa* were unaffected by all compounds (**8a**–**8d**, **9a**–**9d**, and **12a**–**12d**). *E. coli* showed highest sensitivity with all compounds demonstrating activity against that bacterium. None of the compounds tested manifested toxicity against HepG2 or MRC5 cells.

Further biological evaluation of the HMOs and FQs separately demonstrated that HMOs alone do not possess antibacterial activity against the selected strains. In contrast, the alkylated ciprofloxacin **6** and sparfloxacin **7** were efficient at suppressing most strains at low concentrations, indicating that the observed activity from the conjugates might be attributed to FQs. The presence of OAc protecting groups on the oligosaccharide seems to enhance the global antibacterial activity and is true for both linked and unlinked compounds. Biological data also suggested that the average MIC value tends to increase with the addition of a linker (azido or alkyne) and further with the linkage between two fragments, demonstrating that the use of individual fragments in a 1:1 ratio is more effective against bacteria than their linked counterparts.

More importantly, the observed biological data demonstrated that a 1:1 mixture of FQs and HMOs improved the antibacterial activity compared to individual FQs. Although the mode of action is probably attributable to the FQs, the presence of HMOs in the mixture enhances the antibacterial activity. Considering that HMOs do not possess bactericidal effect, the improvement may be the result of antibiofilm activity. This hypothesis is yet to be confirmed with further studies ongoing to elucidate the compound́’s ability to prevent biofilm formation. These results will be reported in due course.

## Experimental Section

### General

All chemicals were purchased from Sigma-Aldrich/Merck or VWR and used without any purification. Solvents were dried over 4 Å molecular sieves. Thin layer chromatography (TLC) visualization was achieved with UV light (254 and/or 365 nm) and a H_2_SO_4_/EtOH (1:9) dip for oligosaccharides. All purifications were performed by silica-gel column chromatography with a hand pump and 230–400 mesh silica gel. IR spectra were recorded on an Agilent Cary 630 FT-IR spectrophotometer. UV–vis spectra were obtained on an Agilent 8453 single-beam. Melting points (mp) were determined on a Stuart SMP20 melting point apparatus and are uncorrected. NMR spectra were recorded on a Bruker Ascend 400 spectrometer (^1^H: 400.13 MHz, ^13^C: 100.61 MHz, and ^19^F: 376.46 MHz). Coupling constants (*J*) are given in Hz, and the multiplicity is reported as singlet (s), doublet (d), triplet (t), quartet (q), sextet (s), multiplet (m), and broad singlet (bs). Chemical shifts are reported in ppm, and calibration is performed using the residual solvent signals: CDCl_3_ (^1^H: 7.26 ppm and ^13^C: 77.16 ppm), MeOD (^1^H: 3.31 ppm and ^13^C: 49.00 ppm), or D_2_O (^1^H: 4.79 ppm). High-resolution mass spectra were obtained on a JEOL AccuTOF T100GC mass spectrometer. The instrument was operated with an orthogonal ESI source, an orthogonal accelerated time-of-flight (TOF) single-stage reflectron mass analyzer, and a dual microchannel plate. Mass calibration was performed using the internal standard method, and mass drift compensation was performed in each acquisition. No unexpected or unusually high safety hazards were encountered for any of the chemicals used in this study.

#### General Procedure for Peracetylation (2′FL, 3FL, LNT, and LNnT)

Oligosaccharide (1 equiv) and iodine (0.05 equiv) or pyridine (0.05 equiv) were dissolved in acetic anhydride, and the resulting reaction mixture was stirred at rt for 18 h. The solvent was removed under *vacuo*, and the crude mixture was purified by silica gel column chromatography using pet. ether/EtOAc as a solvent. Isolation of the relevant fraction yielded the title compound.

#### General Procedure for Selective Anomeric Position (2′FL and 3FL)

Peracetated HMO (1 equiv) and hydrazine acetate (2 equiv) were dissolved in dry THF, and the resulting reaction mixture was stirred at rt between 18 and 20 h. The reaction was quenched with water (15 mL) before being extracted with EtOAc (1 × 20 mL). The organic layer was washed with NaHCO_3_ (2 × 20 mL) and dried over MgSO_4_. Filtration and concentration gave the crude product that was purified by silica gel column chromatography using pet. ether/EtOAc as a solvent. Isolation of the relevant fractions yielded the title compound.

#### General Procedure for α-Selective Trichloroacetimidation (2′FL and 3FL)

Corresponding oligosaccharide (1 equiv), trichloro acetonitrile (2 equiv), and cesium carbonate (0.1 equiv) dissolved in DCM were stirred at rt for up to 17 h. The solvent mixture was evaporated, and the crude residue was purified by silica-gel column chromatography using pet. ether/EtOAc as a solvent. Isolation of the relevant fractions yielded the α-selective trichloroacetimidation compound.

#### General Procedure for Glycosylation with TMSOTf (2′FL and 3FL)

To a solution of the appropriate oligosaccharide (1 equiv) and 1-azidoethanol (2 equiv) in dry DCM were added 10 drops of TMSOTf, under an inert atmosphere. The reaction mixture was stirred at rt for 1 h. When the TLC analysis showed that the starting material was consumed, a few drops of NEt_3_ were added, and the mixture was stirred for an additional 10 min, before being filtered through Celite and concentrated. The residue was purified by silica-gel column chromatography using pet. ether/EtOAc as a solvent. Isolation of the relevant fractions yielded the β-selective azido product.

#### General Procedure for Glycosylation with BF_3_·OEt_2_ (LNT and LNnT)

Acetated HMO (1 equiv), 4-(2-azidoethyl) phenol (1.5 equiv), and NEt_3_ were dissolved in dry DCM under an inert atmosphere, prior to addition of BF_3_·OEt_2_ (7 equiv). The reaction mixture was stirred at rt for 1 h, before being quenched with NaHCO_3_ aq. sat. (10 mL), extracted with DCM (3 × 15 mL), and dried over MgSO_4_. Filtration and concentration gave a crude product that was purified by silica-gel column chromatography using pet. ether/EtOAc as a solvent. Isolation of the relevant fractions yielded the β-selective azido product.

#### General Procedure for *N*-Propargylation

A solution of the corresponding FQ (1 equiv) and K_2_CO_3_ (2 equiv) was stirred in DMF (0.5 mL) at rt. After 5 min, propargyl bromide (5 equiv) was added, and the reaction mixture was stirred at reflux for 17 h. The crude product was extracted with EtOAc (3 × 2 mL), and the combined organic fractions were washed with brine (1 × 5 mL), and the solvent was evaporated. The residue was purified by silica gel column chromatography using pet. ether/EtOAc as a solvent. Isolation of the relevant fractions yielded the title compound.

#### General Procedure for CuAAC [3 + 2] Cycloaddition (2′FL, 3FL, LNT, and LNnT)

Copper­(II) sulfate pentahydrate (1.2 equiv) and sodium ascorbate (1.6 equiv) were added to a stirred solution of azide (1 equiv) and alkyne (1 equiv) in dry DMF (2 mL) under an inert atmosphere. The resulting reaction mixture was stirred at rt for 2 h, followed by dilution with EtOAc (5 mL). The organic phase was washed with 10% w/w Na_2_S_2_O_3_ aqueous solution (3 × 5 mL) and dried over MgSO_4_. Filtration and concentration under reduced pressure gave the crude product that was subjected to purification by silica gel column chromatography using pet. ether/EtOAc as a solvent. Isolation of the relevant fractions gave the title compound.

#### General Procedure for OAc Deprotection (2′FL and 3FL)

Oligosaccharide (1 equiv) and MeONa (3 equiv) were dissolved in dry MeOH (1 mL), and the reaction was stirred at rt for 16 h. When the TLC analysis showed full consumption of the starting material, the reaction mixture was filtered through Celite and evaporated. The crude product was purified by silica gel column chromatography using MeCN/H_2_O as a solvent. Isolation of the relevant fractions gave the title compound.

## Supplementary Material



## Notes

Experimental procedures, characterization data for all new compounds, and ^1^H and ^13^C NMR spectra of all new compounds (PDF)
