# Supplementary material for: Synthesis of Human Milk Oligosaccharides–Fluoroquinolone Conjugates and Investigation of Their Antimicrobial Activity
Source: Bioconjug Chem. 2026 Aug 20;37(9):1867–76. doi: 10.1021/acs.bioconjchem.6c00334 (PMC13587601; doi:10.1021/acs.bioconjchem.6c00334)
Supplement: Supplementary file 1 [file bc6c00334_si_001.pdf]

Synthesis of human milk oligosaccharides (HMOs)-fluoroquinolone conjugates and investigation of their antimicrobial activity

- Supporting Information -

Liza Nguyen-Van-Sang,[a] Emil Lindbäck[a] and Magne O. Sydnes[a, b*]

a: Department of Chemistry, Bioscience and Environmental Engineering, Faculty of Science and Technology, University of Stavanger, Stavanger NO-4036, Norway.

b: Department of Chemistry, University of Bergen, Bergen NO-5020, Norway.

Corresponding author: magne.sydnes@uib.no

General information 2

General procedure 3

Compounds analysis 4

^{1}H, ^{13}C and ^{19}F NMR spectra 22

References 54

Experimental Section

General Information

All chemicals were purchased from Sigma-Aldrich/Merck or VWR and used without any purification. Solvents were dried over 4 Å molecular sieves. The oligosaccharide **2´FL**, **3FL**, **LNT** and **LNnT** were generously provided by Inbiose (Belgium), as part of the "Stop Spread Bad Bugs" agreement and collaboration.

TLC visualization was achieved with UV light (254 and/or 365 nm) and via a mixture H_2SO_4/EtOH, 1:9 for oligosaccharides.

All purifications were performed through a silica-gel column chromatography with a hand pump and 230−400 mesh silica gel.

IR spectra were recorded on an Agilent Cary 630 FT-IR spectrophotometer. UV−vis spectra were obtained on an Agilent 8453 single-beam. Melting points (mp) were determined on a Stuart SMP20 melting point apparatus and are uncorrected.

NMR spectra were recorded on a Bruker Ascend 400 spectrometer (^{1}H: 400.13 MHz, ^{13}C: 100.61 MHz, ^{19}F: 376.46 MHz). Coupling constants (*J*) are given in Hz, and the multiplicity is reported as singlet (s), doublet (d), triplet (t), quartet (q), sextet (s), multiplet (m), and broad singlet (bs). Chemical shifts are reported in ppm and calibration is performed using the residual solvent signals. $CDCl_3$ (^{1}H: 7.26 ppm, ^{13}C: 77.16 ppm), MeOD (^{1}H: 3.31 ppm, ^{13}C 49.00 ppm), D_2O (^{1}H: 4.79 ppm) and $(CD_3)_2SO$ (^{1}H: 2.50 ppm, ^{13}C: 39.52 ppm).

General procedure

General procedure for CuAAC [3+2] cycloaddition (#1)

Copper (II) sulfate pentahydrate (1.2 equiv.) and sodium ascorbate (1.6 equiv.) were added to a stirred solution of azide (1 equiv.) and alkyne (1 equiv.) in dry DMF (2 mL) under inert atmosphere. The resulting reaction mixture was stirred at rt for 2 h, followed by dilution with EtOAc (5 mL). The organic phase was washed with 10% ww $Na_2S_2O_3$ aq until it was colourless (3 x 5 mL) and dried over $MgSO_4$. Filtration and concentration under reduce pressure gave the crude product that was subjected to purification by silica-gel column chromatography using pet. ether/EtOAc solvent system. Isolation of the relevant fractions gave the title compound.

General procedure for OAc deprotection (#2)

Oligosaccharide (1 equiv.) and MeONa (3 equiv.) were dissolved in dry MeOH (1 mL), and the reaction was stirred at rt overnight. When the TLC analysis showed full consumption of the starting material, the reaction mixture was filtered through celite and evaporated. The crude product was purified by silica-gel column chromatography using a MeCN/H_2O solvent system. Isolation of the relevant fractions gave the title compound.

Growth inhibition assay procedure[1]

Bacterial strains, *E. faecalis* (ATCC 29212), *E. coli* (ATCC 25922), *S. agalactiae* (ATCC 12386), *P. aeruginosa* (ATCC 27853) and *S. aureus* (ATCC 25923) were obtained from LGC Standards. Pathogens were transferred from biofreezer stocks onto blood agar plates by streaking for isolation while keeping the stock vials on ice throughout the procedure. The blood agar plates were incubated overnight at 37 °C and subsequently stored at 4 °C. Plates were used for up to two weeks, after which fresh blood agar plates were inoculated by transferring a loopful of bacterial colonies from the stored plates and incubated overnight at 37 °C. Following a maximum of four weeks of continuous maintenance on blood agar plates, fresh cultures were re-established by streaking directly from the original biofreezer stocks onto new blood agar plates. Bacterial colonies were transferred from a blood agar plate into 8 mL of the appropriate growth medium (Mueller–Hinton broth for *E. coli*, *P. aeruginosa* and *S. aureus* and brain heart infusion broth for *E. faecalis* and *S. agalactiae*). The cultures were incubated at 37 °C for 20 h. Test compounds were prepared as working stocks at twice the final test concentration, ensuring that the final DMSO concentration in the assay did not exceed 0.25%. Subsequently, 50 µL of each sample was dispensed in duplicate into the designated wells of a microtiter plate.

In addition, 50 µL of sterile deionized water (ddH_2O) was added to columns 1 and 12, while 50 µL of growth medium was added to column 1. Two milliliters of each bacterial culture were transferred into the corresponding 100 mL Erlenmeyer flask containing 25 mL of the appropriate growth medium. The diluted cultures were incubated at 37 °C with shaking at 100 rpm for 1.5 h for *E. faecalis*, *E. coli*, and *S. agalactiae*, or for 2.5 h for *P. aeruginosa* and *S. aureus*.

The optical density at 600 nm (OD_{600}) of each culture was measured using a 96-well microtiter plate, and the cultures were adjusted to the following target OD_{600} values: 0.125 for *E. faecalis*, 0.080 for *E. coli*, 0.075 for *P. aeruginosa*, 0.080 for *S. aureus*, and 0.080 for *S. agalactiae*.

A volume of 100 µL of the adjusted bacterial suspension was transferred into 9.9 mL of growth medium to obtain a 1:100 dilution. Subsequently, 4 mL of this suspension was transferred into 36 mL of growth medium to obtain a further 1:10 dilution.

A volume of 50 µL of the final bacterial suspension was dispensed into columns 2–11 of the designated microtiter plate according to Figure 1. For the gentamicin control plate, a two-fold serial dilution of gentamicin ranging from 32 µg/mL to 0.0156 µg/mL was prepared as illustrated in Figure 2. The transfer of bacterial suspensions into the microtiter plates was completed within 30 min to ensure consistent bacterial density across all wells.

The microtiter plates were incubated at 37 °C for 19–20 h. The microtiter plates were visually inspected. Results from plates showing growth in column 1 or no growth in column 12 were not considered valid and were excluded from analysis. These assays were repeated due to possible contamination or insufficient growth of the pathogen.

The absorbance of the wells was measured using the Victor plate reader. The plates were read at OD_{600}.

Reported protocol: Anti-bacterial growth inhibition assay.

Cytotoxicity assay

Cytotoxicity essays were performed against the melanoma, hepatocellular carcinoma (HepG2, ATCC, HB-8065) and non-malignant lung fibroblast cell line (MRC5, ATCC CCL-171) in an MTS in vitro cell proliferation essay (concentration ranging 1.56-100 µM). MRC5 was cultured in MEM Eagle (M7278) supplemented with 5 mL non-essential amino acid, 1 mM sodium

pyruvate and 0.15% (w/v) sodium bicarbonate. HepG2 was cultured in MEM Earle´s (F0325) supplemented with 5 mL non-essential amino acid (K0293) and 1 mM sodium pyruvate (L0473). In addition, 10% foetal bovine serum (FBS, S1810), 10 µg/mL Gentamicin (A2712) and 5 mL glutamine stable (200 mM per 500 mL medium) were added to both media. In a 96-wells plates (Nunclon Delta Surface VWR), MRC5 was seeded at 4000 cells/well and at 20,000 cells/well for HepG2. Following a 24 h incubation at 5% CO_2, 37 °C, the tested compounds were added to the wells (100 µL/well) and the media was replaced. Before assaying, MRC5 was incubated for 72 h and HepG2 for 24 h. In each well, 10 µL of CellTiter 96^R Aqueous One solution Reagent (G358B, Promega) were added and the plates were incubated for 1 h at 37 °C. The absorbance was then measured at 490 nm. Negative control (cell assayed in their corresponding media) and positive control (cell treated with 10% DMSO) were performed. Visualisation of data were performed using GraphPad Prism 8 4.2 and IC_{50} was calculated. This equation was used to calculate the percentage of surviving cells: Percent (%) cell survival: (absorbance treated wells - absorbance positive control)/(absorbance negative control - absorbance positive control) x 100

Compounds analysis

Per-*O*-acetyl-α-L-fucopyranosyl-(1→2)-β-D-galactopyranosyl-(1→4)-D-glucopyranosyl (1a)

2´Fucosyllactose (2.0 g, 4.10 mmol) and iodine (53 mg, 0.21 mmol) were dissolved in acetic anhydride (40 mL) and the resulting reaction mixture was stirred at rt for 18 h. The solvent was removed in *vacuo* and the crude mixture was purified by silica-gel column chromatography (pet. ether/EtOAc, 4:6). Isolation of the relevant fractions (R_f = 0.41, pet. ether/EtOAc, 4:6) yielded the title compound **1a** as a white foam (3.54 g, 95%, mp: 114-117 °C, ratio α/β is 64:36, according to ^{1}H NMR analysis).

IR (neat): ν_{max} 1738, 1366, 1210, 1037 cm^{-1}. **^{1}H NMR ($CDCl_3$):** δ 6.30 (d, J = 3.6 Hz, 0.64H-α), 5.68 (d, J = 8.1 Hz, 0.36H-β), 5.41 (t, J = 9.8 Hz, 0.7H), 5.36 (d, J = 4.1 Hz, 1H-α), 5.32-5.27 (m, 2H), 5.22-5.12 (m, 1.6H), 5.09 (dd, J = 9.7 Hz, 8.3 Hz, 0.45H), 5.04 (dd, J = 10.3 Hz, 3.7 Hz, 0.72H), 5.00-4.95 (m, 2H), 4.49-4.37 (m, 3H-β), 4.26 (dd, J = 5.2 Hz, 12.3 Hz, 1H), 4.18-4.05 (m, 4H), 3.90-3.80 (m, 3H), 2.17 (s, 2H), 2.16 (s, 3H), 2.14 (s, 2H), 2.13 (s, 1H), 2.12 (s, 3H), 2.10 (s, 1H), 2.08 (s, 1H), 2.07-2.06 (m, 4H), 2.04 (s, 3H), 2.01 (s, 2H), 2.00 (s, 2H), 1.99 (s, 1H), 1.97-1.96 (m, 5H), 1.27-1.22 (m, 3H). **^{13}C NMR ($CDCl_3$):** δ 171.27, 170.83, 170.80, 170.71, 170.65, 170.47, 170.42, 170.25, 170.12, 170.09, 169.91, 169.86, 169.47, 168.91, 168.87, 166.52, 100.27, 100.05, 95.78, 91.87, 89.08, 73.98, 73.93, 73.76, 73.51, 73.41, 71.75, 71.22, 71.19, 70.98, 70.87, 70.48, 70.41, 69.26, 69.18, 68.20, 68.12, 67.63,

67.45, 67.12, 65.09, 64.98, 62.22, 62.05, 61.09, 60.92, 60.51, 22.30, 21.17, 20.99, 20.95, 20.92, 20.84, 20.77, 20.74, 20.71, 20.70, 20.60, 15.66, 15.53, 14.32. **HRMS (ESI):** Calculated for $C_{38}H_{52}NaO_{25}^+$ [M + Na$^+$] 931.2690, found 931.2693.

Per-*O*-acetyl-α-L-fucopyranosyl-(1→3)-[β-D-galactopyranosyl-(1→4)]-D-glucopyranosyl (1b)

3-Fucosyllactose (2.0 g, 4.1 mmol) and iodine (53 mg, 0.21 mmol) were stirred in acetic anhydride (40 mL) at rt for 24 h. The solvent was removed in *vacuo* and the crude mixture was purified by silica-gel column chromatography (pet. ether/EtOAc, 3:7). Isolation of the relevant fractions (R_f = 0.2, pet. ether/EtOAc, 1:1) yielded the title compound **1b** as a white foam (3518 mg, 94%, mp: 56-59 °C, ratio α/β is 52:48, according to ^{1}H NMR analysis.).

IR (neat): ν_{max} 1739, 1368, 1209, 1016 cm^{-1}. **^{1}H NMR (CDCl$_3$):** δ 6.18 (d, J = 3.7 Hz, 0.52H-α), 5.51 (d, J = 8.4, 0.48 H-β), 5.42-5.32 (m, 3H-α), 5.18 (dd, J = 11.1 Hz, 3.2 Hz, 1H), 5.13-5.08 (m, 1H), 5.05-4.99 (m, 2H), 4.98-4.95 (m, 1H), 4.94-4.92 (m, 0.6 H), 4.59-4.51 (m, 2H), 4.46 (dd, J = 10.7 Hz, 8.1 Hz, 1H-β), 4.32-4.27 (m, 1H), 4.14-4.06 (m, 2H), 4.03-3.84 (m, 3H), 3.62-3.58 (m, 0.4H), 2.19 (s, 3H), 2.14 (s, 6H), 2.07 (s, 6H), 2.05 (s, 3H), 2.00 (s, 6H), 1.97 (s, 3H), 1.96 (s, 3H), 1.27-1.22 (d, J = 6.53 Hz, 3H). **^{13}C NMR (CDCl$_3$):** δ 171.24, 170.88, 170.68, 170.64, 170.50, 170.29 (2 x C), 170.11, 170.07, 169.93, 169.90, 169.66, 169.25, 169.19, 169.09, 169.03, 100.74, 100.69, 95.68, 95.61, 92.03, 89.15, 74.10, 74.02, 73.75, 73.55, 72.77, 72.44, 71.48, 71.42, 71.16, 71.13, 71.05, 69.11, 68.94, 68.19, 68.17, 68.08, 68.00, 66.70, 64.39, 64.32, 61.39, 61.17, 60.75, 60.69, 60.49, 21.15, 21.05, 20.90, 20.85, 20.76, 20.71, 20.62, 15.92, 15.89, 14.30. **HRMS (ESI):** Calculated for $C_{38}H_{52}NaO_{25}^+$ [M + Na$^+$] 931.2690, found 931.2691.

2,3,4,-*O*-acetyl-α-L-fucopyranosyl-(1→2)-3,4,6-*O*-acetyl-β-D-galactopyranosyl-(1→4)-2,3,6-*O*-acetyl-D-glucopyranose (2a)

Glycosyl acetate **1a** (3.5 g, 3.9 mmol) and hydrazine acetate (711 mg, 7.72 mmol) were dissolved in dry THF (30 mL), and the resulting reaction mixture was stirred at rt for 40 h. The reaction was quenched by adding water (15 mL) followed by extraction with EtOAc (2 x 20 mL). The combined organic layers were washed with saturated aqueous $NaHCO_3$ (2 x 20 mL), dried over $MgSO_4$, filtered and concentrated under reduced pressure. The crude product was purified by silica-gel column chromatography (pet. ether/EtOAc, 3:7). Isolation of the relevant fractions (R_f = 0.42, pet. ether/EtOAc, 3:7) yielded the title compound **2a** as a white foam (2.06 g, 62%, mp: 127–130 °C, ratio α/β is 74:26 according to ^{1}H NMR analysis).

IR (neat): ν_{max} 3473, 1736, 1367, 1214, 1033 cm^{-1}. **^{1}H NMR (CDCl$_3$):** δ 5.49 (t, J = 9.8 Hz, 1H), 5.42 (t, J = 3.5 Hz, 0.74H-α), 5.36 (d, J = 3.2 Hz, 1H-α), 5.15-5.11 (m, 2H), 5.0-4.94 (m, 2H), 4.87-4.82 (m, 1H), 4.72 (t, J = 8.1 Hz, 0.26H-β), 4.52-4.38 (m, 4H-β), 4.29-4.12 (m, 3H), 4.06 (dd, J = 7.1 Hz, 10.2 Hz, 1H), 3.86-3.79 (m, 3H), 3,71-3.57 (m, 1H), 2.15 (s, 6H), 2.11 (s, 3H), 2.08 (s, 3H), 2.06 (m, 6H), 1.99 (s, 3H), 1.97-1.96 (m, 6H), 1.20 (m, 3H). **^{13}C NMR (CDCl$_3$):** δ 170.92, 170.85, 170.82, 170.50, 170.38, 170.31, 170.27, 170.21, 170.14, 170.08, 170.00, 169.86, 100.33, 100.22, 95.67, 90.21, 74.47, 73.57, 73.40, 73.25, 71.49, 71.23, 71.10, 70.89, 70.82, 69.04, 68.54, 68.17, 67.74, 67.57, 67.20, 65.02, 62.47, 62.28, 61.04, 21.02, 21.00, 20.89, 20.82, 20.79, 20.76, 20.74, 15.66, 15.52. **HRMS (ESI):** Calculated for $C_{36}H_{50}NaO_{24}^{+}$ [M + Na$^+$] 889.2584, found 889.2592.

2,3,4-*O*-acetyl-α-L-fucopyranosyl-(1→3)-[2,3,4,6-*O*-acetyl-β-D-galactopyranosyl-(1→4)]-2,6-*O*-acetyl-D-glucopyranose (2b)

Glycosyl acetate **1b** (3.5 g, 3.8 mmol) and hydrazine acetate (700 mg, 7.6 mmol) were dissolved in dry THF (30 mL) and the reaction was stirred at rt for 53 h. The reaction was quenched by the addition of water (15 mL). Subsequently, the mixture was extracted with EtOAc (1 x 20 mL). The combined organic layers were washed with saturated aqueous $NaHCO_3$ (2 x 20 mL) and dried over $MgSO_4$. After filtration and concentration, the crude product was purified by silica-gel column chromatography (pet. ether/EtOAc, 3:7). Isolation of the relevant fractions (R_f = 0.3, pet. ether/EtOAc, 3:7) yielded the title compound **2b** as a white foam (2280 mg, 69%, mp: 131-134 °C, ratio α/β is 72:28 according to ^{1}H NMR analysis).

IR (neat): ν_{max} 3474, 1736, 1366, 1211, 1036 cm^{-1}. **^{1}H NMR (CDCl$_3$):** δ 5.42-5.37 (m, 3H-α), 5.29 (d, J = 3.5 Hz, 0.72 H-α), 5.21-5.15 (m, 1H), 5.11-5.03 (m, 2H), 4.99-4.92 (m, 2H), 4.86-4.81 (m, 1H), 4.60 (dd, J = 12.2 Hz, 1.6 Hz, 1H), 4.56-4.52 (m, 1H + 0.28H-β), 4.50 (d, J = 8.1 Hz, 1H-β), 4.31-4.19 (m, 2H), 4.10-4.01 (m, 2H), 3.88-3.83 (m, 2H), 3.13 (bs, 1H), 2.19 (s, 3H), 2.16 (s, 3H), 2.14 (s, 3H), 2.08 (s, 3H), 2.06 (s, 3H), 2.05 (s, 3H), 2.01 (s, 3H), 1.97 (s, 3H), 1.95 (s, 3H), 1.22 (d, J = 6.7 Hz, 3H). **^{13}C NMR (CDCl$_3$):** δ 171.48, 171.34, 171.09, 170.96, 170.90, 170.856, 170.70, 170.61, 170.54, 170.50, 170.18, 170.12, 169.95, 169.17, 169.13, 100.76, 100.69, 95.82, 95.52, 89.94, 74.41, 74.20, 74.09, 73.79, 73.52, 71.54, 71.43, 71.17, 71.12, 71.05, 69.09, 69.03, 68.74, 68.32, 68.29, 68.13, 66.77, 64.35, 64.17, 61.69, 61.53, 60.72, 60.54, 21.17, 21.12, 21.00, 20.90, 20.87, 20.80, 20.78, 20.75, 20.65, 15.90, 14.31. **HRMS(ESI):** Calculated for $C_{36}H_{50}NaO_{24}^{+}$ [M + Na$^+$] 889.2584, found 889.2592.

2,3,4,-*O*-acetyl-α-L-fucopyranosyl-(1→2)-3,4,6-*O*-acetyl-β-D-galactopyranosyl-(1→4)-2,3,6-*O*-acetyl-α-D-glucopyranosyl-trichloroacetimidate (3a)

A mixture of compound **2a** (2.0 g, 2.3 mmol), trichloro acetonitrile (0.5 mL, 4.7 mmol) and cesium carbonate (77 mg, 0.2 mmol) dissolved in DCM (30 mL) was stirred at rt for 15 h. After this time, the solvent was removed under reduced pressure, and the residue underwent purification by silica-gel column chromatography (pet. ether/EtOAc, 4:6). Isolation of the relevant fractions (R_f = 0.45, pet. ether/EtOAc, 4:6) yielded the title compound **3a** as a white foam (*α*-selective, 2.10 g, 89%, mp: 115-118 °C, ratio α/β is >99:1 according to ^{1}H NMR analysis).

$[\boldsymbol{\alpha}]_D^{27\,°C}$ -16 (c 0.25, MeOH). **IR (neat):** ν_{max} 1741, 1366, 1213, 1040, 796 cm^{-1}. **^{1}H NMR (CDCl$_3$):** *δ* 8.68 (s, 1H), 6.56 (d, *J* = 3.6Hz, 1H-α), 5.52 (t, *J* = 9.8 Hz, 1H), 5.38 (d, *J* = 3.7 Hz, 1H-α), 5.28 (d, *J* = 2.8 Hz, 1H), 5.21 (d, *J* = 2.6 Hz, 1H), 5.14-5.06 (m, 2H), 5.00-4.92 (m, 2H), 4.55 (dd, *J* = 12.2 Hz, 1.7 Hz, 1H), 4.48 (qt, *J* = 6.5 Hz, 1H), 4.42 (d, *J* = 7.6 Hz, 1H-β), 4.29-4.15 (m, 3H), 4.10-4.04 (m, 1H), 3.93 (t, *J* = 9.7 Hz, 1H), 3.88-3.83 (m, 2H), 2.14 (s, 3H), 2.12 (s, 6H), 2.07 (s, 3H), 2.06 (s, 3H), 2.01 (s, 3H), 1.98 (s, 3H), 1.97 (s, 3H), 1.94 (s, 3H), 1.24 (d, *J* = 6.5 Hz, 3H). **^{13}C NMR (CDCl$_3$):** *δ* 170.88, 170.68, 170.55, 170.43, 170.27, 170.13 (2 x C), 169.91, 169.79, 160.91, 100.32, 95.58, 92.87, 90.83, 73.52 (2 x C), 71.25, 71.23, 71.11, 70.93, 69.71, 69.28, 68.21, 67.52, 67.17, 65.11, 61.85, 60.91, 20.97, 20.90, 20.78 (2 x C), 20.76 (2 x C), 20.74 (2 x C), 20.60, 15.50. **HRMS (ESI):** Calculated for $C_{38}H_{50}Cl_3NNaO_{24}^+$ [M + Na$^+$] 1032.1681, found 1032.1665.

2,3,4-*O*-acetyl-α-L-fucopyranosyl-(1→3)-[2,3,4,6-*O*-acetyl-β-D-galactopyranosyl-(1→4)]-2,6-*O*-acetyl-α-D-glucopyranosyl-trichloroacetimidate (3b)

A mixture of compound **2b** (2.3 g, 2.6 mmol), trichloro acetonitrile (0.5 mL, 5.2 mmol) and cesium carbonate (85 mg, 0.3 mmol) in DCM (30 mL) was stirred at rt for 17 h. The solvent mixture was evaporated, and the crude residue was purified by silica-gel column chromatography (pet. ether/EtOAc, 4:6). Isolation of the relevant fractions (R_f = 0.4, pet. ether/EtOAc, 4:6) yielded the title compound **3b** as a white foam (*α*-selective, 2.19 g, 83%, mp: 121-124 °C, ratio α/β is >99:1 according to ^{1}H NMR analysis).

$[\boldsymbol{\alpha}]_D^{25.1°C}$ -21.2 (c 0.47, CHCl$_3$). **IR (neat):** ν_{max} 3344, 1735, 1368, 1213, 1025 cm^{-1}. **^{1}H NMR (CDCl$_3$):** *δ* 8.61 (s, 1H), 6.40 (d, *J* = 4.2 Hz, 1H-α), 5.45 (d, *J* = 3.8 Hz, 1H-α), 5.42 (d, *J* = 2.8 Hz, 1H), 5.4 (d, *J* = 2.6 Hz, 1H), 5.19 (dd, *J* = 10.9 Hz, 3.3 Hz, 1H), 5.13-5.05 (m, 3H), 5.03-4.98 (m, 1H), 4.97-4.94 (m, 1H), 4.59 (d, *J* = 12.8 Hz, 1H), 4.55 (app. t, *J* = 5.8 Hz, 1H), 4.49 (d, *J* = 7.9 Hz, 1H-β), 4.30 (dd, *J* = 11.4 Hz, 8.1 Hz, 1H), 4.22-4.18 (m, 1H), 4.09 (d, *J* = 10.9 Hz, 1H), 3.95 (d, *J* = 5.7 Hz, 2H), 3.87 (t, *J* = 7.1 Hz, 1H), 2.19 (s, 3H), 2.16 (s, 3H), 2.15 (s, 3H), 2.12 (s, 3H), 2.08 (s, 3H), 2.04 (s, 3H), 2.01 (s, 3H), 1.97 (s, 3H), 1.96 (s, 3H), 1.26 (d, *J* = 6.6 Hz, 3H). **^{13}C NMR (CDCl$_3$):** *δ* 170.88, 170.83, 170.66, 170.49, 170.27, 170.21, 170.11, 169.96, 169.17, 161.01, 100.79, 95.74, 92.99, 90.86, 73.59, 73.01, 71.81, 71.56, 71.43, 71.14 (2 x C), 69.02, 68.25, 68.21, 66.71, 64.33, 61.23, 60.67, 20.90, 20.89, 20.85,

20.77 (2 x C), 20.74, 20.71, 20.66, 20.63, 15.93. **HRMS (ESI):** Calculated for $C_{38}H_{50}Cl_3NNaO_{24}^+$ [M + Na$^+$] 1032.1681, found 1032.1660.

2,3,4,-*O*-acetyl-α-L-fucopyranosyl-(1→2)-3,4,6-*O*-acetyl-β-D-galactopyranosyl-(1→4)-2,3,6-*O*-acetyl-β-D-glucopyranosyl-2-azido ethyl (4a)

10 drops of TMSOTf were added to a solution of **3a** (2.1 g, 2.1 mmol) and 2-azidoethanol **5** (360 mg, 4.1 mmol) in dry DCM (40 mL), under an inert atmosphere. The reaction mixture was stirred at rt for 1 h. When TLC indicates complete conversion of the starting material (1h), the reaction was quenched with a few drops of NEt_3 and the mixture was stirred for an additional 10 minutes. Subsequently, the mixture was filtered through celite and concentrated under reduce pressure. The residue was purified by silica-gel column chromatography (pet. ether/EtOAc, 1:1) and the isolation of the relevant fractions (R_f = 0.23, pet. ether/EtOAc, 1:1) yielded the title compound **4a** as a white foam (*β*-selective, 810 mg, 42%. Mp: 94-97 °C, ratio α/β is 1:>99 according to ^{1}H NMR analysis).

$[\alpha]_D^{26.6\ °C}$ -67.9 (c 0.53, MeOH). **IR (neat):** ν_{max} 2104, 1737, 1366, 1213, 1035 cm^{-1}. **^{1}H NMR ($CDCl_3$):** *δ* 5.37 (d, *J* = 3.7 Hz, 1H-α), 5.31 (d, *J* = 2.5 Hz, 1H), 5.27 (d, *J* = 2.5 Hz, 1H), 5.14-5.09 (m, 2H), 5.00-4.94 (m, 3H), 4.57-4.51 (m, 2H-β), 4.42-4.39 (m, 2H-β), 4.25 (dd, *J* = 12.2 Hz, 5.4 Hz, 1H), 4.16-4.06 (m, 2H), 4.04-3.99 (m, 1H), 3.87-3.80 (m, 3H), 3.74-3.68 (m, 1H), 3.64-3.61 (m, 1H), 3.52-3.46 (m, 1H), 3.30-3.25 (m, 1H), 2.15 (s, 3H), 2.14 (s, 3H), 2.11 (s, 3H), 2.06-2.04 (m, 9H), 1.99 (s, 3H), 1.97 (s, 6H), 1.20 (d, *J* = 6.4, 3H). **^{13}C NMR ($CDCl_3$):** *δ* 170.84, 170.82, 170.75, 170.48, 170.27, 170.26, 170.15, 169.92, 169.61, 100.95, 100.38, 95.64, 74.38, 73.57, 73.27, 72.11, 71.41, 71.18, 71.11, 70.90, 68.96, 68.12, 67.58, 67.18, 64.99, 62.14, 61.02, 50.65, 20.99, 20.89, 20.85, 20.82, 20.78, 20.79 (2 x C), 20.77, 20.74, 15.67. **HRMS (ESI):** Calculated for $C_{38}H_{53}N_3NaO_{24}^+$ [M + Na$^+$] 958.2911, found 958.2917.

2,3,4-*O*-acetyl-α-L-fucopyranosyl-(1→3)-[2,3,4,6-*O*-acetyl-β-D-galactopyranosyl-(1→4)]-2,6-*O*-acetyl-β-D-glucopyranosyl-2-azido ethyl (4b)

10 drops of TMSOTf were added to a solution of **3b** (2.2 g, 2.1 mmol) and 2-azidoethanol **5** (372 mg, 4.3 mmol) in dry DCM (40 mL), at rt and under an inert atmosphere. The reaction mixture was stirred at rt for 1 h before quenching with a few drops of NEt_3 and kept the reaction stirring for an additional 10 minutes. Subsequently, the mixture was filtered through celite and concentrated under reduced pressure. The residue was purified by silica-gel column chromatography (pet. ether/EtOAc, 1:1) and the isolation of the relevant fractions (R_f = 0.4, pet. ether/EtOAc, 4:6) yielded the title compound **4b** as a white foam (*β*-selective, 1.11 g, 56%, mp: 94-97 °C, ratio α/β is 1:>99 according to ^{1}H NMR analysis).

$[\alpha]_D^{26.8°C}$ -50 (c 0.12, $CHCl_3$). **IR (neat):** ν_{max} 2103, 1736, 1368, 1210, 1041 cm^{-1}. **^1H NMR ($CDCl_3$):** δ 5.39 (d, *J* = 3.5 Hz, 1H), 5.37 (d, *J* = 3.1 Hz, 1H), 5.30 (d, *J* = 3.8 Hz, 1H-α), 5.16 (dd, *J* = 10.9 Hz, 3.1 Hz, 1H), 5.09-5.02 (m, 3H), 4.99-4.92 (m, 2H), 4.65 (dd, *J* = 12.2 Hz, 1.9 Hz, 1H), 4.52 (dd, *J* = 11.5 Hz, 6.4 Hz, 1H), 4.47 (d, *J* = 8.1 Hz, 1H-β), 4.35 (d, *J* = 8.0 Hz, 1H-β), 4.28 (dd, *J* = 11.4 Hz, 7.1 Hz, 1H), 4.05 (dd, *J* = 12.3 Hz, 4.3 Hz, 1H), 3.95-3.84 (m, 4H), 3.61-3.55 (m, 1H), 3.47-3.38 (m, 2H), 3.27 (ddd, *J* = 13.3 Hz, 4.7 Hz, 3.5 Hz, 1H), 2.17 (s, 3H), 2.13 (s, 3H), 2.12 (s, 3H), 2.06 (s, 3H), 2.04 (s, 3H), 2.03 (s, 3H), 2.02 (s, 3H), 1.95 (s, 3H), 1.93 (s, 3H), 1.21 (d, *J* = 6.6 Hz, 3H). **^{13}C NMR ($CDCl_3$):** δ 171.28, 170.87, 170.70, 170.54, 170.31, 170.08, 169.90, 169.69, 169.05, 100.90, 100.73, 95.63, 74.14, 74.11, 73.43, 73.41, 71.45, 71.11, 71.05, 69.06, 68.14, 68.08, 67.82, 66.73, 64.29, 61.31, 60.78, 50.74, 21.03, 20.92, 20.89, 20.87, 20.83, 20.75, 20.70 (2 x C), 20.62, 15.89. **LC-MS (ESI):** Calculated for $C_{38}H_{53}N_3NaO_{24}^+$ [M + Na^+] 958.2911, found 958.

2-azidoethanol (5b)

Sodium azide (1.2 g, 18.4 mmol) was added to a solution of 2-bromoethanol (1 mL, 15.3 mmol) in DMF (6 mL). The resultant mixture was kept stirring at 50 °C for 20 h under inert atmosphere. Afterward, it was allowed to reach rt before H_2O was added. The mixture was extracted with EtOAc (3 x 10 mL), and the combined organic layers were washed with H_2O (10 mL), brine (10 mL), then dried over $MgSO_4$. Filtration and removal of the solvent under reduced pressure gave the crude product which was purified by silica-gel column chromatography (pet. ether/EtOAc, 1:1). Concentration of the relevant fractions (R_f = 0.6, pet. ether/EtOAc, 1:1) gave the title compound **5b** as a slightly yellow oil (562 mg, 55%).

IR (neat): ν_{max} 3360, 2932, 2879, 2092 cm^{-1}. **^1H NMR ($CDCl_3$):** δ 3.77 (t, *J* = 4.8 Hz, 2H), 3.44 (t, *J* = 5.1 Hz, 2H). **^{13}C NMR ($CDCl_3$):** δ 61.65, 53.67. The spectroscopic data are in accordance with data previously reported in the literature.[2]

1-cyclopropyl-6-fluoro-4-oxo-7-(4-(prop-2-yn-1-yl)piperazin-1-yl)-1,4-dihydroquinoline-3-carboxylic acid (6)

A mixture of ciprofloxacin (200 mg, 0.6 mmol), propargyl bromide (0.1 mL, 0.7 mmol) and sodium bicarbonate (60 mg, 0.7 mmol) in MeCN (10 mL) was kept stirring at 80 °C for 15 h. After this time, the mixture was filtered through cotton and washed with a solution of DCM/MeOH (1:1, 40 mL). The filtrate was concentrated under reduced pressure and the residue was purified by silica-gel column chromatography (DCM/MeOH, 96:4). Isolation of the relevant fractions (R_f = 0.40, DCM/MeOH, 96:4) yielded the title compound **6** as a yellow powder (53 mg, 25%, mp: 242-245°C[3]).

IR (neat): νmax 3249, 2843, 1902, 1733, 1626, 1450, 1254 cm^{-1}. **^{1}H NMR ($CDCl_3$)**: δ 14.99 (bs, OH), 8.77 (s, 1H), 8.02 (d, J = 12.7 Hz, 1H), 7.37 (d, *J* = 6.9 Hz, 1H), 3.56-3.49 (m, 1H), 3.41 (d, *J* = 2.3 Hz, 2H), 3.39 (t, *J* = 4.9 Hz, 4H), 2.82 (t, *J* = 4.7 Hz, 4H), 2.31 (t, *J* = 2.2 Hz, 1H), 1.39 (q, *J* = 7.2 Hz, 2H), 1.20 (m, 2H). **^{13}C NMR ($CDCl_3$):** δ 177.30, 167.17, 153.66 (d, *J* = 251.3 Hz), 147.58, 145.98 (d, *J* = 10.5 Hz), 139.23, 120.07 (d, *J* = 7.8 Hz), 112.66 (d, *J* = 23.4 Hz), 108.35, 104.96 (d, *J* = 2.5 Hz), 78.33, 73.84, 51.58, 49.87, 46.07, 35.40, 8.38. **^{19}F NMR ($CDCl_3$):** δ -120.71 – -120.76 (m, 1F). The spectroscopic data are in accordance with data previously reported in the literature.[4]

5-amino-1-cyclopropyl-7-(3,5-dimethyl-4-(prop-2-yn-1-yl)piperazin-1-yl)-6,8-difluoro-4-oxo-1,4-dihydroquinoline-3-carboxylic acid (7)

A mixture of sparfloxacin (1.0 g, 2.5 mmol), propargyl bromide (0.3 mL, 3.3 mmol) and sodium bicarbonate (277 mg, 3.3 mmol) in MeCN (20 mL) was kept stirring at 80 °C for 17 h. After this time, the mixture was filtered through cotton and washed with a solution of DCM/MeOH (1:1, 40 mL). The filtrate was concentrated under reduced pressure and the residue was purified by silica-gel column chromatography (DCM/MeOH, 96:4). Isolation of the relevant fractions (R_f = 0.61, DCM/MeOH, 96:4) yielded the title compound **7** as a yellow powder (500 mg, 46%, mp: 237-240 °C).

IR (neat): ν_{max} 3443, 3329, 2975, 2842, 3276, 1987, 1712, 1630, 1432, 1375, 1289 cm^{-1}. **^{1}H NMR (($CD_3)_2SO$):** *δ* 14.70 (bs, OH), 8.50 (s, 1H), 7.25 (bs, NH_2), 4.04-4.00 (m, 1H), 3.60 (d, *J* = 2.1 Hz, 2H), 3.11 (t, *J* = 2.0 Hz, 1H), 2.98 (t, *J* = 11.3 Hz, 2H), 2.75-2.71 (m, 2H), 1.12-1.07 (m, 4H), 1.01 (d, *J* = 6.2 Hz, 6H). Two protons are overlapping with the signal of the DMSO-d6 solvent, determined by ^{1}H-^{13}C HSQC. **^{13}C NMR (($CD_3)_2SO$):** *δ* 179.64 (determined by ^{1}H-^{13}C HMBC), 165.52, 150.07, 136.47, 136.34, 127.07 (determined by ^{1}H-^{13}C HMBC), 105.58, 105.07, 77.34, 75.86, 57.36, 53.08, 40.65, 36.23, 16.80, 8.47. Two carbons are proven by presence of signals in the ^{19}F spectra. **^{19}F NMR (($CD_3)_2SO$):** *δ* -144.80 (s, 1F), -147.85 (s, 1F). **HRMS (ESI):** Calculated for $C_{22}H_{25}F_2N_4O_3^+$ [M + H^+] 431.1889, found 431.1899.

2,3,4,-*O*-acetyl-α-L-fucopyranosyl-(1→2)-3,4,6-*O*-acetyl-β-D-galactopyranosyl-(1→4)-2,3,6-*O*-acetyl-β-D-glucopyranosyl-1-cyclopropyl-7-(4-((1-methyl-1*H*-1,2,3-triazol-4-yl)methyl)piperazin-1-yl)-6-fluoro-4-oxo-1,4-dihydroquinoline-3-carboxylic acid (8a)

Copper (II) sulfate pentahydrate (19 mg, 0.12 mmol) and sodium ascorbate (30 mg, 0.15 mmol) were added to a stirred solution of azide **4a** (100 mg, 0.10 mmol) and alkyne **6** (44 mg, 0.12 mmol) in dry DMF (2 mL) and at rt for 1 h, according to procedure #1. Purification by silica-gel column chromatography (MeCN/H_2O, 95:5) yielded the title compound **8a** (R_f = 0.26, MeCN/H_2O, 95:5) as an orange solid (104 mg, 80%, mp: 156-159 °C).

$[\alpha]_D^{26.8}$ °C -28.5 (c 0.14, MeOH). **IR (neat):** ν_{max} 2955, 2825, 1966, 1740, 1625, 1456, 1367, 1213, 1132, 1038 cm^{-1}. **^{1}H NMR (CD$_3$OD):** δ 8.71 (s, 1H), 7.95 (s, 1H), 7.80 (d, J = 13.0 Hz, 1H), 7.54 (d, J = 6.5 Hz, 1H), 5.34 (d, J = 2.9 Hz, 1H), 5.32 (d, J = 3.8 Hz, 1H-α), 5.25 (d, J = 3.6 Hz, 1H), 5.18 (dd, J = 10.9 Hz, 3.3 Hz, 1H), 5.10 (m, 2H), 4.90 (d, J = 3.7 Hz, 1H), 4.87-4.82 (m, 1H), 4.67 (d, J = 7.8 Hz, 1H-β), 4.62-4.61 (m, 2H), 4.59 (d, J = 7.8 Hz, 1H-β), 4.54 (d, J = 11.1 Hz, 1H), 4.48 (q, J = 6.4 Hz, 1H), 4.30 (dd, J = 11.8 Hz, 5.8 Hz, 1H), 4.21-4.11 (m, 3H), 4.09-4.06 (m, 2H), 3.89-3.82 (m, 2H), 3.80 (d, J = 4.2 Hz, 2H), 3.74 (dd, J = 9.8 Hz, 7.7 Hz, 2H), 3.42 (app. as singlet, 4H), 2.77 (app. as singlet, 4H), 2.13 (s, 3H), 2.12 (s, 3H), 2.11 (s, 3H), 2.03 (s, 3H), 2.02 (s, 3H), 1.99 (s, 3H), 1.95 (s, 6H), 1.95 (s, 3H), 1.40 (d, J = 4.1 Hz, 2H), 1.22 (app. as singlet, 2H), 1.19 (d, J = 6.5 Hz, 3H). **^{13}C NMR (CD$_3$OD):** δ 178.04, 172.51, 172.31, 172.06, 171.98, 171.93, 171.76, 171.62, 171.40, 171.02, 169.92, 154.92 (d, J = 253.2 Hz), 149.08, 146.85 (d, J = 10.8 Hz), 144.31, 140.65, 126.16, 120.92, 112.54 (d, J = 23.5 Hz), 107.07, 101.99, 101.46, 96.92, 75.89, 75.40, 74.97, 74.23, 73.65, 73.21, 72.43, 72.31, 71.71, 69.56, 69.19, 68.93, 68.77, 66.20, 63.69, 62.16, 53.67, 53.54 51.49, 50.68, 50.63, 36.82, 21.0, 20.96, 20.91, 20.79, 20.63, 20.60, 20.57, 20.53, 20.48, 15.90, 8.65, 8.54. **^{19}F NMR (CD$_3$OD):** δ -122.5 (s, 1F). **HRMS (ESI):** Calculated for $C_{58}H_{74}FN_6O_{27}$ [M + H$^+$] 1305.4508; found 1305.4583.

2,3,4,-*O*-acetyl-α-L-fucopyranosyl-(1→2)-3,4,6-*O*-acetyl-β-D-galactopyranosyl-(1→4)-2,3,6-*O*-acetyl-β-D-glucopyranosyl-5-amino-1-cyclopropyl-6,8-difluoro-7-(4-((1-methyl-1*H*-1,2,3-triazol-4-yl)methyl)-3,5-dimethylpiperazin-1-yl)-4-oxo-1,4-dihydroquinoline-3-carboxylic acid (8b)

Copper (II) sulfate pentahydrate (19 mg, 0.12 mmol) and sodium ascorbate (30 mg, 0.15 mmol) were added to a stirred solution of azide **4a** (100 mg, 0.10 mmol) and alkyne **7** (52 mg, 0.12 mmol) in dry DMF (2 mL) and stirred at rt for 2 h, following procedure #1. Purification by silica-gel column chromatography (MeCN/H_2O, 95:5 – 9:1) the title compound **8b** (R_f = 0.45, MeCN/H_2O, 97:3) as an orange solid (90 mg, 66%, mp: 153-156 °C).

$[\alpha]_D^{27.1}$ °C -61.5 (c 0.13, MeOH). **IR (neat):** ν_{max} 3466, 2966, 2878, 1964, 1741, 1629, 1436, 1367, 1215, 1133, 1039 cm^{-1}. **^{1}H NMR (CD$_3$OD):** δ 8.51 (s, 1H), 7.95 (s, 1H), 5.34 (d, J = 2.9 Hz, 1H), 5.32 (d, J = 3.8 Hz, 1H-α), 5.25 (d, J = 3.4 Hz, 1H), 5.18 (dd, J = 10.9 Hz, 3.3 Hz, 1H), 5.20-5.09 (m, 2H), 4.90-4.82 (m, 3H), 4.71 (d, J = 7.9 Hz, 1H-β), 4.63 (t, J = 5.1 Hz, 2H), 4.57 (d, J = 7.6 Hz, 1H-β), 4.53 (d, J = 11.7 Hz, 1H), 4.47 (q, J = 6.7 Hz, 1H), 4.29-4.25 (m, 1H), 4.20 (s, 2H), 4.17-4.11 (m, 2H), 4.09-4.03 (m, 2H), 3.94-3.93 (m, 1H), 3.87-3.85 (m, 2H), 3.74 (dd, J = 9.9 Hz, 7.6 Hz, 1H), 3.37 (d, J = 11.1 Hz, 2H), 3.13-3.06 (m, 2H), 2.70-2.68 (m, 2H), 2.14 (s, 3H), 2.10 (s, 3H), 2.10 (s, 3H), 2.03 (s, 3H), 2.05 (s, 3H), 1.95 (s, 3H), 1.95 (s, 3H), 1.95 (s, 3H), 1.94 (s, 3H), 1.31 (dd, J = 5.9 Hz, 3.3 Hz, 6H), 1.18 (d, J = 6.5 Hz, 3H), 1.15 (app. as singlet, 2H), 1.08 (app. as singlet, 2H). **^{13}C NMR (CD$_3$OD):** δ 181.32,

172.45, 172.31, 172.05, 171.97, 171.92, 171.73, 171.61, 171.39, 171.03, 169.10, 151.08, 142.07, 140.92 (dd, *J* = 231.1 Hz, 4.8 Hz), 137.89 (d, *J* = 12.7 Hz), 137.46 (dd, *J* = 237.7 Hz, 5.4 Hz), 135.07 (dd, *J* = 13.4 Hz, 10.9 Hz), 129.38 (d, *J* = 4.3 Hz), 126.35, 106.69, 101.95, 101.43, 96.90, 75.90, 74.97, 74.27, 73.63, 73.19, 72.45, 72.30, 71.69, 69.58, 69.19, 68.93, 68.77, 66.20, 63.75, 62.17, 58.86, 54.99, 54.81, 54.78, 51.34, 42.24, 41.97, 41.82, 21.09, 20.96, 20.91, 20.73, 20.65, 20.62, 20.59, 20.50 (2 x C), 17.64, 17.62, 15.83, 9.48 (d, *J* = 5.5 Hz), 9.40 (d, *J* = 5.5 Hz). **^{19}F NMR (CD_3OD):** δ -145.42 (s, 1F), -149.76 (s, 1F). **HRMS (ESI):** Calculated for $C_{60}H_{78}F_2N_7O_{27}$ [M + H$^+$] 1366.4908, found 1366.4904.

2,3,4-*O*-acetyl-α-L-fucopyranosyl-(1→3)-[2,3,4,6-*O*-acetyl-β-D-galactopyranosyl-(1→4)]-2,6-*O*-acetyl-β-D-glucopyranosyl-1-cyclopropyl-7-(4-((1-methyl-1*H*-1,2,3-triazol-4-yl)methyl)piperazin-1-yl)-6-fluoro-4-oxo-1,4-dihydroquinoline-3-carboxylic acid (8c)

Copper (II) sulfate pentahydrate (19 mg, 0.12 mmol) and sodium ascorbate (30 mg, 0.15 mmol) were added to a stirred solution of azide **4b** (100 mg, 0.10 mmol) and alkyne **6** (44 mg, 0.12 mmol) in dry DMF (2 mL) and stirred at rt for 1 h, according to procedure #1. Purification by silica-gel column chromatography (MeCN/H_2O, 96:4) yielded the title compound **8c** fractions (R_f = 0.21, MeCN/H_2O, 95:5) as an orange solid (80 mg, 61%, mp: 164-167 °C).

$[\alpha]_D^{26.9\,°C}$ -42.8 (c 0.14, MeOH). **IR (neat):** ν_{max} 2959, 2825, 2001, 1740, 1626, 1457, 1369, 1214, 1131, 1042 cm^{-1}. **^{1}H NMR (CD_3OD):** δ 8.86 (s, 1H), 7.89 (s, 1H), 7.72 (d, *J* = 13.3 Hz, 1H), 7.50 (d, *J* = 7.0 Hz, 1H), 5.41-5.39 (m, 2H), 5.24-5.19 (m, 2H-α), 5.14 (dd, *J* = 10.2 Hz, 3.5 Hz, 1H), 5.08 (d, *J* = 7.8 Hz, 1H), 5.05-5.02 (m, 1H), 4.93 (dd, *J* = 10.8 Hz, 3.9 Hz, 1H), 4.89-4.87 (m, 1H), 4.70 (d, *J* = 7.8 Hz, 1H-β), 4.67 (d, *J* = 11.5 Hz, 1H), 4.59 (t, *J* = 4.2 Hz, 2H), 4.50 (d, *J* = 8.0 Hz, 1H-β), 4.40 (dd, *J* = 11.4 Hz, 6.8 Hz, 1H), 4.30 (dd, *J* = 11.4 Hz, 6.9 Hz, 1H), 4.18-4.09 (m, 3H), 3.99-3.92 (m, 2H), 3.83 (t, *J* = 9.5 Hz, 1H), 3.78 (s, 2H), 3.73 (app. as bs, 1H), 3.71-3.67 (m, 1H), 3.40 (app. as singlet, 4H), 2.76 (app. as singlet, 4H), 2.14 (s, 3H), 2.13 (s, 3H), 2.12 (s, 3H), 2.06 (s, 3H), 2.04 (s, 3H), 1.98 (s, 3H), 1.97 (s, 3H), 1.93 (s, 3H), 1.93 (s, 3H), 1.40 (d, *J* = 6.2 Hz, 2H), 1.22 (app. as bs, 2H), 1.15 (d, *J* = 6.5 Hz, 3H). **^{13}C NMR (CD_3OD):** δ 178.12, 172.42, 172.39, 172.20, 172.10 (2 x C), 171.53, 171.38, 171.26, 170.96, 169.29, 154.98 (d, *J* = 250.3 Hz), 149.18, 147.01 (d, *J* = 9.9 Hz), 144.25, 140.68, 126.25, 120.36 (d, *J* = 8.0 Hz), 112.34 (d, *J* = 23.9 Hz), 108.45, 107.08, 101.80, 101.68, 96.86, 75.84, 75.37, 75.09, 74.49, 72.69, 72.35, 72.17, 70.51, 69.48, 69.37, 68.83, 68.60, 66.11, 65.44, 63.14, 62.38, 53.62, 53.46, 50.64, 50.60, 36.96, 21.38, 20.96, 20.91, 20.85, 20.76, 20.65, 20.64, 20.49 20.48, 16.34, 8.57. **^{19}F NMR (CD_3OD):** δ -122.63 (s, 1F). **HRMS (ESI):** Calculated for $C_{58}H_{74}FN_6O_{27}$ [M + H$^+$] 1305.4580, found 1305.4580.

2,3,4-*O*-acetyl-α-L-fucopyranosyl-(1→3)-[2,3,4,6-*O*-acetyl-β-D-galactopyranosyl-(1→4)]-2,6-*O*-acetyl-β-D-glucopyranosyl-5-amino-1-cyclopropyl-6,8-difluoro-7-(4-((1-methyl-1*H*-1,2,3-triazol-4-yl)methyl)-3,5-dimethylpiperazin-1-yl)-4-oxo-1,4-dihydroquinoline-3-carboxylic acid (8d)

Copper (II) sulfate pentahydrate (16 mg, 0.10 mmol) and sodium ascorbate (24 mg, 0.12 mmol) were added to a stirred solution of azide **4b** (100 mg, 0.10 mmol) and alkyne **7** (43 mg, 0.10 mmol) in dry DMF (3 mL) and stirred at rt for 1 h, according to procedure #1. Purification by silica-gel column chromatography (DCM/MeOH, 99:1 – 9:1 – MeCN/H_2O, 9:1) yielded the title compound **8d** (R_f = 0.26, DCM/MeOH, 99:1) as an orange powder (55 mg, 40%, mp: 168-171 °C).

$[\alpha]_D^{26.8\ °C}$ -116 (c 0.12, MeOH). **IR (neat):** ν_{max} 3477, 2967, 2880, 2021, 1741, 1628, 1436, 1369, 1215, 1131, 1043 cm^{-1}. **^{1}H NMR (CD_3OD):** δ 8.51 (s, 1H), 7.92 (s, 1H), 5.40 (dd, *J* = 5.9 Hz, 3.2 Hz, 2H), 5.24-5.20 (m, 2H- α), 5.15-5.02 (m, 3H), 4.95-4.89 (m, 2H), 4.68 (d, *J* = 7.9 Hz, 1H-β), 4.66-4.59 (m, 3H), 4.49 (d, *J* = 7.9 Hz, 1H-β), 4.40 (dd, *J* = 11.3 Hz, 6.8 Hz, 1H), 4.31 (dd, *J* = 11.3 Hz, 7.1 Hz, 1H), 4.20 (s, 2H), 4.15-4.10 (m, 3H), 3.95 (app as triplet, *J* = 9.3 Hz, 3H), 3.82 (t, *J* = 9.5 Hz, 1H), 3.69-3.66 (m, 1H), 3.37 (d, *J* = 11.1 Hz, 2H), 3.10 (t, *J* = 9.7 Hz, 2H), 2.70 (app. as singlet, 2H), 2.15 (s, 3H), 2.14 (s, 3H), 2.10 (3H), 2.05 (s, 3H), 2.04 (s, 3H), 1.98 (s, 3H), 1.94 (s, 3H), 1.93 (s, 3H), 1.93 (s, 3H), 1.31 (d, *J* = 4.1 Hz, 6H), 1.18 (app. as doublet, *J* = 4.1 Hz, 5H), 1.09 (app. as singlet, 2H). **^{13}C NMR (CD_3OD):** δ 181.32, 172.42, 172.40, 172.17, 172.10 (2 x C), 171.53, 171.39, 171.26, 170.97, 169.03, 151.07, 142.00, 141.03 (d, *J* = 239.2 Hz), 137.87 (d, *J* = 13.3 Hz), 137.44 (d, *J* = 236.6 Hz), 135.12 (d, *J* = 10.4 Hz), 135.01 (d, *J* = 11.2 Hz), 129.40, 126.48, 10669, 101.78, 101.70, 96.85, 75.88, 75.37, 75.14, 74.49, 72.70, 72.36, 72.16, 70.52, 69.46, 69.41, 68.79, 68.59, 65.47, 63.17, 62.36, 58.84, 54.86, 54.82, 51.37, 42.26, 41.98, 41.83, 21.35, 20.96, 20.88, 20.81, 20.76, 20.64 (2 x C), 20.4 (2 x C), 17.60, 17.58, 16.35, 9.51, 9.43. **^{19}F NMR (CD_3OD):** δ -145.49 (s, 1F), -149.66 (s, 1F). **HRMS (ESI):** Calculated for $C_{60}H_{78}F_2N_7O_{27}$ [M + H^+] 1366.4908, found 1366.4918.

α-L-fucopyranosyl-(1→2)-β-D-galactopyranosyl-(1→4)-β-D-glucopyranosyl-1-cyclopropyl-7-(4-((1-methyl-1*H*-1,2,3-triazol-4-yl)methyl)piperazin-1-yl)-6-fluoro-4-oxo-1,4-dihydroquinoline-3-carboxylic acid (9a)

Compound **8a** (96 mg, 0.074 mmol) and MeONa (10 mg, 0.18 mmol) were dissolved in MeOH (1 mL), and the reaction was stirred at rt for 1 h according to procedure #2. Purification by silica-gel column chromatography (MeCN/H_2O, 8:2) yielded the title compound **9a** (R_f = 0.30, MeCN/H_2O, 7:3) as an orange solid (32 mg, 47%, mp: 183-186 °C).

$[\alpha]_D^{29.8\ °C}$ -50 (c 0.16, H_2O). **IR (neat):** ν_{max} 3308, 2885, 1965, 1707, 1625, 1456, 1112, 1027 cm^{-1}. **^{1}H NMR (D_2O):** δ 8.39 (s, 1H), 8.19 (s, 1H), 7.22 (s, 1H), 5.28 (d, J = 2.2 Hz, 1H-α), 4.72 (d, J = 4.5 Hz, 2H), 4.46 (d, J = 7.9 Hz, 1H-β), 4.43 (d, J = 8.1 Hz, 1H-β), 4.34-4.31 (m, 1H), 4.20 (d, J = 6.5 Hz, 1H), 4.17-4.12 (m, 1H), 4.02 (app. as singlet, 2H), 3.94 (d, J = 11.1 Hz, 1H), 3.87-3,71 (m, 11H), 3.66-3.62 (m, 3H), 3.56 (t, J = 9.1 Hz, 2H), 3.45-3.41 (m, 1H), 3.35-3.28 (m, 4H), 2.89 (bs, 2H), 1.35 (bs, 2H), 1.19 (d, J = 5.9 Hz, 4H), 1.04 (bs, 2H). **^{13}C NMR (D_2O):** δ 175.20, 169.30, 151.57, 147.57, 144.50, 144.43, 140.34, 138.50, 126.75, 118.46, 110.47 (d, J = 18.9 Hz), 107.93 (d, J = 16.1 Hz), 105.78, 102.37, 100.31, 99.39, 76.37, 75.98, 75.30, 75.19, 74.16, 73.51, 72.72, 71.64, 69.59, 69.04, 68.18, 68.04, 66.87, 61.03, 60.16, 50.88, 50.35, 48.25, 35.65, 15.30, 7.41. The C-F carbon is proved by ^{19}F NMR data. **^{19}F NMR (D_2O):** δ -120.85 (s, 1F).

α-L-fucopyranosyl-(1→2)-β-D-galactopyranosyl-(1→4)-β-D-glucopyranosyl-5-amino-1-cyclopropyl-6,8-difluoro-7-(4-((1-methyl-1*H*-1,2,3-triazol-4-yl)methyl)-3,5-dimethylpiperazin-1-yl)-4-oxo-1,4-dihydroquinoline-3-carboxylic acid (9b)

Compound **8b** (90 mg, 0.07 mmol) and MeONa (10 mg, 0.10 mmol) were dissolved in MeOH (1 mL), and the reaction was stirred at rt for 1 h according to procedure #2. Purification by silica-gel column chromatography (MeCN/H_2O, 8:2 – 7:3) yielded the title compound **9b** (R_f = 0.30, MeCN/H_2O, 7:3) as a light-yellow solid (42 mg, 65%, mp: 212-215 °C).

$[\alpha]_D^{27.7\ °C}$ -60.0 (c 0.10, H_2O). **IR (neat):** ν_{max} 3266, 2882, 2029, 1706, 1629, 1438, 1289, 1037 cm^{-1}.

^{1}H NMR (D_2O): δ 8.26 (app. as singlet, 2H), 5.24 (d, J = 3.3 Hz, 1H-α), 4.74-4.72 (m, 2H), 4.42 (d, J = 7.7 Hz, 1H-β), 4.36 (s, 1H), 4.31 (d, J = 7.4 Hz, 2H-β), 4.15-4.11 (m, 2H), 3.88-3.85 (m, 2H), 3.80-3.71 (m, 7H), 3.69-3.65 (m, 2H), 3.62 (d, J = 7.7 Hz, 1H), 3.59-3.53 (m, 3H), 3.40-3.25 (m, 4H), 3.15 (app. as bs, 2H), 2.84-2.78 (m, 2H), 1.37 (s, 6H), 1.17 (d, J = 6.5 Hz, 3H), 1.06 (app. as bs, 2H), 0.90 (app. as bs, 2H). **^{13}C NMR (D_2O):** δ 178.64, 169.54, 149.18, 138.57, 135.20, 127.48, 127.05, 102.08, 100.43, 99.53, 76.64, 76.26, 75.31, 75.26, 74.16, 73.41, 72.83, 71.63, 69.60, 68.96, 68.23, 67.82, 66.92, 61.00, 60.32, 56.31, 54.11, 50.29, 40.03, 23.21, 15.63, 15.29, 8.22. Three quaternary carbons in sparfloxacin are not visible due to low concentration and the presence of the two C-F carbons are proved by ^{19}F NMR data. **^{19}F NMR (D_2O):** δ -143.80 (bs, 1F), -148.31 (bs, 1F). **HRMS (ESI):** Calculated for $C_{42}H_{60}F_2N_7O_{18}$ [M + H^+] 988.3957, found 988.3959.

α-L-fucopyranosyl-(1→3)-[β-D-galactopyranosyl-(1→4)]-β-D-glucopyranosyl-1-cyclopropyl-7-(4-((1-methyl-1*H*-1,2,3-triazol-4-yl)methyl)piperazin-1-yl)-6-fluoro-4-oxo-1,4-dihydroquinoline-3-carboxylic acid (9c)

Compound **8c** (71 mg, 0.05 mmol) and MeONa (7 mg, 0.13 mmol) were dissolved in dry MeOH (1 mL), and the reaction was stirred at rt for 1 h according to procedure #2. Purification by silica-gel column chromatography (MeCN/H_2O, 9:1 – 8:2 – 7:3) yielded the title compound **9c** (R_f = 0.2, MeCN/H_2O, 7:3) as a slightly yellow powder (27 mg, 54%, mp: 212-215 °C).

$[\alpha]_D^{26.9\ °C}$ -61.5 (c 0.13, H_2O). **IR (neat):** ν_{max} 3378, 2919, 2001, 1707, 1624, 1457, 1259, 1111, 1055, 1032 cm^{-1}. **^{1}H NMR (D_2O):** δ 8.52 (s, 1H), 8.18 (s, 1H), 7.44 (s, 1H), 7.40 (s, 1H), 5.40 (d, J = 3.7 Hz, 1H-α), 4.80 (determined by ^{1}H-^{13}C HSQC) 4.72 (app. as bs, 2H), 4.45 (d, J = 7.8 Hz, 1H-β), 4.38 (d, J = 7.6 Hz, 1H-β), 4.33 (m, 1H), 4.12 (m, 1H), 4.03 (app. as singlet, 2H), 3.97-3.95 (m, 2H), 3.92 (d, J = 3.2 Hz, 1H), 3.86 (d, J = 2.9 Hz, 1H), 3.83-3.66 (m, 8H), 3.63-3.60 (m, 2H), 3.55-3.44 (m, 6H), 3.38 (app. as bs, 2H), 2.93 (app. as bs, 2H), 1.37 (app. as bs, 2H), 1.15 (d, J = 6.6 Hz, 3H), 1.10 (app. as bs, 2H). **^{13}C NMR (D_2O):** δ 175.56, 169.98, 151.86, 147.75, 144.52, 140.38, 138.72, 126.68, 119.34, 110.73, 106.26, 102.31, 101.77, 98.36, 76.88, 75.33, 74.91, 74.31, 72.72, 72.42, 71.90, 71.09, 69.28, 68.28, 68.16, 68.03, 66.46, 61.43, 59.73, 50.89, 50.35, 48.45, 15.21, 7.40. Tertiary aromatic carbons in ciprofloxacin are not visible due to low concentration. The presence of the C-F was proved by ^{19}F NMR spectra. **^{19}F NMR (D_2O):** δ -121.41 (s, 1F). **HRMS (ESI):** Calculated for $C_{40}H_{56}FN_6O_{18}$ [M + H^+] 927.3630, found 927.3622.

α-L-fucopyranosyl-(1→3)-[β-D-galactopyranosyl-(1→4)]-β-D-glucopyranosyl-5-amino-1-cyclopropyl-6,8-difluoro-7-(4-((1-methyl-1*H*-1,2,3-triazol-4-yl)methyl)-3,5-dimethylpiperazin-1-yl)-4-oxo-1,4-dihydroquinoline-3-carboxylic acid (9d)

Compound **8d** (89 mg, 0.07 mmol) and MeONa (7 mg, 0.13 mmol) were dissolved in dry MeOH (1 mL), and the reaction was stirred at rt for 30 min, according to procedure #2. Purification by silica-gel column chromatography (MeCN/H_2O, 9:1, 8:2, 7:3) yielded the title compound **9d** (R_f = 0.2, MeCN/H_2O, 7:3) as a slightly yellow powder (40 mg, 63%, mp: 215-218 °C).

$[\alpha]_D^{27.9\ °C}$ -33.3 (c 0.12, H_2O). **IR (neat):** ν_{max} 3409, 2924, 1961, 1630, 1437, 1068, 1036 cm^{-1}. **^{1}H NMR ($(CD_3)_2SO$):** δ 8.44 (s, 1H), 8.12 (s, 1H), 7.16 (bs, 1H), 5.37 (s, 1H), 5.14 (d, J = 3.2 Hz, 1H-α), 5.00 (s, 1H), 4.71 (bs, 1H), 4.60 (d, J = 6.6 Hz, 1H), 4.57-4.49 (m, 3H), 4.34 (bs, 1H), 4.30 (d, J = 7.8 Hz, 1H-β), 4.25 (d, J = 6.3 Hz, 1H-β), 4.21-4.16 (m, 2H), 4.09-4.06 (m, 2H), 3.96 (bs, 1H), 3.88-3.86 (m, 1H), 3.71-3.63 (m, 5H), 3.25-3.24 (m, 7H), 2.96 (t, J = 10.5 Hz, 2H), 1.17 (d, J = 3.6 Hz, 6H), 1.07 (d, J = 6.2 Hz, 2H), 1.02 (s, 2H), 0.98 (d, J = 6.4 Hz, 3H). **^{13}C NMR ($(CD_3)_2SO$):** δ 179.61, 165.84, 150.02, 140.86, 139.14 (d, J = 238.1 Hz), 136.43 (d, J = 9.9 Hz), 136.32 (d, J = 10.4 Hz), 135.42 (d, J = 235.9 Hz), 133.59 (d, J = 14.2 Hz), 128.11 (d, J = 5.5 Hz), 124.92, 105.25, 102.38, 102.38, 99.28, 78.73, 75.98, 74.87, 74.70, 73.56, 73.39, 71.95, 71.06, 69.55, 68.90, 67.59, 67.53, 65.68, 60.03, 59.43, 57.80,

52.91, 49.59, 40.89, 40.66, 17.43, 16.64, 8.61. **^{19}F NMR ((CD$_3$)$_2$SO):** *δ* -144.83 (s, 1F), -147.91 (s, 1F). **HRMS (ESI):** Calculated for $C_{42}H_{60}F_2N_7O_{18}$ [M + H$^+$] 988.3957, found 988.3959.

Per-*O*-acetylated-β-D-galactopyranosyl-(1→3)-2-deoxy-2-acetamido-β-D-glucopyranosyl-(1→3)-β-D-galactopyranosyl-(1→4)-D-glucopyranosyl (10a)

Lacto-*N*-tetraose (3.0 g, 4.24 mmol) was dissolved in pyridine (10 mL) and acetic anhydride (40 mL) at 0 °C and then stirred while equilibrating to rt for 18 h. The mixture was dissolved in EtOAc (20 mL) and was washed with HCl (2 M, 3 x 30 mL) before being dried over $MgSO_4$. Filtration and concentration gave a crude product that was purified by silica-gel column chromatography (EtOAc). Isolation of the relevant fractions (R_f = 0.3, EtOAc) yielded the title compound **10a** as a white foam (4.83 mg, 91%, mp: 118-121 °C, ratio α/β is 43:57, according to ^{1}H NMR analysis).

$[\alpha]_D^{27.1\,°C}$ 20.4 (c 0.49, MeOH). **IR (neat):** ν_{max} 1736, 1385, 1209, 1035 cm^{-1}. **^{1}H NMR (CDCl$_3$):** *δ* 6.24 (d, *J* = 3.6 Hz, 0.43H-α), 5.82-5.73 (m, 1H), 5.66 (d, *J* = 8.1 Hz, 0.57H-β), 5.,42 (t, *J* = 9.8 Hz, 0.50H), 5.32 (m, 2H), 5.21 (t, *J* = 8.8 Hz, 0.50H), 5.12 (dd, *J* = 4.9 Hz, 8.1 Hz, 1H-β), 5.05-4.91 (m, 5H), 4.56 (t, *J* = 9.8 Hz, 1H), 4.44 (dd, *J* = 7.9 Hz, 1.2 Hz, 1H-β), 4.41-4.28 (m, 1H), 4.34 (dd, *J* = 7.9 Hz, 3.2 Hz, 1H-β), 4.16-4.12 (m, 1H), 4.10-4.02 (m, 4H), 3.98 (dd, *J* = 12 Hz, 3.7 Hz, 1H), 3.83 (t, *J* = 6.5 Hz, 1H), 3.80-3.72 (m, 4H), 3.60 (d, *J* = 10.1 Hz, 1H), 2.85-2.78 (m, 1H), 2.21 (s, 1H), 2.17 (s, 1H), 2.13 (s, 3H), 2.12 (s, 1H), 2.11 (s, 3H), 2.10 (s, 2H), 2.08 (s, 13H), 2.06 (s, 2H), 2.05 (s, 3H), 2.03-2.02 (m, 4H), 2.01 (s, 3H), 1.99-1.98 (m, 4H), 1.95 (s, 3H). **^{13}C NMR (CDCl$_3$):** *δ* 175.32, 171.60, 170.96, 170.72, 170.70, 170.63, 170.60, 170.50, 170.33, 170.27, 170.25, 170.06, 169.86, 169.84, 169.64, 169.59, 169.52, 169.47, 169.15, 169.07, 169.03, 101.01, 100.85, 100.64, 98.85, 98.82, 91.69, 89.11, 76.35, 76.06, 75.27, 75.10, 73.68, 72.50, 71.91, 71.42, 71.36, 71.20, 71.05, 70.94, 70.64, 70.51, 69.64, 69.51, 69.47, 69.28, 68.95, 66.97, 61.95, 61.68, 61.60, 61.46, 61.05, 60.53, 59.00, 23.77, 22.30, 21.07, 21.03, 20.96, 20.93, 20.91, 20.83, 20.78, 20.71, 20.66, 20.62, 14.31. **HRMS (ESI):** Calculated for $C_{52}H_{71}NNaO_{34}^+$ [M + Na$^+$] 1276.3750, found 1276.3758.

Per-*O*-acetylated-β-D-galactopyranosyl-(1→4)-2-deoxy-2-acetamido-β-D-glucopyranosyl-(1→3)-β-D-galactopyranosyl-(1→4)-D-glucopyranosyl (10b)

Lacto-*N*-neotetraose (3.0 g, 4.2 mmol) was dissolved in pyridine (10 mL) and acetic anhydride (40 mL) at 0 °C and then stirred while equilibrating to rt for 36 h. The mixture was dissolved in EtOAc (20 mL) and was washed with HCl (2 M, 3 x 30 mL) before being dried over $MgSO_4$. Filtration and concentration gave a crude product that was purified by silica-gel column chromatography (EtOAc). Isolation of the

relevant fractions (R_f = 0.26, EtOAc) yielded the title compound **10b** as a white foam (4.26 g, 80%, mp: 118-121 °C, the ratio α/β is 42:58, according to ^{1}H NMR analysis).

$[\alpha]_D^{26.2°C}$ 21.1 (c 0.19, MeOH). **IR (neat):** ν_{max} 2118, 1736, 1366, 1208, 1036 cm^{-1}. **^{1}H NMR (CDCl$_3$):** *δ* 6.24 (d, *J* = 3.5 Hz, 0.42H-α), 5.65 (d, *J* = 8.2 Hz, 0.58H-β), 5.44-5.29 (m, 3H), 5.23-5.15 (m, 2H), 5.10-4.95 (m, 4H), 4.78 (d, *J* = 11.9 Hz, 1H), 4.65 (d, *J* = 7.7 Hz, 1H-β), 4.53 (d, *J* = 7.7 Hz, 1H-β), 4.39 (dd, *J* = 11.8 Hz, 6.2 Hz, 1H), 4.32 (d, *J* = 7.7 Hz, 1H-β), 4.15-4.04 (m, 5H), 4.00-3.93 (m, 2H), 3.86 (t, *J* = Hz, 1H), 3.78-3.70 (m, 4H), 3.54-3.48 (m, 2H), 2.14 (s, 6H), 2.11 (s, 6H), 2.10 (s, 3H), 2.08-2.05 (m, 12H), 2.03 (s, 3H), 2.01 (s, 3H), 2.01 (s, 3H), 1.95 (s, 3H), 1.89 (s, 3H). **^{13}C NMR (CDCl$_3$):** *δ* 170.76, 170.73, 170.64, 170.62, 170.57, 170.48, 170.24, 170.19, 170.06, 169.94, 169.92, 169.81, 169.76, 169.65, 169.23, 169.09, 169.07, 168.99, 168.98, 101.17, 100.94, 100.59, 100.40, 100.36, 91.65, 89.07, 75.95, 75.87, 75.82, 75.29, 75.15, 73.67, 72.73, 72.46, 72.07, 71.24, 71.20, 71.11, 70.97, 70.89, 70.79, 70.52, 69.47, 69.27, 68.99, 66.73, 61.95, 61.69, 61.63, 60.87, 60.50, 60.40, 55.06, 23.22, 21.14, 21.05, 20.97, 20.95, 20.90, 20.87, 20.84, 20.78, 20.74, 20.72, 20.68, 20.65, 20.61, 14.28. **HRMS (ESI):** Calculated for $C_{52}H_{72}NO_{34}$ [M + H$^+$] 1254.3930, found 1254.3940.

2,3,4,6-*O*-acetyl-β-D-galactopyranosyl-(1→3)-2-deoxy-2-acetamido-4,6-*O*-acetyl-β-D-glucopyranosyl-(1→3)-2,4,6-*O*-acetyl-β-D-galactopyranosyl-(1→4)-2,3,6-*O*-acetyl-D-glucopyranosyl-(4-(2-azidoethyl)phenoxy) (11a)

Acetated Lacto-*N*-Tetraose **10a** (2.0 g, 1.6 mmol), 4-(2-azidoethyl)phenol (388 mg, 2.4 mmol) and NEt_3 (60 drops) were dissolved in dry DCM (30 mL). Under inert atm., $BF_3.OEt_2$ (1.55 mL, 670 mmol) was added. The reaction mixture was stirred at rt for 1 h, before being quenched with $NaHCO_3$ aq. sat. (20 mL), extracted with DCM (3 x 20 mL) and dried over $MgSO_4$. Filtration and concentration gave a crude product that was purified by silica-gel column chromatography (pet. ether/EtOAc, 3:7). Isolation of the relevant fractions (R_f = 0.50, EtOAc) yielded the title compound **11a** as a white foam (*β*-selective, 198 mg, 10%, mp: 129-132 °C, ratio α/β is 1:>99 according to ^{1}H NMR analysis).

$[\alpha]_D^{27.0\,°C}$ 2.12 (c 0.94, MeOH). **IR (neat):** ν_{max} 2942, 2101, 1741, 1367, 1214, 1040 cm^{-1}. **^{1}H NMR (CDCl$_3$):** *δ* 7.03 (d, *J* = 8.6 Hz, 2H), 6.83 (d, *J* = 8.1 Hz, 2H), 6.06 (d, *J* = 6.0 Hz, 1H), 5.28 (d, *J* = 3.2 Hz, 1H), 5.24 (d, *J* = 3.0 Hz, 1H), 5.18 (t, *J* = 8.8 Hz, 1H), 5.08 (d, *J* = 7.8 Hz, 1H-β), 5.06 (dd, *J* = 8.0 Hz, 2.3 Hz, 1H), 4.97-4.80 (m, 5H-β), 4.51 (t, *J* = 9.9 Hz, 1H), 4.41-4.26 (m, 2H-β), 4.31 (d, *J* = 7.7 Hz, 1H-β), 4.28 (d, *J* = 10.6 Hz, 1H), 4.10-3.90 (m, 6H), 3.79-3.69 (m, 5H), 3.54 (dt, *J* = 9.7 Hz, 3.0 Hz, 1H), 3.38 (t, *J* = 7.1 Hz, 2H), 2.75 (t, *J* = 6.9 Hz, 3H), 2.06 (s, 3H), 2.02 (s, 3H), 2.02 (s, 3H), 2.01 (s, 3H), 2.01 (s, 3H), 2.01 (s, 3H), 2.01 (s, 3H), 2.01 (s, 3H), 1.97 (s, 3H), 1.97 (s, 3H), 1.97 (s, 3H), 1.96 (s, 3H), 1.91 (s, 3H), 1.88 (s, 3H). **^{13}C NMR (CDCl$_3$):** *δ* 171.30, 170.68, 170.48, 170.33, 170.26, 170.09, 169.75, 169.45 (2 x C), 169.44, 169.24, 169.17 (2 x C), 155.63, 132.87, 129.76, 116.99, 100.66 (2 x C),

98.84, 98.74, 76.37, 75.85, 75.52, 72.75, 72.39, 71.72, 71.31, 71.26, 71.03, 70.69, 70.45, 69.37, 69.14, 68.77, 66.78, 62.03, 61.50, 61.36, 60.91, 58.69, 52.38, 34.38, 23.54, 20.82, 20.75, 20.68 (2 x C), 20.66 (2 x C), 20.59 (2 x C), 20.57 (2 x C), 20.54, 20.47. **HRMS (ESI):** Calculated for $C_{58}H_{77}N_4O_{33}$ [M + H^+] 1357.4465, found 1357.4472.

2,3,4,6-*O*-acetyl-β-D-galactopyranosyl-(1→4)-2-deoxy-2-acetamido-3,6-*O*-acetyl-β-D-glucopyranosyl-(1→3)-2,4,6-*O*-acetyl-β-D-galactopyranosyl-(1→4)-2,3,6-*O*-acetyl-β-D-glucopyranosyl-(4-(2-azidoethyl)phenoxy) (11b)

Acetated Lacto-*N*-neoTetraose **10b** (1.2 g, 0.96 mmol), 4-(2-azidoethyl)phenol (233 mg, 1.43 mmol) and NEt_3 (36 drops) were dissolved in dry DCM (15 mL). Under inert atm., $BF_3.OEt_2$ (0.82 mL, 670 mmol) was added. The reaction mixture was stirred at rt for 1 h 20, before being quenched with $NaHCO_3$ aq. sat. (10 mL), extracted with DCM (3 x 15 mL) and dried over $MgSO_4$. Filtration and concentration gave a crude product that was purified by silica-gel column chromatography (pet. ether/EtOAc, 1:9). Isolation of the relevant fractions (R_f = 0.23, EtOAc) yielded the title compound **11b** as a white foam (*β*-selective, 350 mg, 27%, mp: 136-139 °C, ratio α/β is 1:>99 according to ^{1}H NMR analysis).

$[\alpha]_D^{26.2\,°C}$ 5.7 (c 0.35, MeOH). **IR (neat):** ν_{max} 2101, 1956, 1741, 1368, 1217, 1045 cm^{-1}. **^{1}H NMR ($CDCl_3$):** *δ* 7.09 (d, *J* = 8.5 Hz, 2H), 6.88 (d, *J* = 8.5 Hz, 2H), 5.49 (d, *J* = 8.4 Hz, 1H), 5.31 (d, *J* = 2.8 Hz, 1H), 5.28 (d, *J* = 3.1 Hz, 1H), 5.23-5.04 (m, 4H), 4.99-4.92 (m, 3H-β), 4.73 (d, *J* = 10.1 Hz, 1H), 4.66 (d, *J* = 7.7 Hz, 1H-β), 4.51 (d, *J* = 8.1 Hz, 1H-β), 4.42 (d, *J* = 10.4 Hz, 1H), 4.34 (d, *J* = 8.1 Hz, 1H-β), 4.13-4.01 (m, 5H), 3.92 (dd, *J* = 12.1 Hz, 2.6 Hz, 1H), 3.86-3.69 (m, 6H), 3.52-3.48 (m, 2H), 3.43 (t, *J* = 7.0 Hz, 2H), 2.79 (t, *J* = 7.3 Hz, 2H), 2.10 (s, 3H), 2.10 (s, 3H), 2.08 (s, 3H), 2.07 (s, 3H), 2.05 (s, 3H), 2.04 (s, 3H), 2.02 (s, 3H), 2.01 (s, 3H), 2.00 (s, 9H), 1.92 (s, 3H), 1.86 (s, 3H). **^{13}C NMR ($CDCl_3$):** *δ* 170.66, 170.50, 170.47, 170.40 (2 x C), 170.37, 170.16, 170.10, 169.83 (2 x C), 169.62, 169.16, 168.96, 155.76, 132.96, 129.86, 117.12, 101.11, 100.78, 100.28, 98.90, 75.83, 75.79, 75.74, 72.90, 72.64, 72.60, 71.93, 71.42, 71.17, 70.99, 70.90, 70.73, 69.19, 68.95, 66.67, 62.19, 61.61, 60.81, 60.39, 55.04, 52.52, 34.51, 23.18, 20.90, 20.87, 20.83, 20.81, 20.78, 20.76, 20.72, 20.68 (2 x C), 20.65 (2 x C), 20.54. **HRMS (ESI):** Calculated for $C_{58}H_{77}N_4O_{33}$ [M + H^+] 1357.4465, found 1357.4470.

2,3,4,6-*O*-acetyl-β-D-galactopyranosyl-(1→3)-2-deoxy-2-acetamido-4,6-*O*-acetyl-β-D-glucopyranosyl-(1→3)-2,4,6-*O*-acetyl-β-D-galactopyranosyl-(1→4)-2,3,6-*O*-acetyl-D-glucopyranosyl-1-cyclopropyl-7-(4-((1-methyl-1*H*-1,2,3-triazol-4-yl)methyl)piperazin-1-yl)-6-fluoro-4-oxo-1,4-dihydroquinoline-3-carboxylic acid (12a)

Copper (II) sulfate pentahydrate (9 mg, 0.06 mmol) and sodium ascorbate (11 mg, 0.07 mmol) were added to a stirred solution of azide **11a** (77 mg, 0.06 mmol) and alkyne **6** (20 mg, 0.06 mmol) in dry DMF (1 mL) at rt for 1 h, according to procedure #1. Purification by silica-gel column chromatography (DCM/MeOH, 95:5 – 9:1 – 8:2) yielded the title compound **12a** (R_f = 0.48, DCM/MeOH, 95:5) as an orange powder (20 mg, 21%, mp: 181-184 °C).

$[\alpha]_D^{27.4\,°C}$ 4.34 (c 0.92, Acetone). **IR (neat):** ν_{max} 2933, 1985, 1740, 1366, 1213, 1040 cm^{-1}. **^{1}H NMR ($(CD_3)_2CO$):** δ 15.00 (bs, 1H), 8.71 (s, 1H), 7.92 (d, *J* = 13.5 Hz, 1H), 7.66 (d, *J* = 7.0 Hz, 1H), 7.20 (d, *J* = 8.8 Hz, 1H), 7.11 (d, *J* = 8.8 Hz, 2H), 6.94 (d, *J* = 8.2 Hz, 2H), 5.42 (d, *J* = 3.5 Hz, 1H), 5.31 (s, 1H), 5.30 (d, *J* = 6.4 Hz, 1H-β), 5.22 (t, *J* = 9.4 Hz, 1H), 5.04-4.96 (m, 5H), 4.84-4.75 (m, 3H-β, β), 4.64-4.60 (m, 3H-β), 4.42 (d, *J* = 11.4 Hz, 1H), 4.23-4.14 (m, 5H), 4.11-4.05 (m, 5H), 4.04-3.99 (m, 5H), 3.87-3.82 (m, 1H), 3.71-3.59 (m, 5H), 3.36 (app. as bs, 3H), 3.18 (t, *J* = 7.0 Hz, 2H), 2.62 (app. as bs, 2H), 2.09 (s, 3H), 2.06 (s, 3H), 2.05 (s, 3H), 2.04 (s, 3H), 2.04 (s, 3H), 2.03 (s, 3H), 2.03 (s, 3H), 2.02 (s, 3H), 2.02 (s, 3H), 1.99 (s, 3H), 1.95 (s, 3H), 1.94 (s, 3H), 1.87 (s, 3H), 1.47 (d, *J* = 7.1 Hz, 2H), 1.31 (app. as bs, 2H). **^{13}C NMR ($(CD_3)_2CO$):** δ 177.94, 170.87, 170.81, 170.78, 170,70, 170.61, 170.45, 170.25 (2 x C), 170.10, 170.01, 169.90, 169.82, 169.57, 166.82, 156.85, 154.51 (d, *J* = 251.6 Hz), 148.87, 140.48, 133.17, 130.79, 124.36, 117.54, 112.11 (d, *J* = 24.1 Hz), 108.56, 107.15, 101.83, 99.04, 78.21, 77.41, 77.20, 73.54, 73.46, 72.29, 71.97, 71.44, 71.06, 70.36, 69.83, 69.66, 68.10, 63.17, 62.55, 62.46, 61.95, 56.66, 53.66, 52.97, 51.80, 50.63, 36.56, 36.36, 23.51, 21.09, 20.98, 20.88 (2 x C), 20.81 (2 x C), 20.77 (2 x C), 20.73, 20.64, 20.60, 20.54, 20.48, 8.47. Tertiary carbons from Ciprofloxacin are overlapping or non-visible due to low concentration. **^{19}F NMR ($(CD_3)_2CO$):** δ -122.55 (s, 1F). **HRMS (ESI):** Calculated for $C_{78}H_{97}FN_7O_{36}$ [M + H^+] 1726.5953, found 1726.5948.

2,3,4,6-*O*-acetyl-β-D-galactopyranosyl-(1→3)-2-deoxy-2-acetamido-4,6-*O*-acetyl-β-D-glucopyranosyl-(1→3)-2,4,6-*O*-acetyl-β-D-galactopyranosyl-(1→4)-2,3,6-*O*-acetyl-D-glucopyranosyl-5-amino-1-cyclopropyl-6,8-difluoro-7-(4-((1-methyl-1*H*-1,2,3-triazol-4-yl)methyl)-3,5-dimethylpiperazin-1-yl)-4-oxo-1,4-dihydroquinoline-3-carboxylicacid (12b)

Copper (II) sulfate pentahydrate (9 mg, 0.06 mmol) and sodium ascorbate (11 mg, 0.07 mmol) were added to a stirred solution of azide **11a** (81 mg, 0.06 mmol) and alkyne **7** (25 mg, 0.06 mmol) in dry DMF (1 mL) and resulting reaction mixture was stirred at rt for 1 h according to procedure #1.

Purification by silica-gel column chromatography (DCM/MeOH, 9:1 – 8:2) yielded the title compound **12b** (R_f = 0.43, DCM/MeOH, 95:5) as an orange powder (50 mg, 48%, mp: 171-174 °C).

$[\alpha]_D^{27.5\,°C}$ -4.87 (c 0.41, Acetone). **IR (neat):** ν_{max} 2961, 1943, 1741, 1629, 1436, 1366, 1214, 1040 cm^{-1}. **^{1}H NMR ((CD$_3$)$_2$CO):** δ 14.4 (s, 1H), 8.57 (s, 1H), 7.65 (s, 1H), 7.19 (d, *J* = 9.1 Hz, 1H), 7.08 (d, *J* = 8.7 Hz, 2H), 6.97 (bs, 2H), 6.90 (d, *J* = 8.7 Hz, 2H), 5.43 (d, *J* = 3.3 Hz, 1H), 5.30 (m, 1H), 5.26 (d, *J* = 8.0 Hz, 1H-β), 5.19 (t, *J* = 9.2 Hz, 1H), 5.02-4.98 (m, 3H-β), 4.87 (dd, *J* = 8.2 Hz, 1.4 Hz, 1H), 4.85-4.76 (m, 3H-β), 4.64-4.62 (m, 3H-β), 4.47 (dd, *J* = 11.8 Hz, 1.6 Hz, 1H), 4.22-4.15 (m, 4H), 4.12-4.04 (m, 6H), 4.03-3.99 (m, 4H), 3.87 (t, *J* = 9.1 Hz, 1H), 3.71-3.59 (m, 3H), 3.34 (d, *J* = 9.1 Hz, 2H), 3.18 (t, *J* = 7.0 Hz, 2H), 3.01 (app. as bs, 2H), 2.53 (d, *J* = 11.6 Hz, 2H), 2.09 (s, 3H), 2.08 (s, 3H), 2.05 (s, 3H), 2.04 (s, 3H), 2.03 (s, 3H), 2.03 (s, 3H), 2.02 (s, 3H), 2.01 (s, 3H), 1.99 (s, 3H), 1.98 (s, 3H), 1.94 (s, 3H), 1.94 (s, 3H), 1.87 (s, 3H), 1.27 (bs, 2H), 1.17 (m, 8H). **^{13}C NMR ((CD$_3$)$_2$CO):** δ 181.23, 170.87, 170.79, 170.78, 170.69, 170.60, 170.46, 170.26, 170.25, 170.12, 170.00, 169.93, 169.90, 169.84, 169.57, 166.32, 156.85, 150.99, 141.89 (d, *J* = 22.5 Hz), 137.43 (d, *J* = 14.7 Hz), 133.15, 130.77, 129.46 (d, *J* = 8.6 Hz), 124.36, 117.55, 107.37, 101.82 (2 x C), 101.78, 99.01, 78.18, 77.41, 77.07, 73.58, 73.36, 72.37, 72.29, 71.96, 71.44, 71.05, 70.36, 69.84, 69.65, 68.09, 63.16, 62.58, 62.46, 61.94, 59.04, 56.66, 53.97, 51.71, 42.02, 41.52, 41.38, 36.33, 23.51, 21.09, 20.97, 20.85, 20.80 (2 x C), 20.77 (2 x C), 20.76 (2 x C), 20.63, 20.60, 20.54, 20.48, 17.90, 9.34, 9.29. The presence of the two C-F carbons is proved by ^{19}F NMR data. **^{19}F NMR ((CD$_3$)$_2$CO):** δ -145.05 (s, 1F), -149.94 (s, 1F). **HRMS (ESI):** Calculated for $C_{80}H_{101}F_2N_8O_{36}$ [M + H$^+$] 1787.6281, found 1787.6276.

2,3,4,6-*O*-acetyl-β-D-galactopyranosyl-(1→4)-2-deoxy-2-acetamido-3,6-*O*-acetyl-β-D-glucopyranosyl-(1→3)-2,4,6-*O*-acetyl-β-D-galactopyranosyl-(1→4)-2,3,6-*O*-acetyl-D-glucopyranosyl-1-cyclopropyl-7-(4-((1-methyl-1*H*-1,2,3-triazol-4-yl)methyl)piperazin-1-yl)-6-fluoro-4-oxo-1,4-dihydroquinoline-3-carboxylic acid (12c)

Copper (II) sulfate pentahydrate (23 mg, 0.15 mmol) and sodium ascorbate (37 mg, 0.19 mmol) were added to a stirred solution of azide **11b** (197 mg, 0.15 mmol) and alkyne **6** (53 mg, 0.15 mmol) in dry DMF (1 mL) and the resulting reaction mixture was stirred at rt for 1 h, according to procedure #1. Purification by silica-gel column chromatography (MeCN/H_2O, 98:2 – 95:5) yielded the title compound **12c** (R_f = 0.50, MeCN/H_2O, 95:5) as an orange powder (90 mg, 36%, mp: 161-163 °C).

$[\alpha]_D^{26.8\,°C}$ -22.22 (c 0.18, MeOH). **IR (neat):** ν_{max} 2946, 1983, 1739, 1627, 1368, 1214, 1042 cm^{-1}. **^{1}H NMR (CD$_3$OD):** δ 8.81 (s, 1H), 7.95 (d, *J* = 13.1 Hz, 1H), 7.57 (d, *J* = 7.6 Hz, 1H), 7.49 (s, 1H), 6.99 (d, *J* = 8.7 Hz, 2H), 6.83 (d, *J* = 8.7 Hz, 2H), 5.42 (d, *J* = 3.4 Hz, 1H), 5.35 (d, *J* = 3.5 Hz, 1H), 5.17 (dd, *J* = 9.0 Hz, 8.0 Hz, 1H), 5.15-5.10 (m, 2H), 5.06 (d, *J* = 7.9 Hz, 1H-β), 5.00 (dd, *J* = 10.4 Hz, 7.7 Hz, 1H), 4.92-4.88 (m, 2H), 4.72 (d, *J* = 8.0 Hz, 1H-β), 4.69-4.64 (m, 4H-β), 4.47 (d, *J* = 7.9 Hz, 1H-

β), 4.22 (d, J = 10.1 Hz, 1H), 4.12 (app. as s, 3H), 4.09-3.98 (m, 4H), 3.90-3.76 (m, 5H), 3.69-3.60 (m, 4H), 3.57 (dd, 10.4 Hz, 8.3 Hz, 1H), 3.37-3.35 (m, 2H), 3.27-3.25 (m, 2H), 3.17 (t, J = 6.4 Hz, 2H), 2.54-2.44 (m, 4H), 2.15 (s, 3H), 2.12 (s, 3H), 2.09 (s, 3H), 2.08 (s, 3H), 2.06 (s, 3H), 2.05 (s, 3H), 2.04 (s, 3H), 2.04 (s, 3H), 2.04 (s, 3H), 2.02 (s, 3H), 1.96 (s, 3H), 1.92 (s, 3H), 1.85 (s, 3H), 1.42 (d, J = 7.0 Hz, 2H), 1.26 (app. as bs, 2H). **^{13}C NMR (CD_3OD):** δ 178.38, 173.26, 172.48, 172.33, 172.15, 172.03, 171.93 (2 x C), 171.56, 171.51, 171.42, 171.22, 171.17, 170.90, 169.56, 157.18, 155.20 (d, J = 250.9 Hz), 149.46, 147.35 (d, J = 10.5 Hz), 143.65, 140.91, 133.33, 131.05, 126.18, 120.60 (d, J = 8.2 Hz), 118.13, 112.68 (d, J = 22.3 Hz), 108.35, 107.38, 102.40, 102.07, 101.99, 99.62, 78.17, 77.92, 77.47, 74.61, 73.85, 73.76, 73.68, 73.00, 72.46, 72.20, 71.81, 71.71, 71.08, 70.80, 68.61, 63.71, 62.81, 62.62, 62.25, 56.01, 53.33, 52.95, 52.42, 50.75, 50.70, 37.04, 36.53, 22.95, 21.15, 21.13, 21.09, 21.01, 20.83 (2 x C), 20.78, 20.74, 20.60 (2 x C), 20.4 (2 x C), 8.65, 8.55. **^{19}F NMR (CD_3OD):** δ -121.64. **HRMS (ESI):** Calculated for $C_{78}H_{97}FN_7O_{36}$ [M + H$^+$] 1726.5953, found 1726.5950.

2,3,4,6-*O*-acetyl-β-D-galactopyranosyl-(1→4)-2-deoxy-2-acetamido-3,6-*O*-acetyl-β-D-glucopyranosyl-(1→3)-2,4,6-*O*-acetyl-β-D-galactopyranosyl-(1→4)-2,3,6-*O*-acetyl-D-glucopyranosyl-5-amino-1-cyclopropyl-6,8-difluoro-7-(4-((1-methyl-1*H*-1,2,3-triazol-4-yl)methyl)-3,5-dimethylpiperazin-1-yl)-4-oxo-1,4-dihydroquinoline-3-carboxylic acid (12d)

Copper (II) sulfate pentahydrate (12 mg, 0.074 mmol) and sodium ascorbate (18 mg, 0.093 mmol) were added to a stirred solution of azide **11b** (84 mg, 0.06 mmol) and alkyne **7** (27 mg, 0.07 mmol) in dry DMF (1 mL) and the resulting reaction mixture was stirred at rt for 1 h, according to procedure #1. Purification by silica-gel column chromatography (DCM/MeOH, 95:5 – MeCN/H_2O, 9:1) yielded the title compound **12d** (R_f = 0.39, DCM/MeOH, 9:1) as a yellow foam (99 mg, 76%, mp: 163-166 °C).

$[\alpha]_D^{25.6\,°C}$ 13.3 (c 0.15, MeOH). **IR (neat):** ν_{max} 3331, 2876, 2017, 1741, 1629, 1436, 1368, 1214, 1043 cm^{-1}. **^{1}H NMR (CD_3OD):** δ 8.61 (s, 1H), 7.52 (s, 1H), 6.95 (d, J = 8.3 Hz, 2H), 6.78 (d, J = 8.5 Hz, 2H), 5.44 (d, J = 3.1 Hz, 1H), 5.35 (d, J = 3.4 Hz, 1H), 5.18 (dd, J = 10.1 Hz, 9.2 Hz, 1H), 5.12 (dd, J = 10.4 Hz, 3.4 Hz, 1H), 5.06 (t, J = 9.1 Hz, 1H), 5.00 (dd, J = 10.3 Hz, 7.8 Hz, 1H), 4.94 (d, J = 7.8 Hz, 1H-β), 4.92 (dd, J = 10.0 Hz, 8.1 Hz, 1H), 4.74 (d, J = 7.6 Hz, 1H-β), 4.72 (d, J = 7.9 Hz, 1H-β), 4.70-4.63 (m, 3H), 4.51 (d, J = 8.0 Hz, 1H-β), 4.42-4.37 (m, 2H), 4.13 (bs, 1H), 4.12 (app. as bs, 4H), 4.06 (bs, 2H), 4.04-3.97 (m, 4H), 3.91 (dd, J = 10.0 Hz, 3.0 Hz, 1H), 3.81-3.66 (m, 4H), 3.63-3.61 (m, 1H), 3.55 (dd, J = 10.2 Hz, 8.3 Hz, 1H), 3.36-3.34 (m, 2H), 3.18 (t, J = 5.6 Hz, 2H), 3.05 (t, J = 9.7 Hz, 1H), 2.95 (t, J = 10.8 Hz, 1H), 2.32 (bs, 1H), 2.15 (s, 3H), 2.12 (s, 3H), 2.10 (s, 3H), 2.09 (s, 3H), 2.09 (s, 3H), 2.06 (s, 3H), 2.05 (s, 3H), 2.04 (s, 3H), 2.04 (s, 3H), 1.97 (s, 3H), 1.92 (s, 3H), 1.89 (s, 3H), 1.86 (s, 3H), 1.18 (d, J = 6.2 Hz, 3H), 1.17 (bs, 2H), 1.16 (d, J = 6.2 Hz, 3H), 1.13 (bs, 2H). **^{13}C NMR**

(CD_3OD): δ 181.62, 173.26, 172.45, 172.35, 172.06, 172.00, 171.91, 171.89, 171.64, 171.52, 171.41, 171.14, 170.96, 170.89, 169.32, 157.26, 151.66, 141.48 (dd, J = 236.2 Hz, 5.2 Hz), 140.71, 137.91 (dd, J = 238.3 Hz, 5.7 Hz), 137.80 (d, J = 13.3 Hz), 135.40 (d, J = 13.5 Hz), 135.28 (d, J = 13.5 Hz), 133.21, 130.97, 129.45 (dd, J = 5.5 Hz, 5.8 Hz), 126.36, 118.12, 107.02, 106.97, 106.67, 102.27, 102.00, 99.64, 78.24, 77.60, 77.47, 74.25, 73.89, 73.80, 73.77, 73.07, 72.43, 72.17, 71.80, 71.69, 71.09, 70.79, 68.59, 63.77, 62.97, 62.61, 62.25, 58.94, 58.47, 56.10, 54.27, 54.08, 52.07, 42.10, 41.96, 41.59, 36.36, 23.00, 21.18, 21.10, 21.08, 21.04, 20.93, 20.89, 20.82, 20.76 (2 x C), 20.63, 20.59, 20.50 (2 x C), 17.41, 9.56. **^{19}F NMR (CD_3OD):** δ -144.22 (s, 1F), - 149.29 (s, 1F). **HRMS (ESI):** Calculated for $C_{80}H_{101}F_2N_8O_{36}$ [M + H$^+$] 1787.6281, found 1787.6271.

4-(2-azidoethyl)phenol (14)

Sodium azide (1.56 g, 24 mmol) was added to a solution of 4-(2-bromoethyl)phenol **13** (1.00 g, 5 mmol) in DMF (2 mL). The reaction mixture was stirred at 50 °C for 22 h, was then allowed to cool down to rt and quenched with H_2O. The crude was extracted with DCM (3 x 10 mL), and the combined organic layer were washed with H_2O (10 mL), brine (10 mL), dried over $MgSO_4$. Filtration and concentration gave a light orange oil, that was used directly for next step without further purification (660 mg).

IR (neat): vmax 3226, 2091, 1886, 1654 cm^{-1}. **^{1}H NMR ($CDCl_3$):** δ 7.09 (d, J = 8.5 Hz, 2H), 6.78 (d, J = 8.5 Hz, 2H), 4.75 (bs, 1H), 3.45 (t, J = 7.2 Hz, 2H), 2.82 (t, J = 7.2 Hz, 2H). **^{13}C NMR ($CDCl_3$):** δ 154.50, 130.38, 130.09, 115.63, 52.84, 34.63. The spectroscopic data are in accordance with data previously reported in the literature.[5]

^{1}H, ^{13}C and ^{19}F NMR spectra

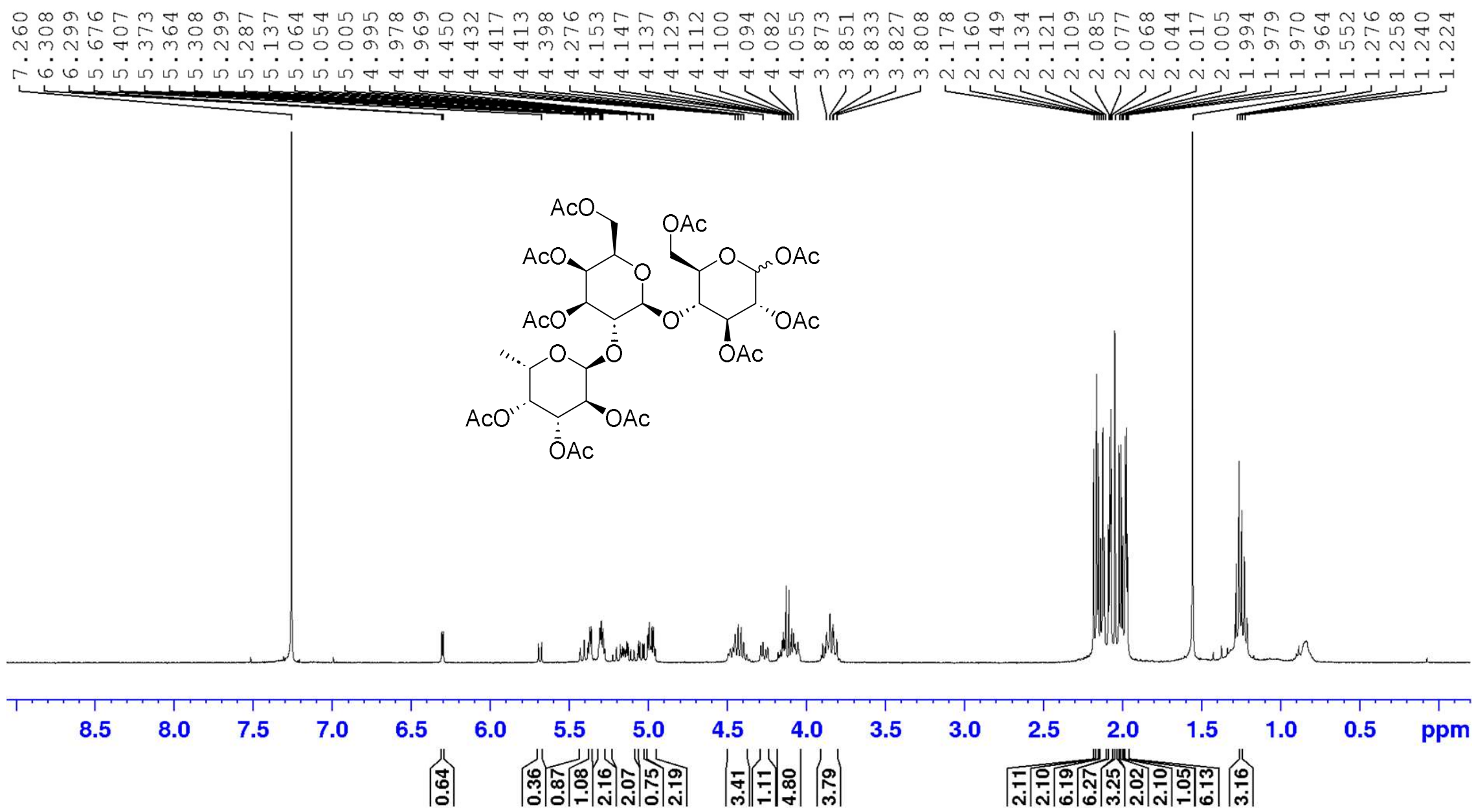

^{1}H NMR (400 MHz, $CDCl_3$) spectrum of compound **1a**.

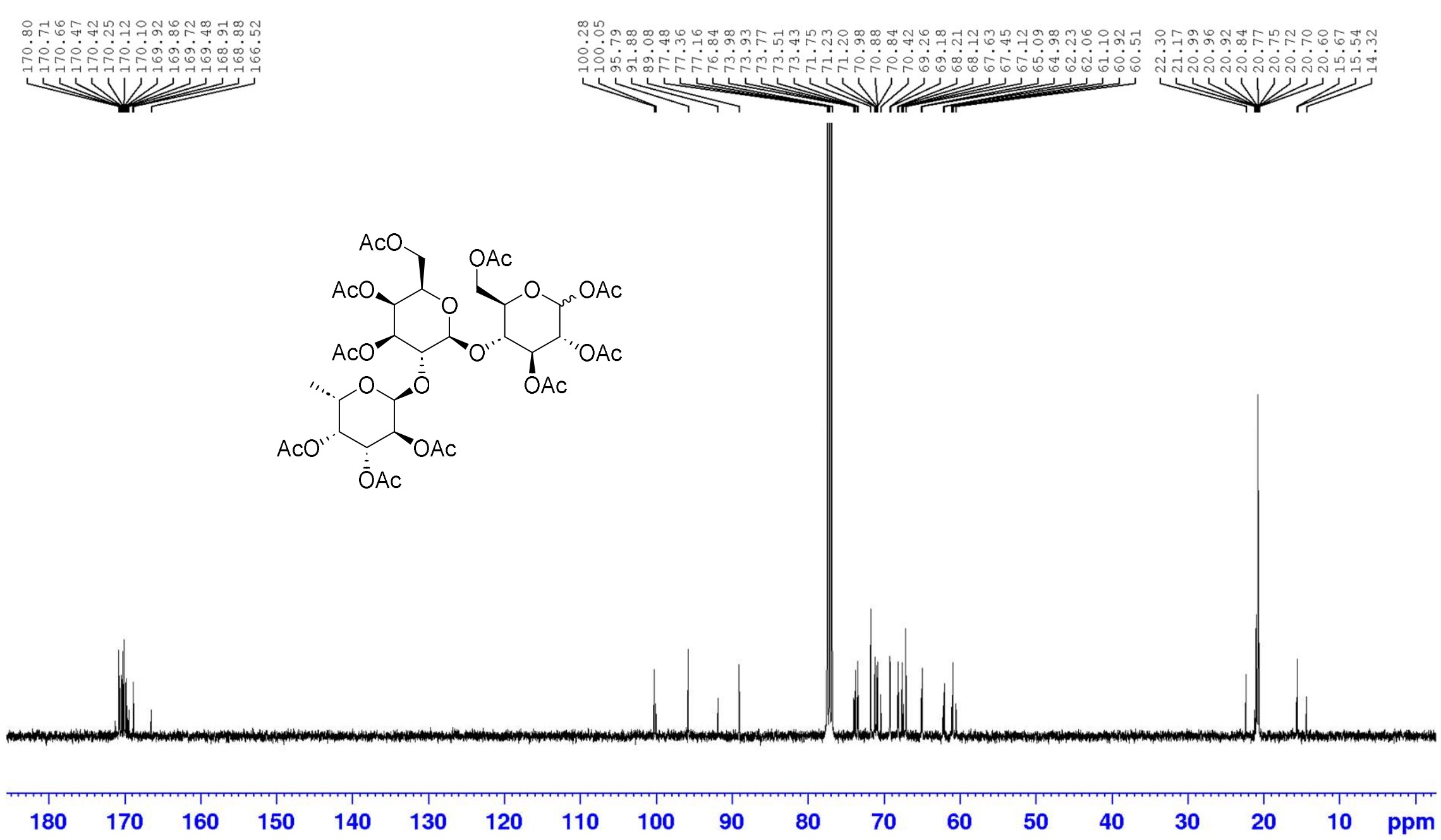

^{13}C NMR (100 MHz, $CDCl_3$) spectrum of compound **1a**.

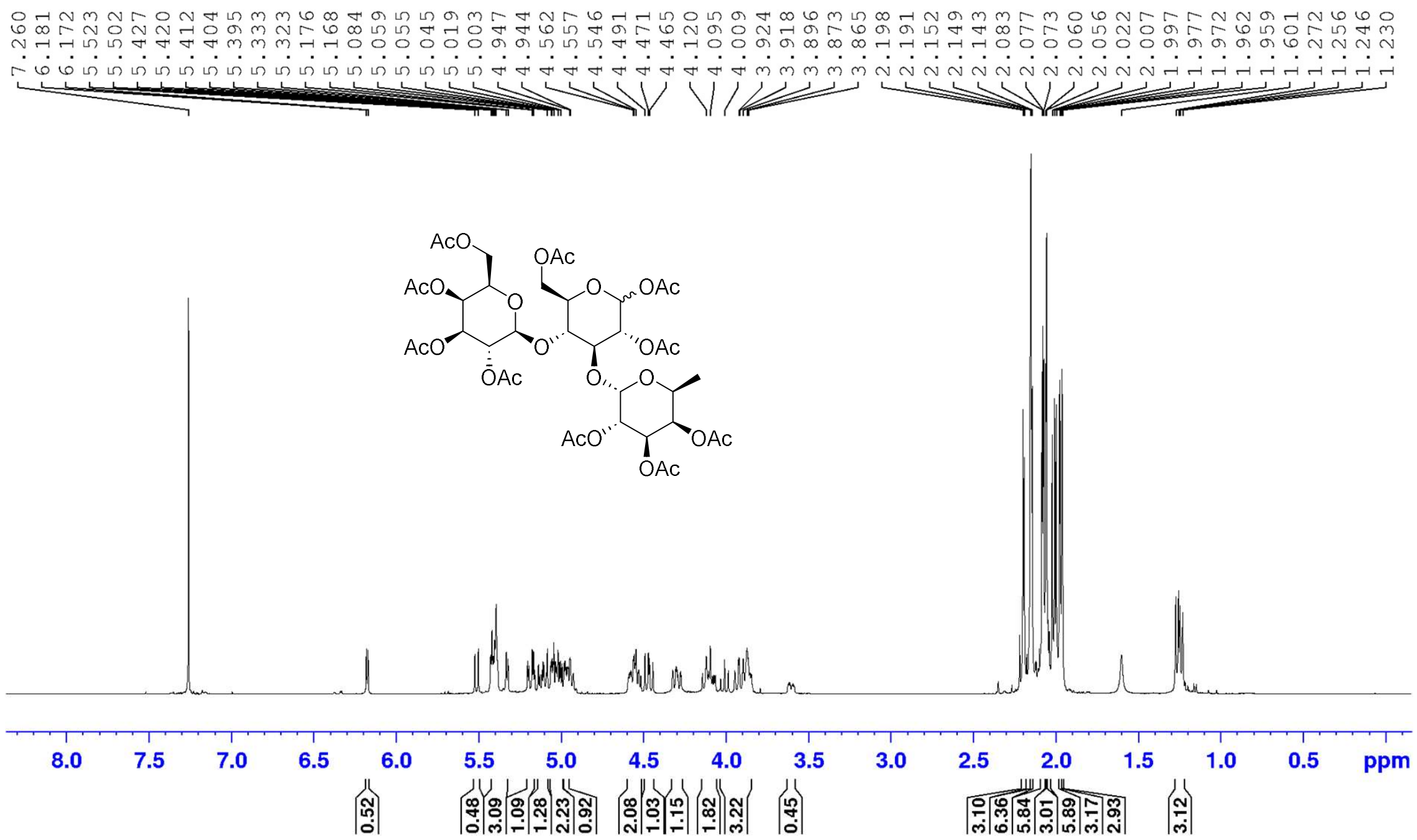

^{1}H NMR (400 MHz, $CDCl_3$) spectrum of compound **1b**.

^{13}C NMR (100 MHz, $CDCl_3$) spectrum of compound **1b.**

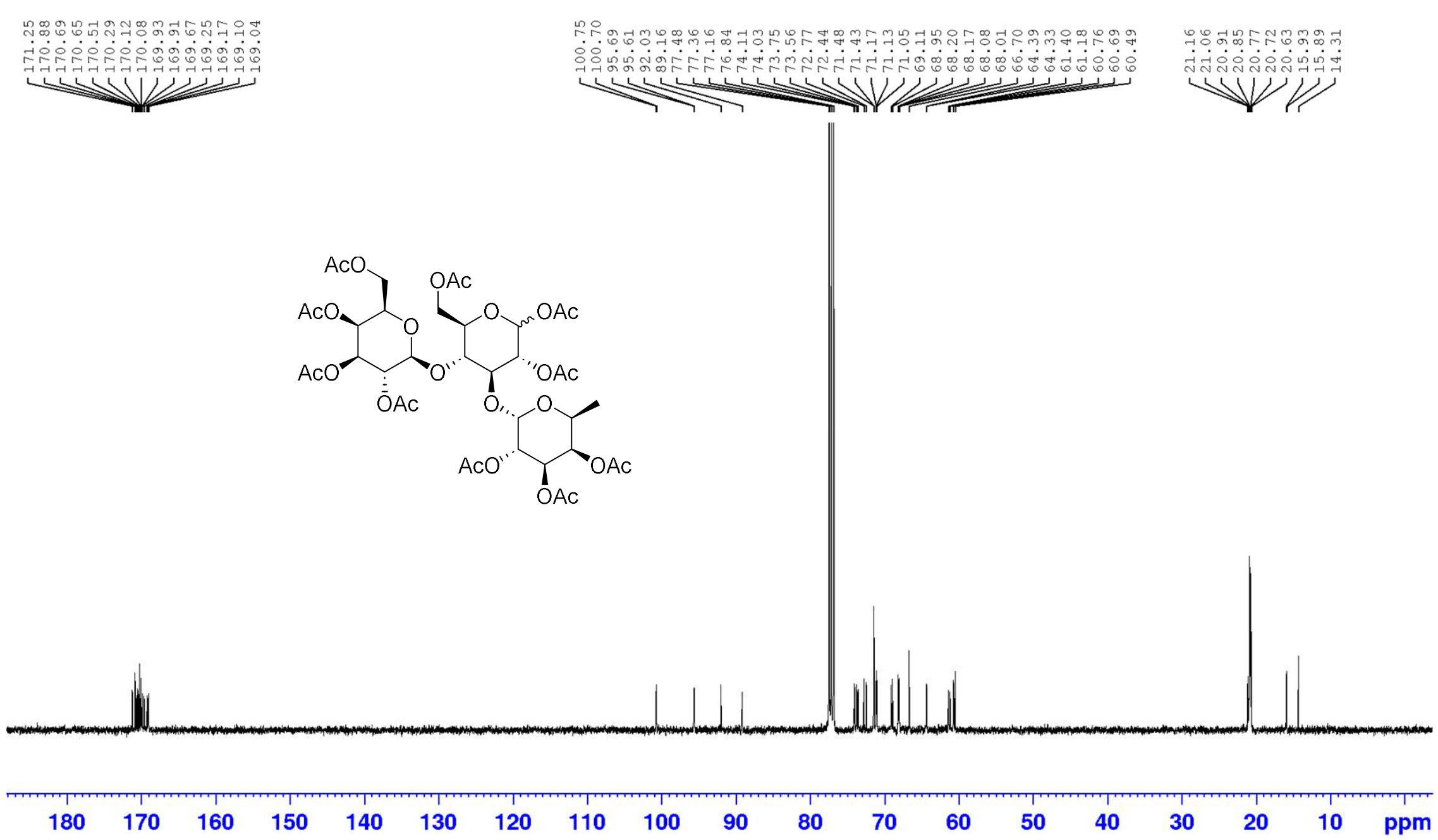

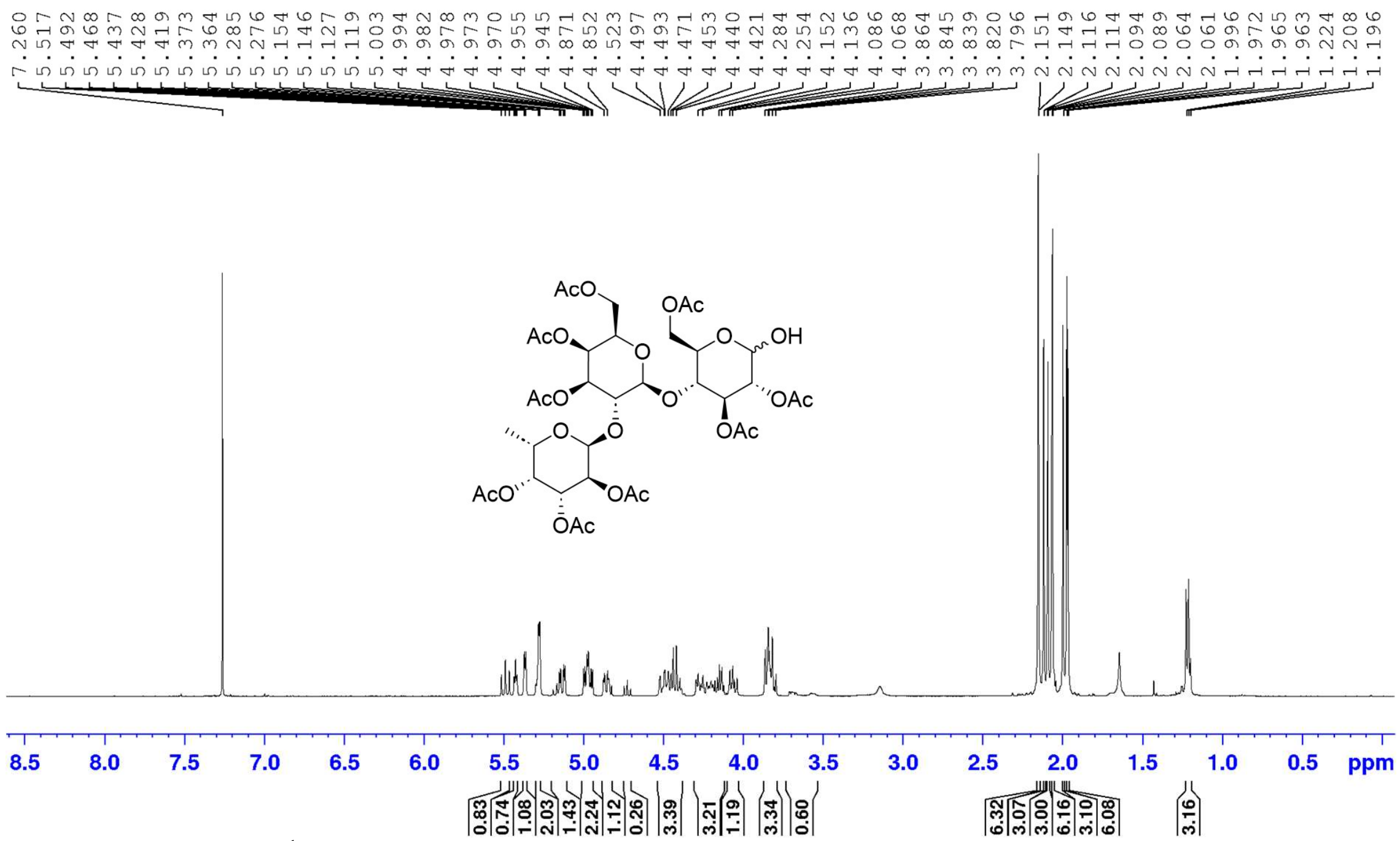

^{1}H NMR (400 MHz, $CDCl_3$) spectrum of compound **2a**.

^{13}C NMR (100 MHz, $CDCl_3$) spectrum of compound **2a.**

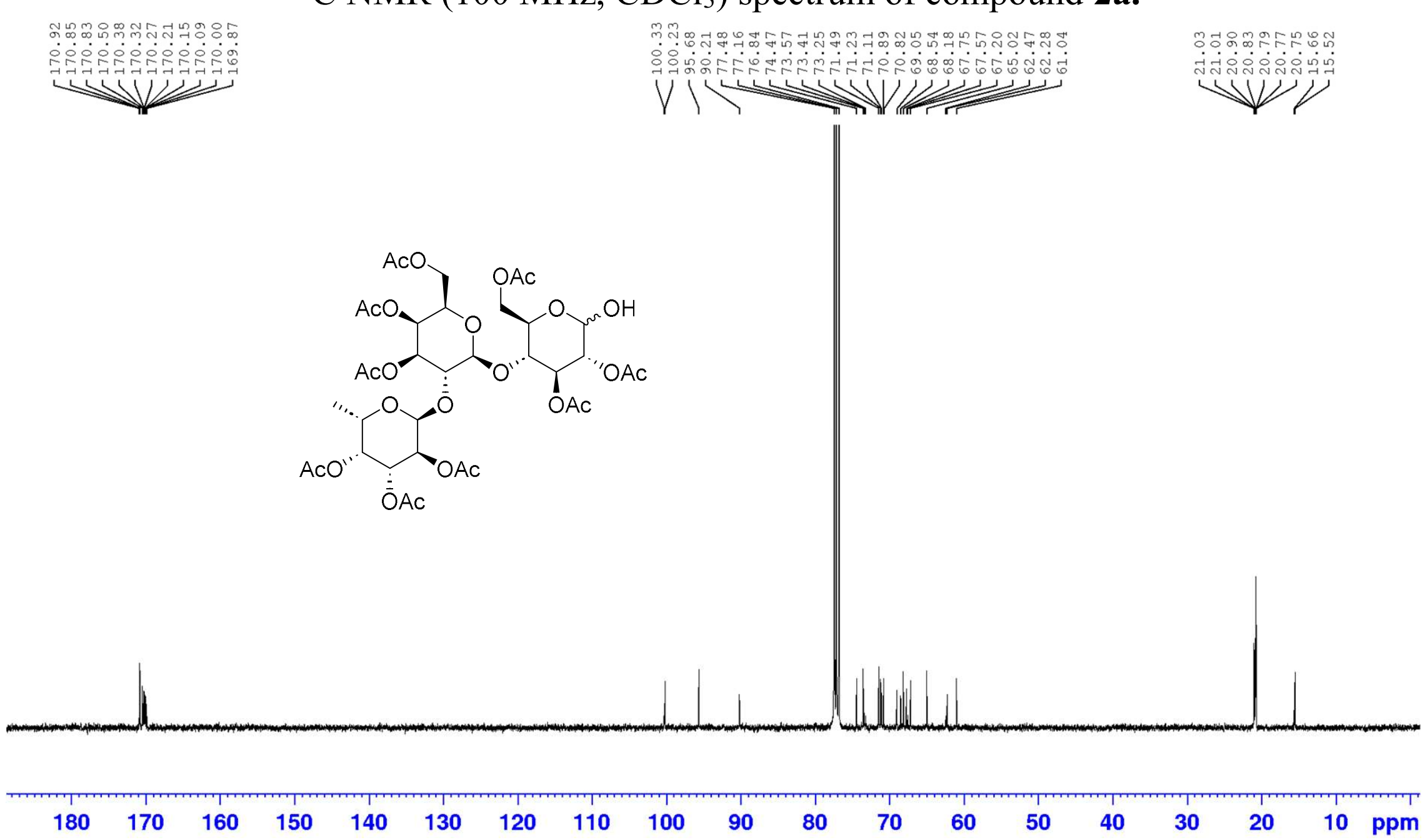

^{1}H NMR (400 MHz, $CDCl_3$) spectrum of compound **2b.**

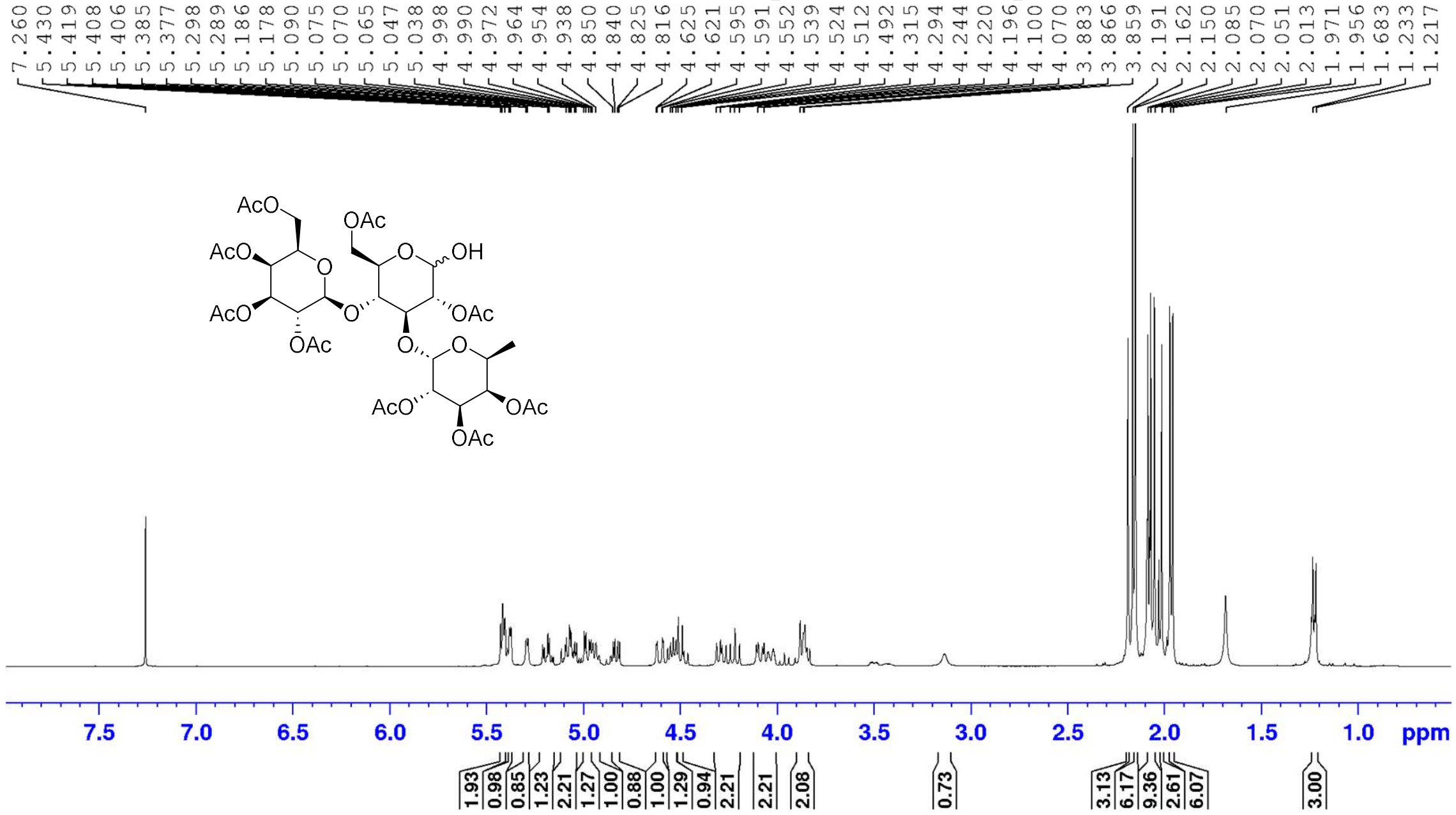

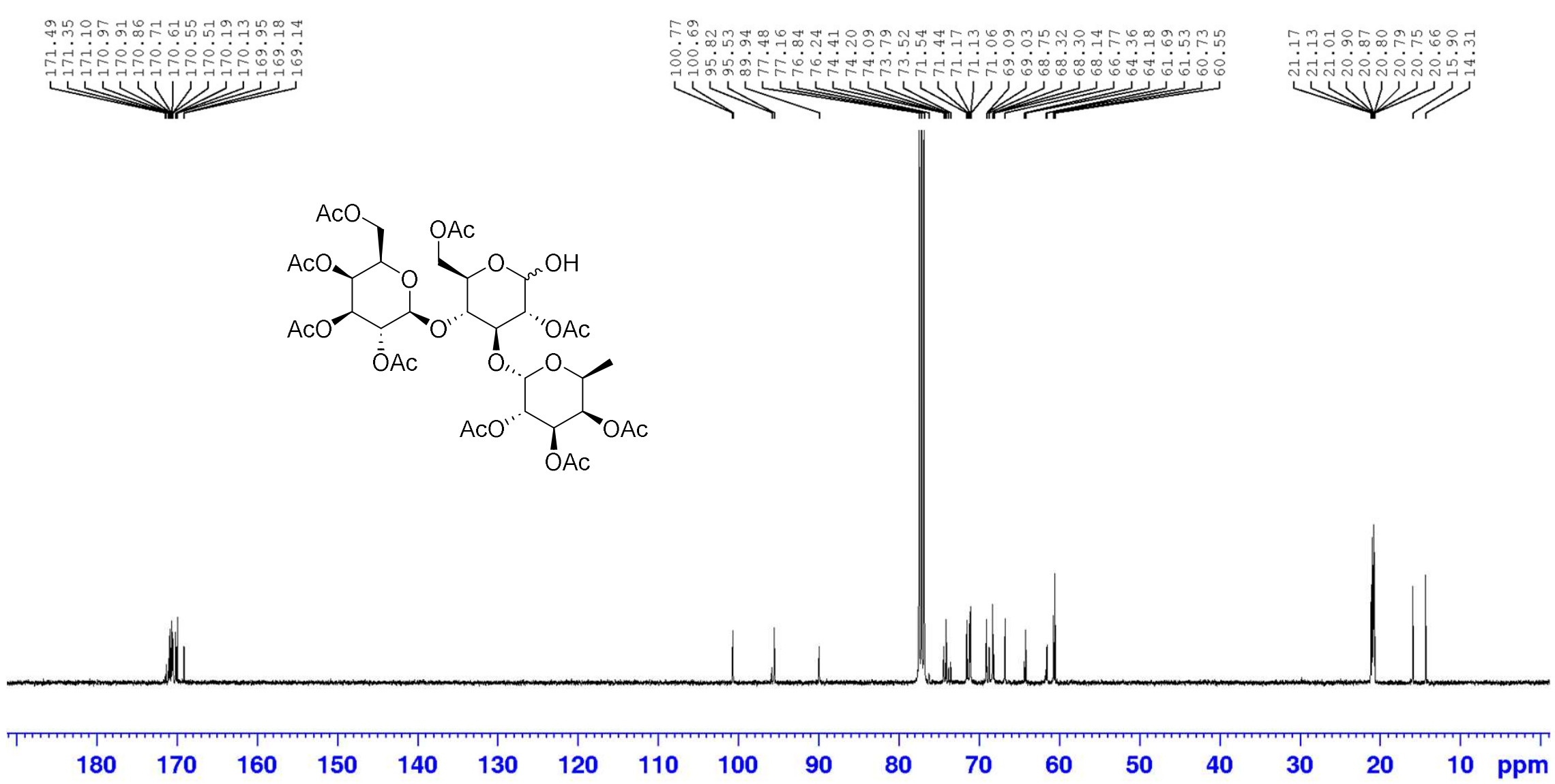

^{13}C NMR (100 MHz, $CDCl_3$) spectrum of compound **2b.**

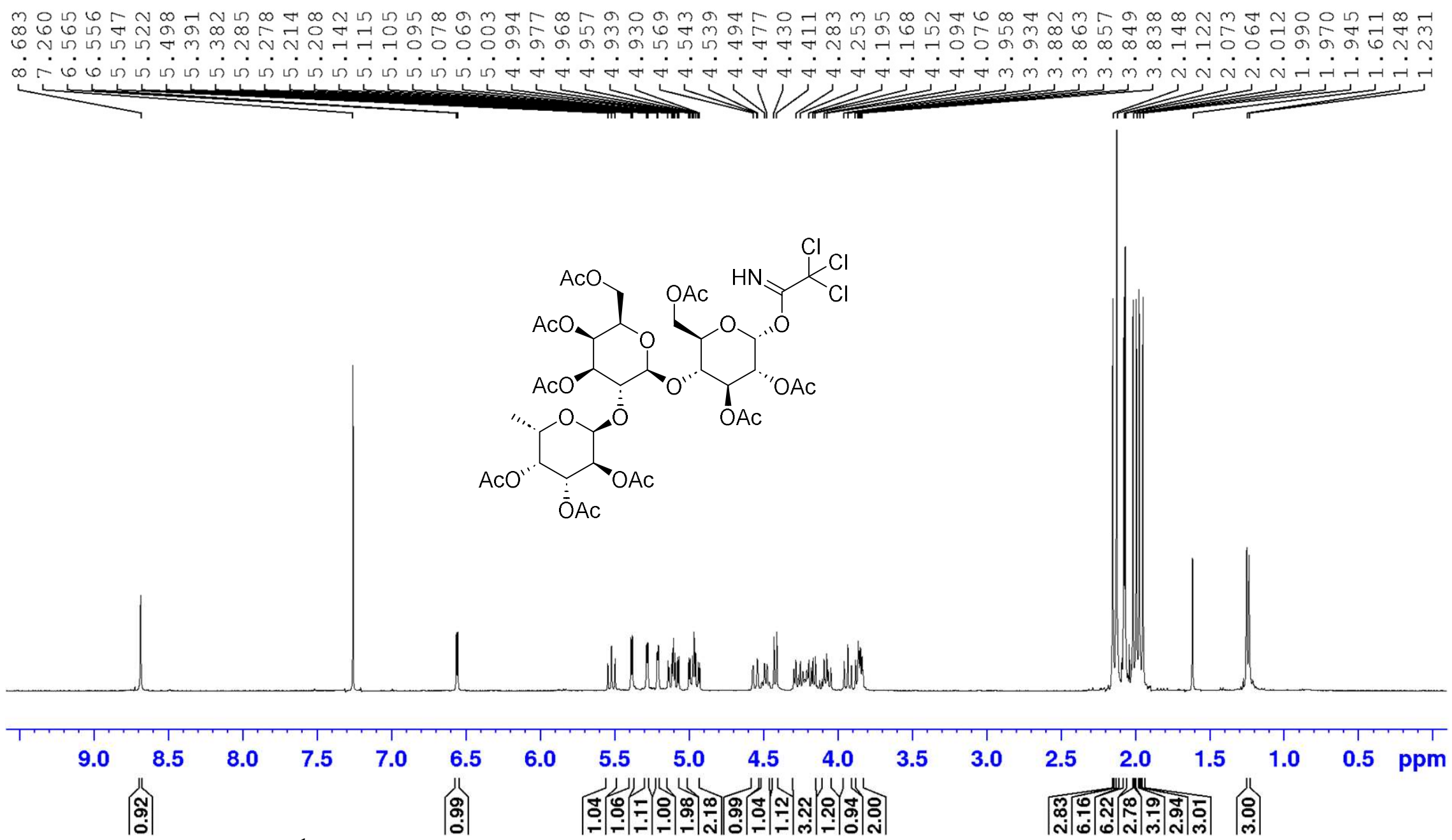

^{1}H NMR (400 MHz, $CDCl_3$) spectrum of compound **3a.**

^{13}C NMR (100 MHz, $CDCl_3$) spectrum of compound **3a.**

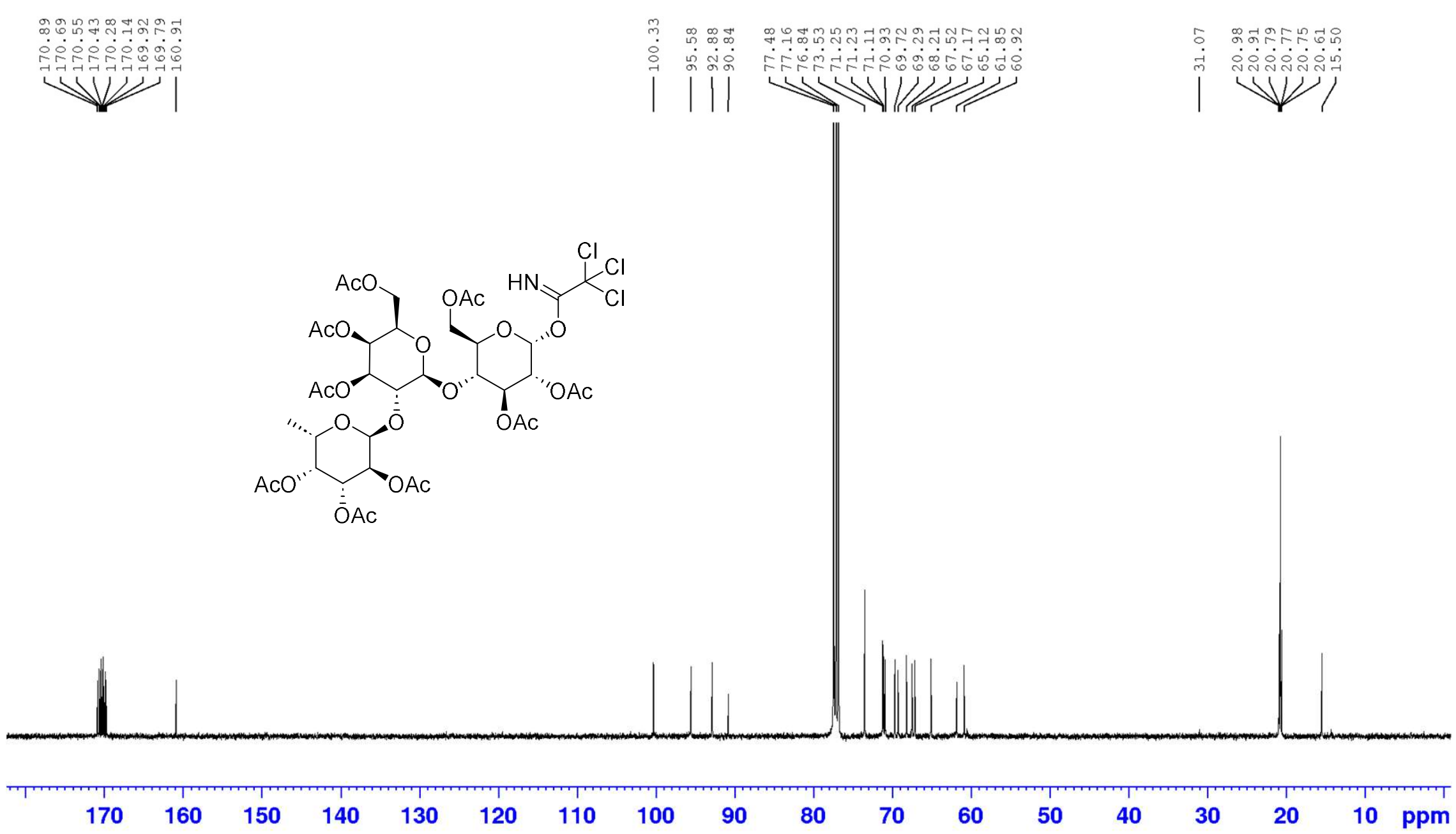

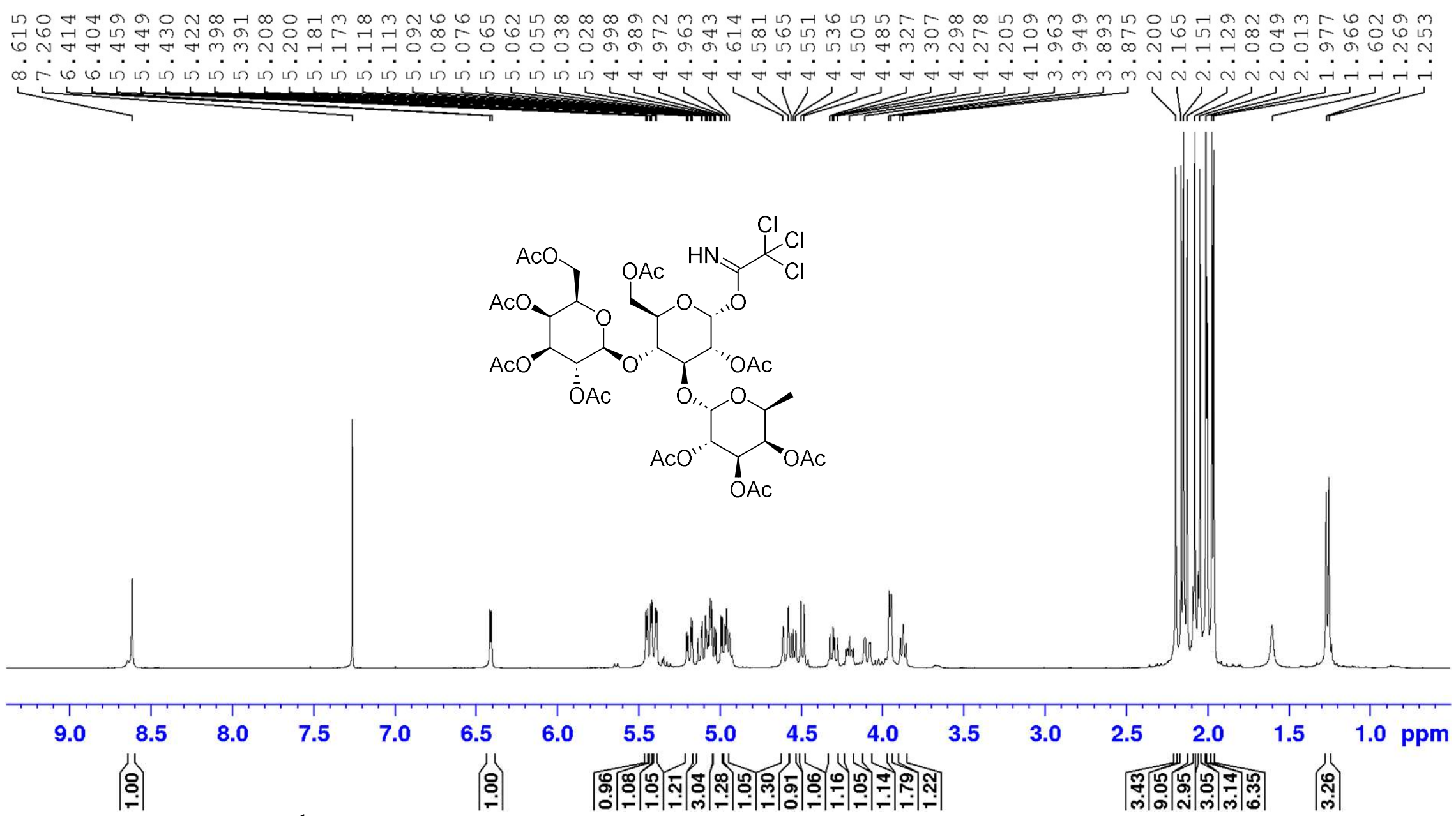

^{1}H NMR (400 MHz, $CDCl_3$) spectrum of compound **3b.**

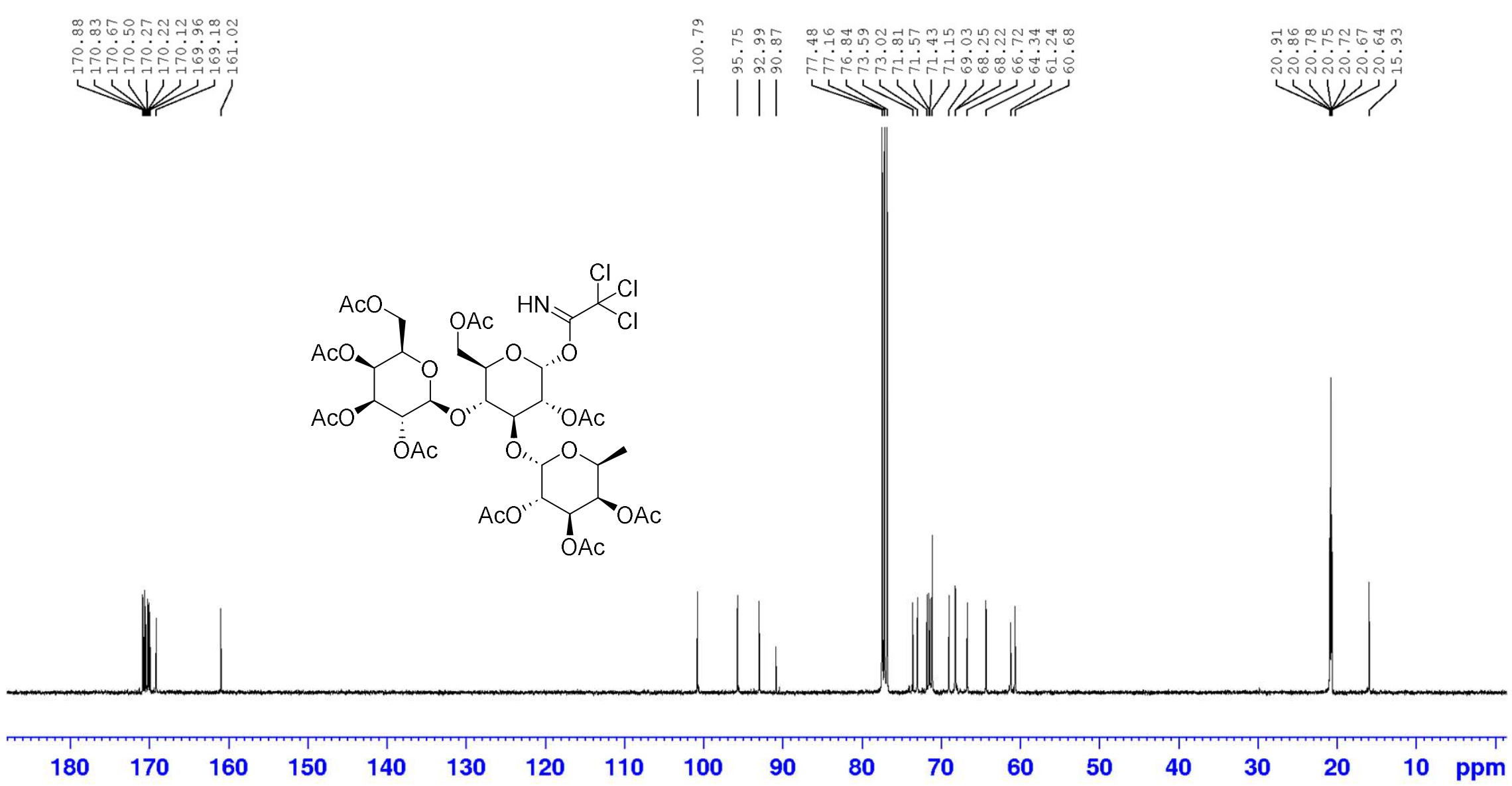

^{13}C NMR (100 MHz, $CDCl_3$) spectrum of compound **3b.**

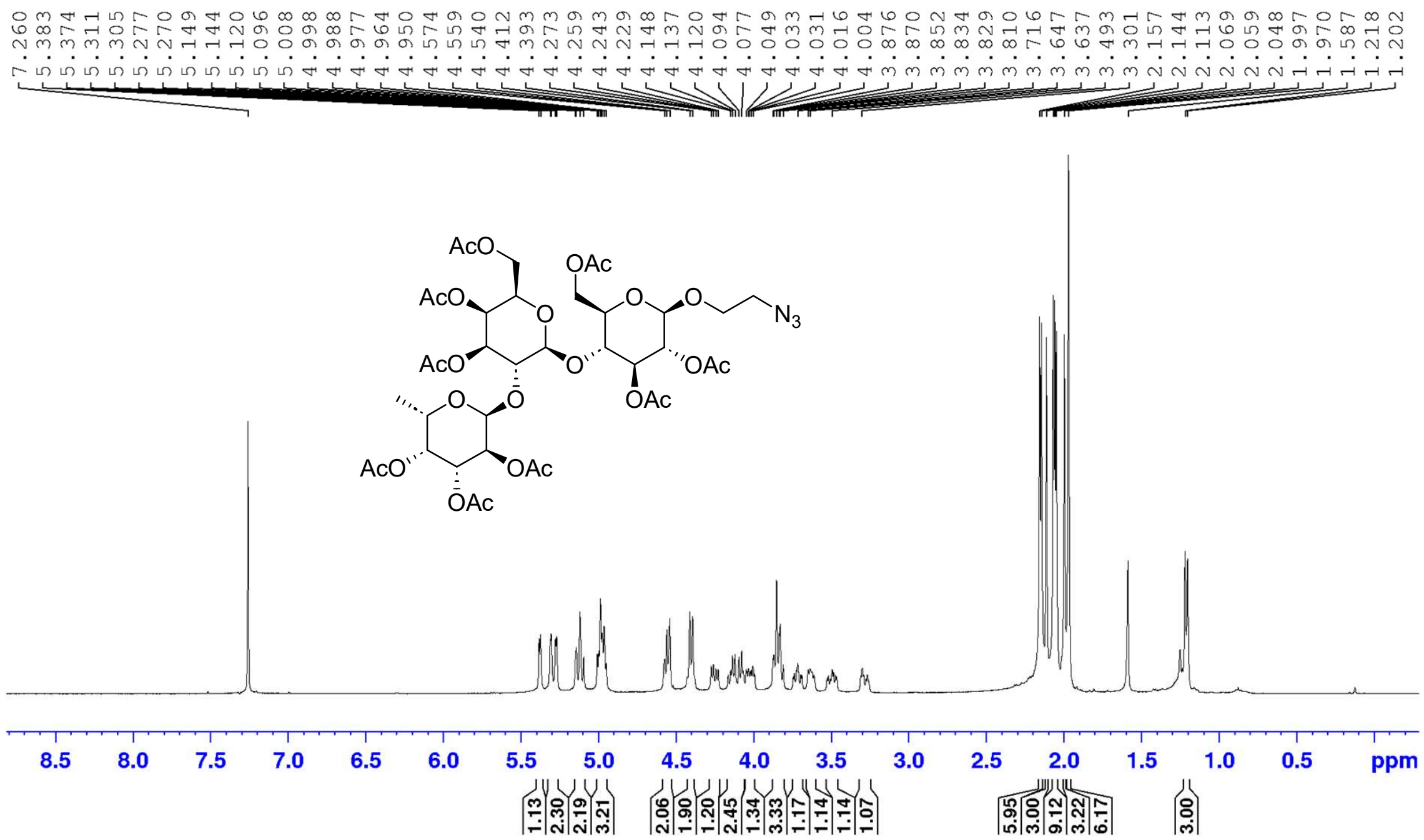

^{1}H NMR (400 MHz, $CDCl_3$) spectrum of compound **4a.**

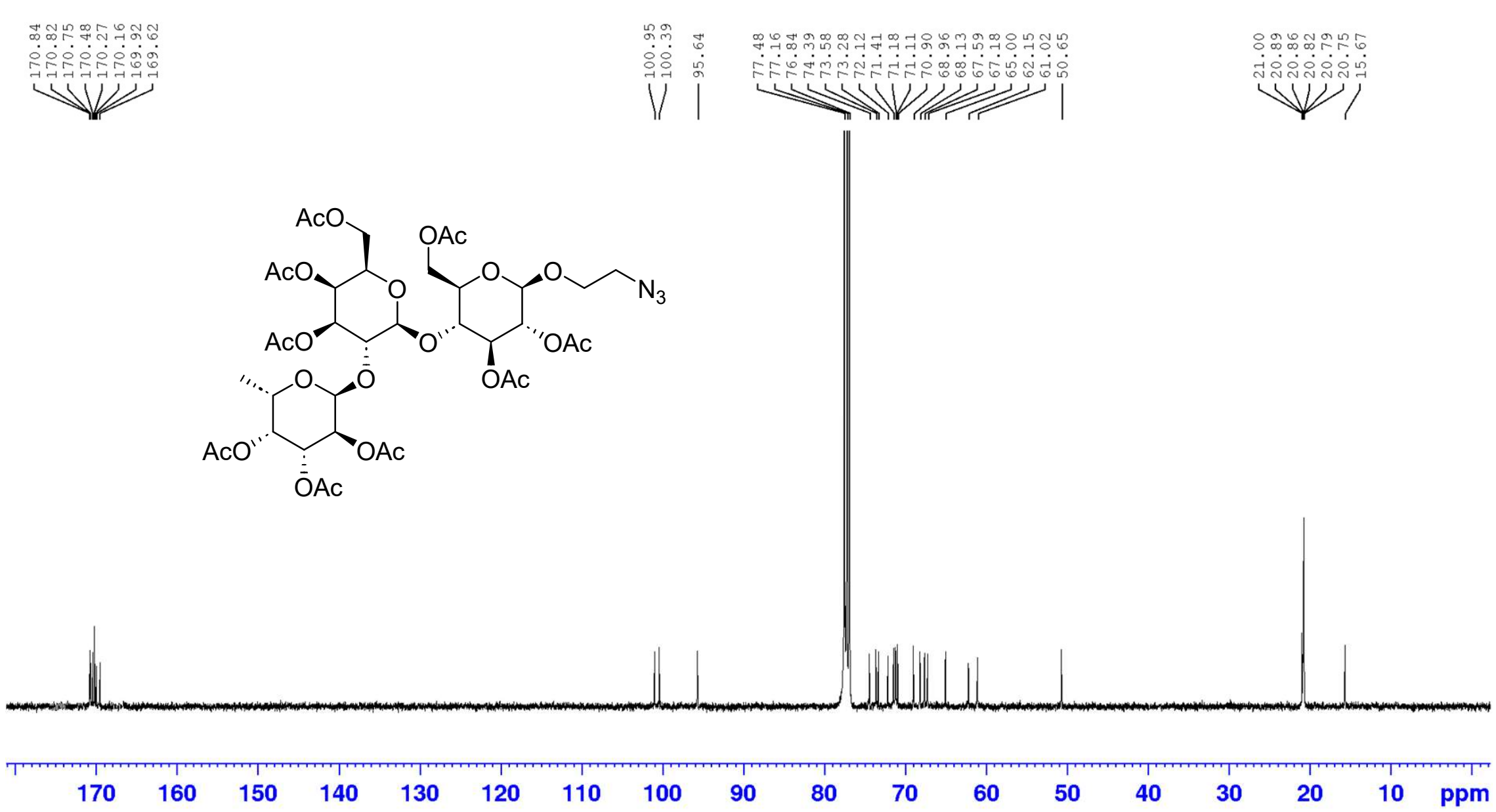

^{13}C NMR (100 MHz, $CDCl_3$) spectrum of compound **4a.**

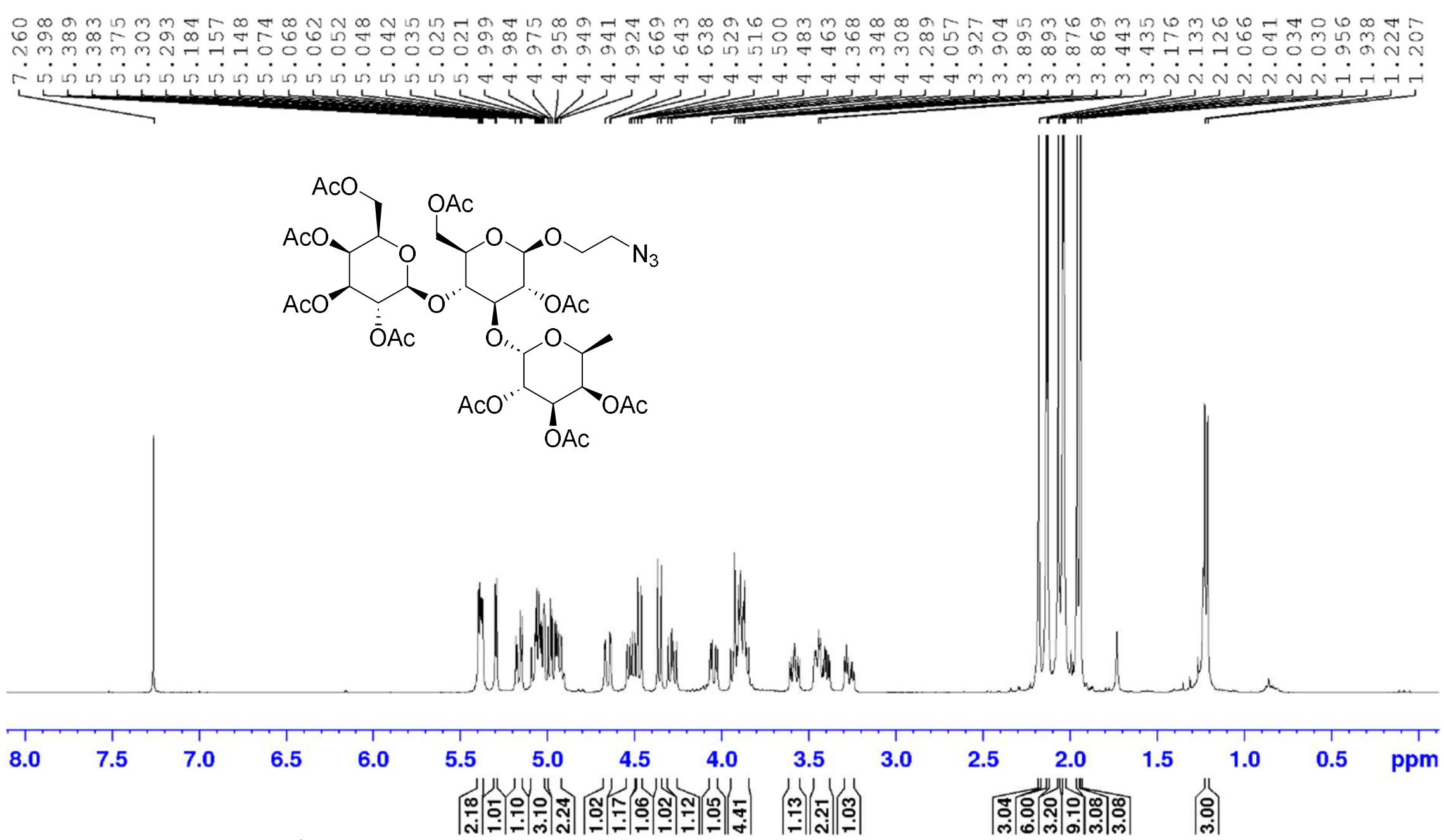

^{1}H NMR (400 MHz, $CDCl_3$) spectrum of compound **4b.**

^{13}C NMR (100 MHz, $CDCl_3$) spectrum of compound **4b.**

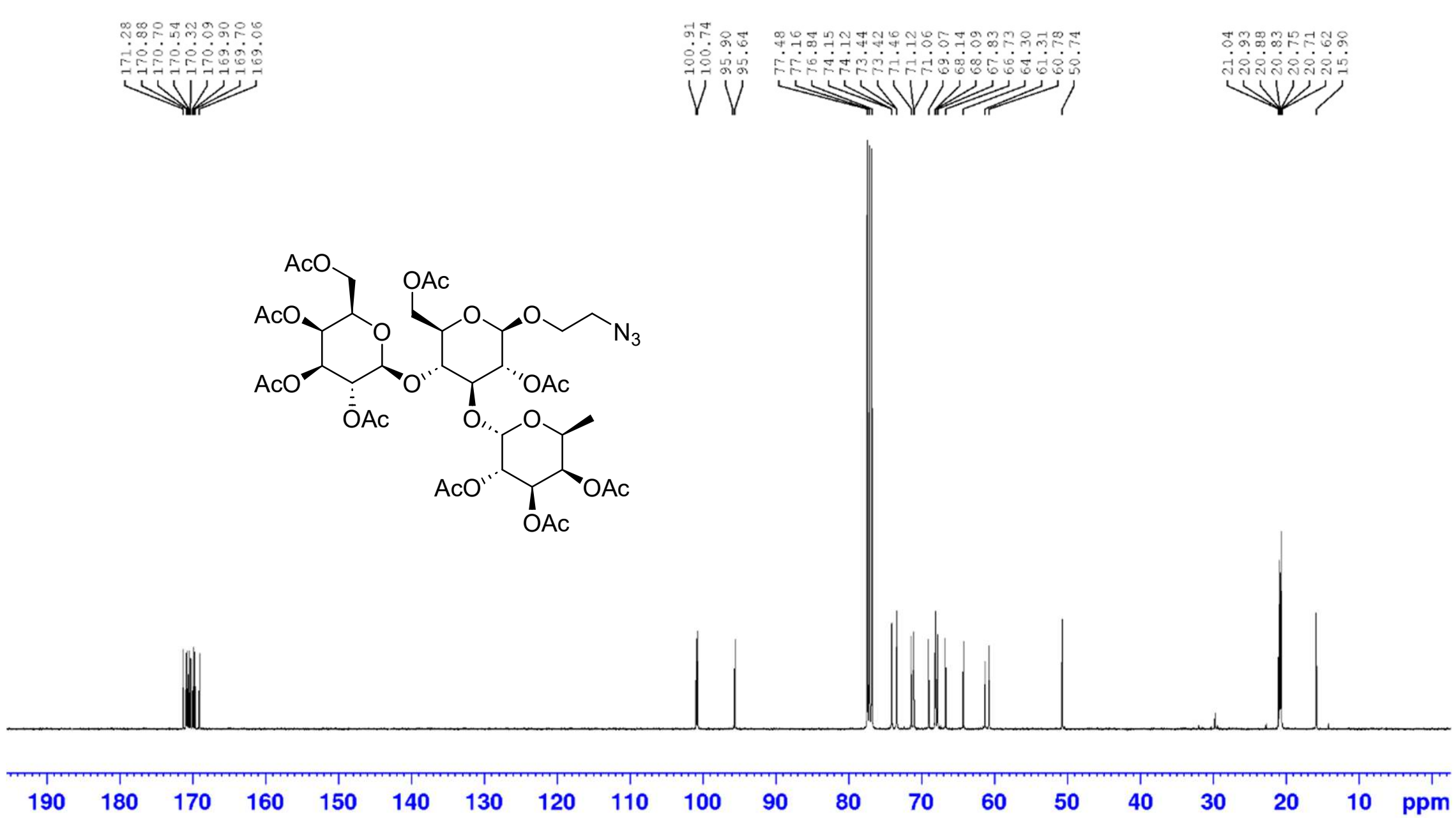

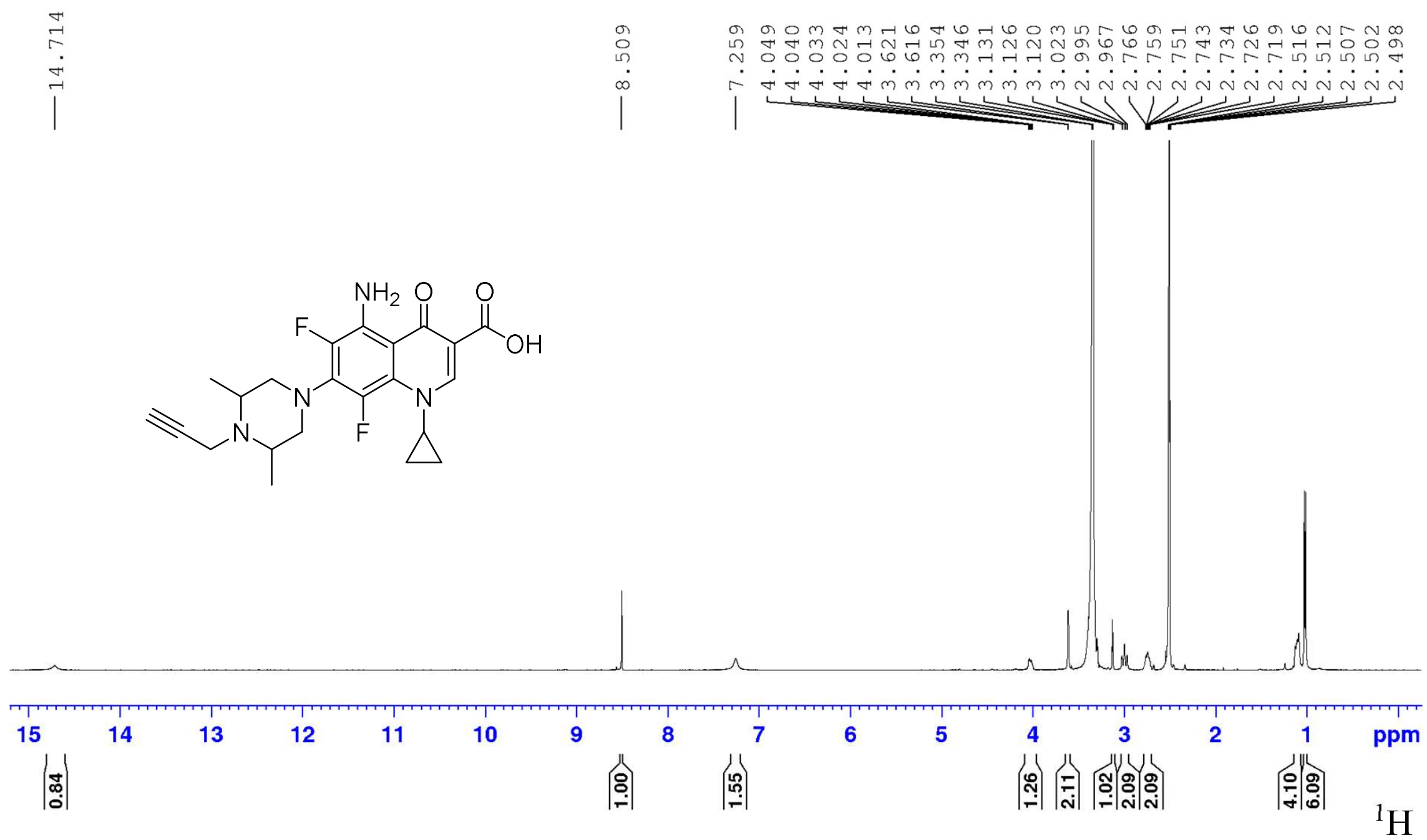

NMR (400 MHz, $(CD_3)_2SO$) spectrum of compound **7.**

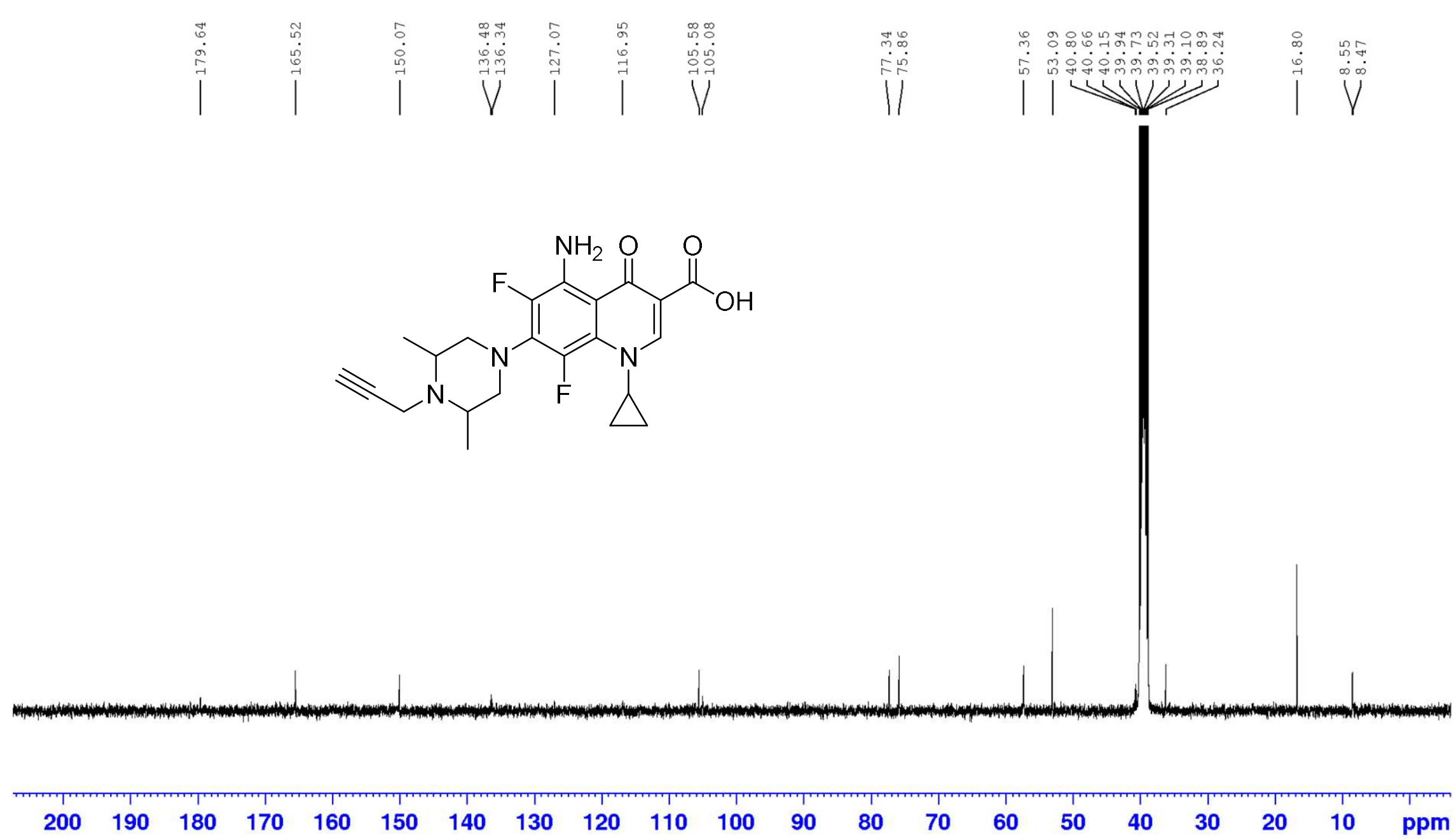

^{13}C NMR (100 MHz, $(CD_3)_2SO$) spectrum of compound **7.**

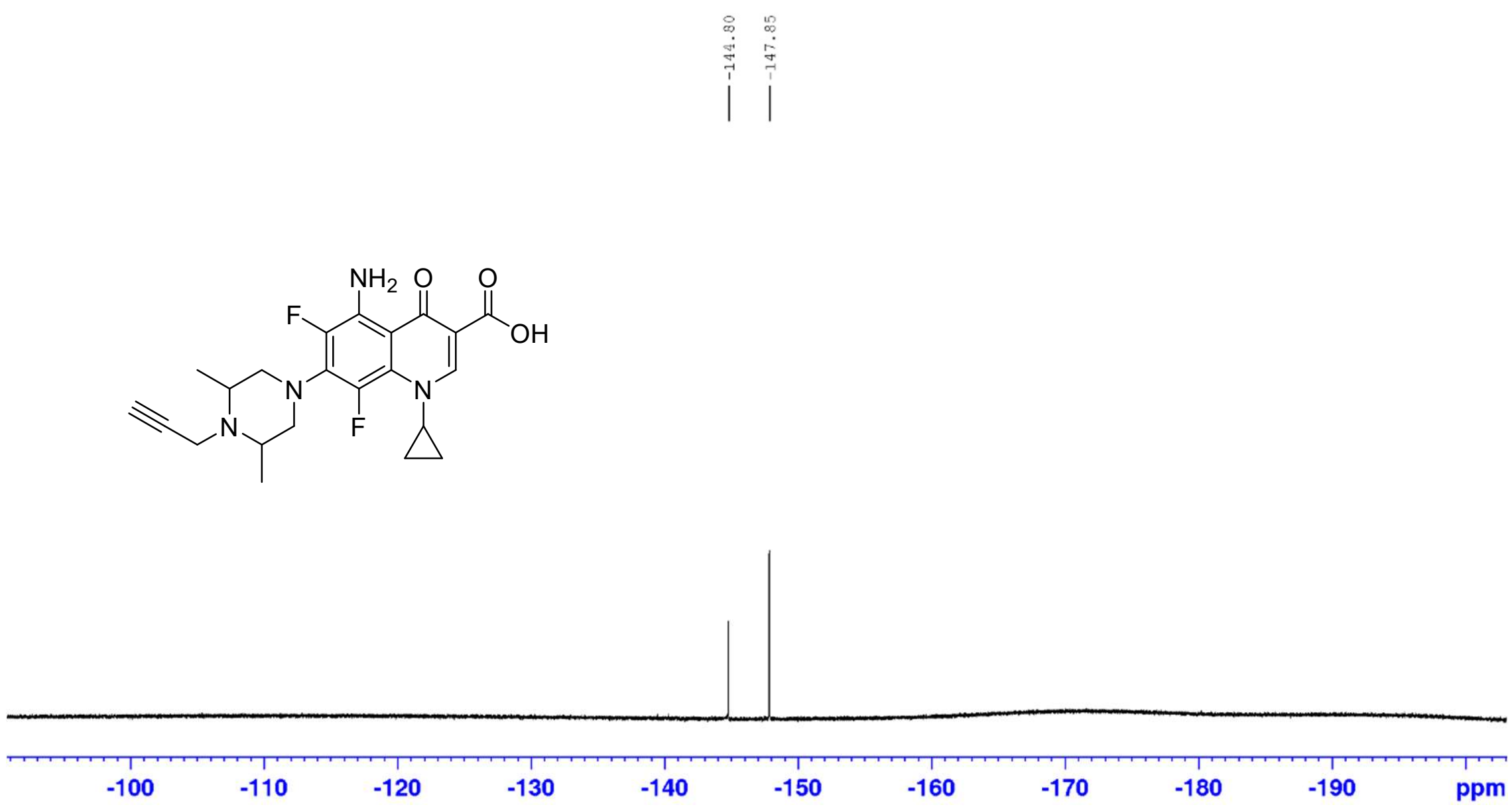

^{19}F NMR (376 MHz, $(CD_3)_2SO$) spectrum of compound **7.**

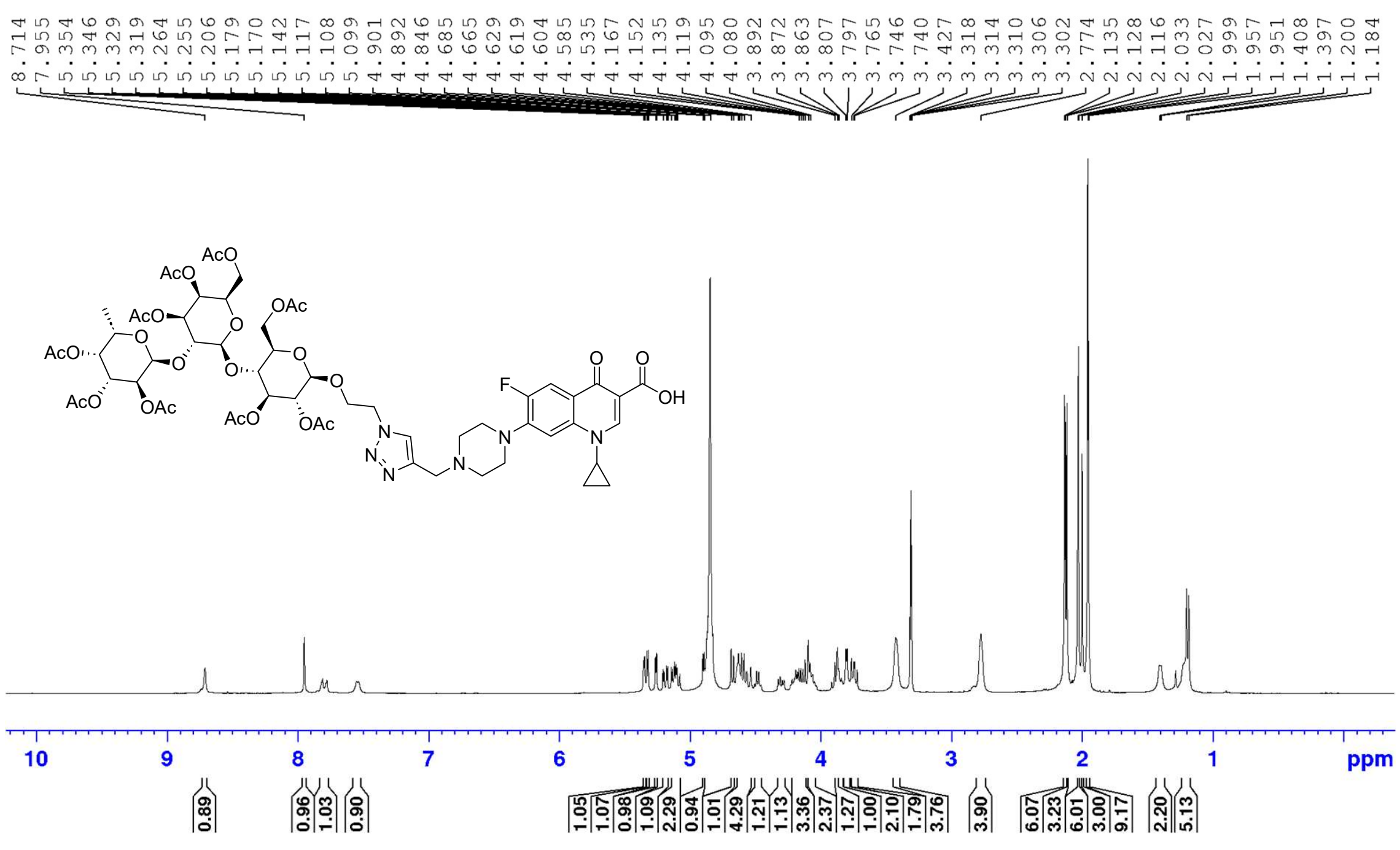

^{1}H NMR (400 MHz, $CDCl_3$) spectrum of compound **8a.**

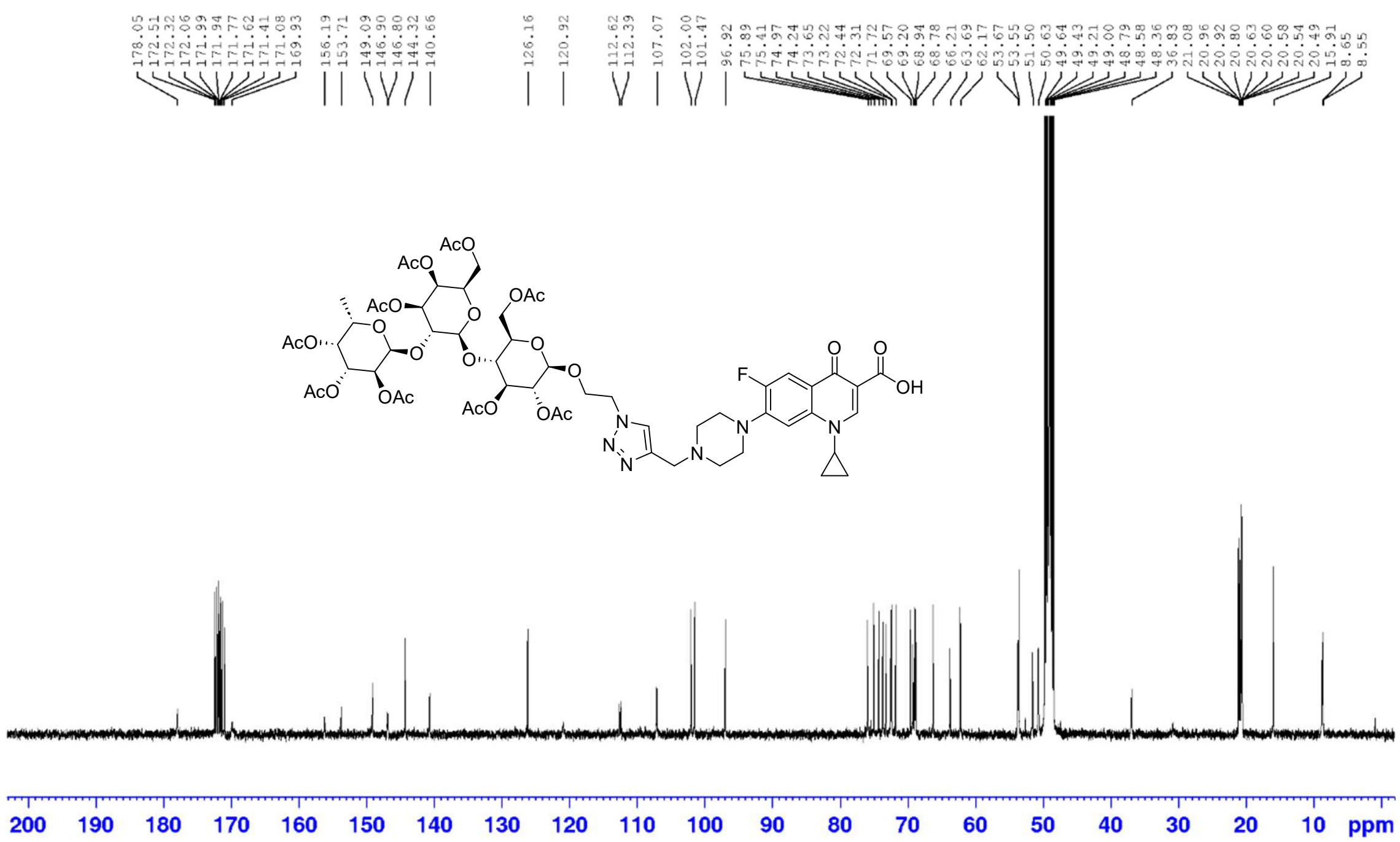

^{13}C NMR (100 MHz, CD_3OD) spectrum of compound **8a.**

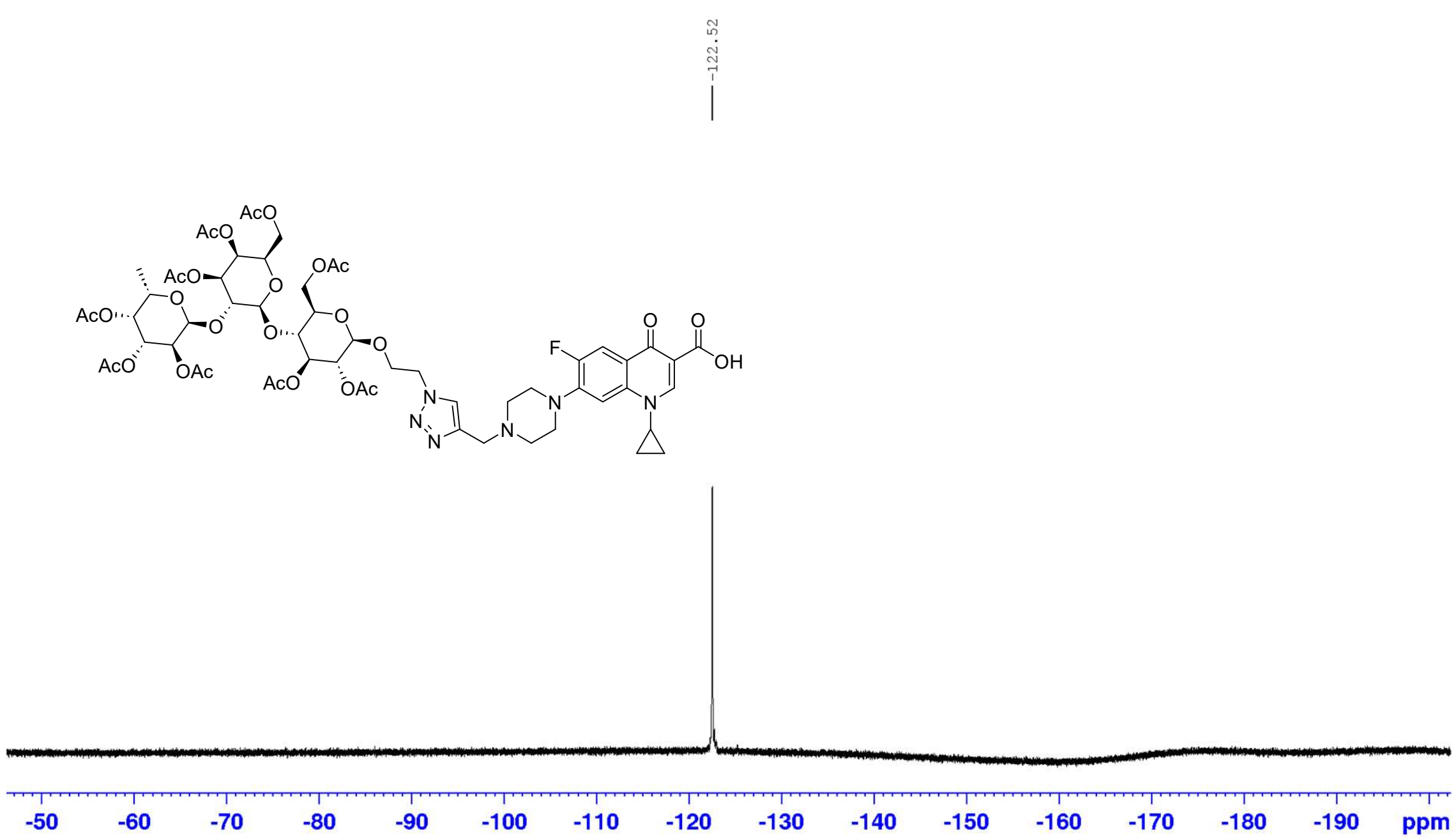

^{19}F NMR (376 MHz, CD_3OD) spectrum of compound **8a.**

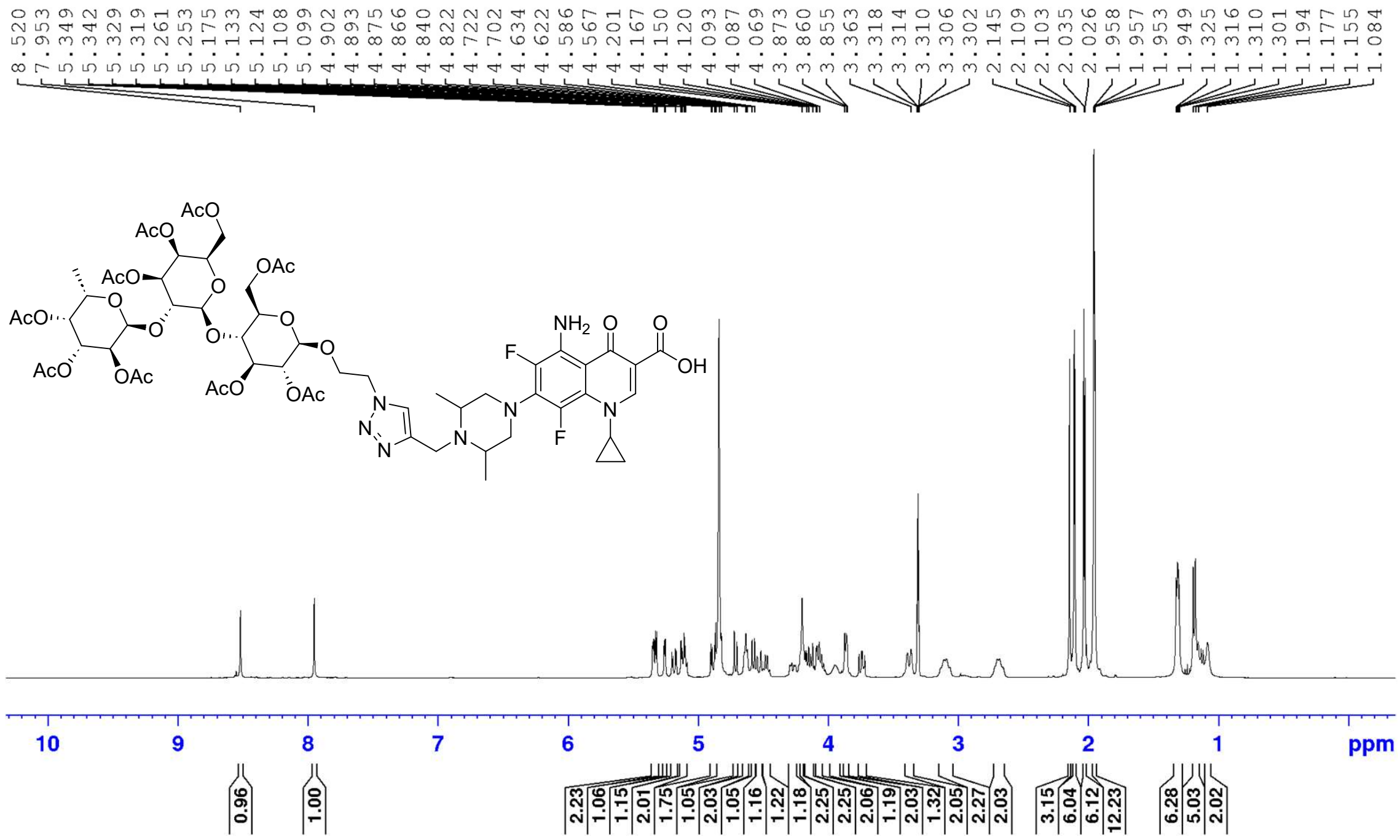

^{1}H NMR (400 MHz, CD3OD) spectrum of compound **8b.**

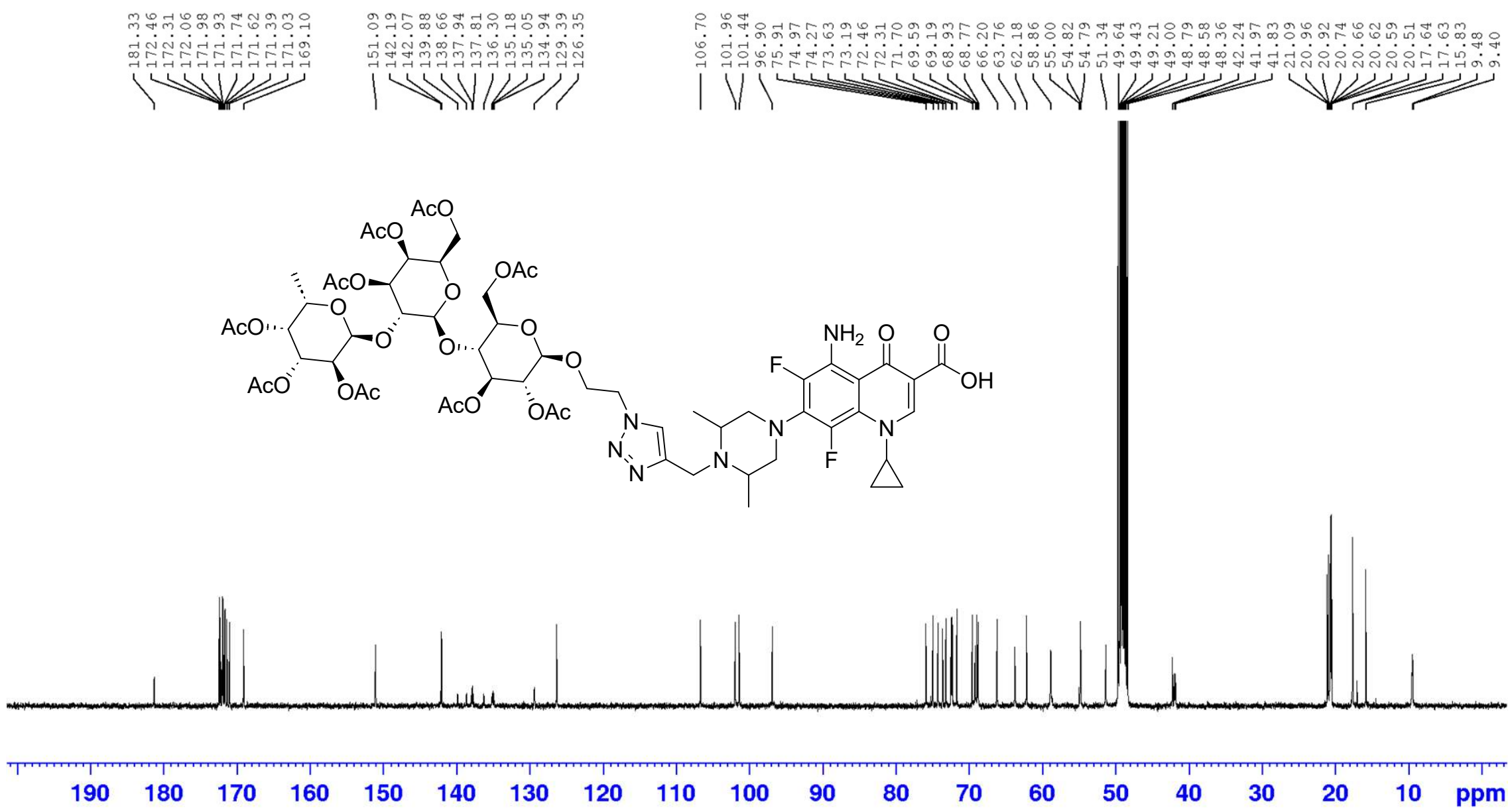

^{13}C NMR (100 MHz, CD3OD) spectrum of compound **8b.**

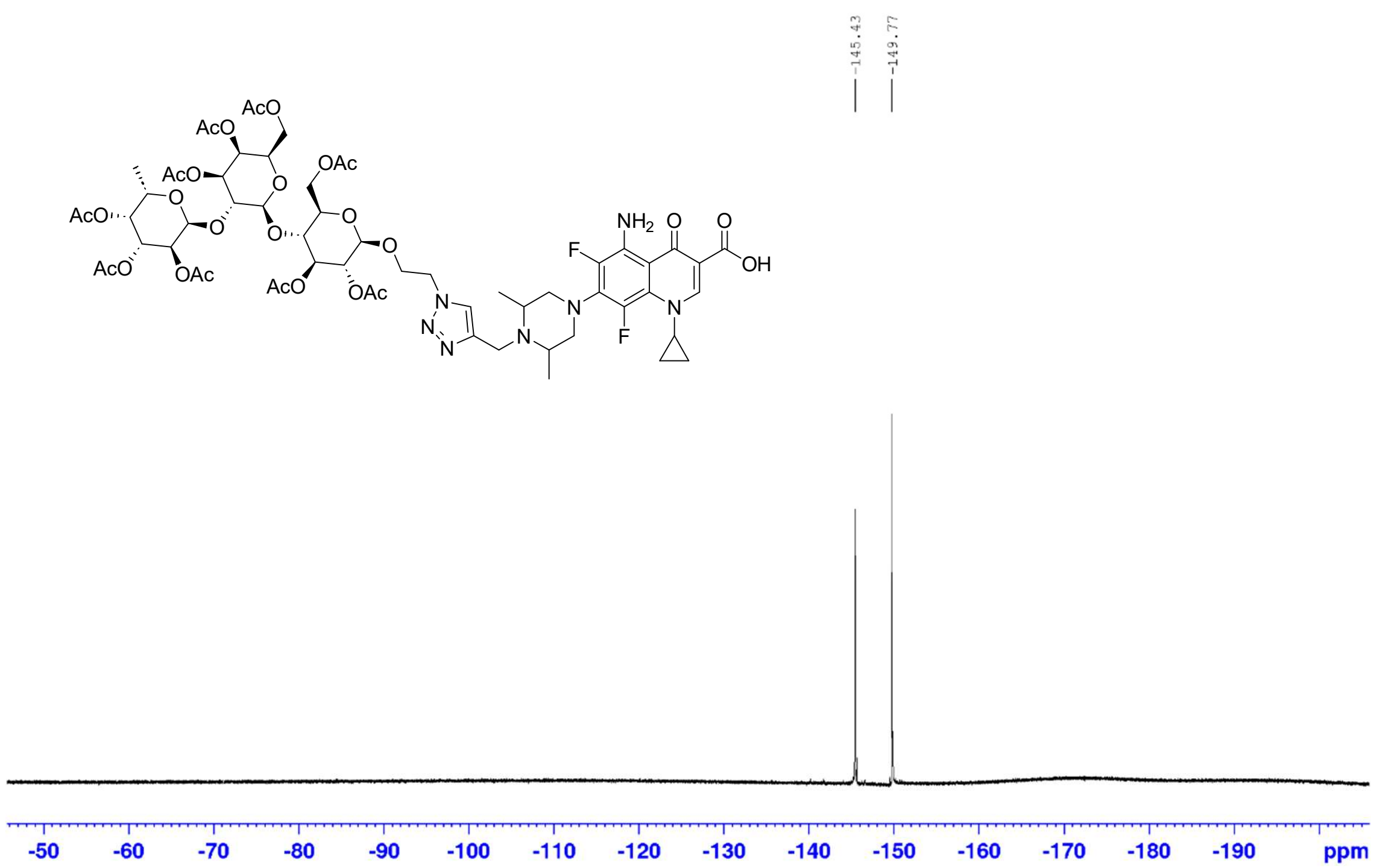

^{19}F NMR (376 MHz, CD_3OD) spectrum of compound **8b.**

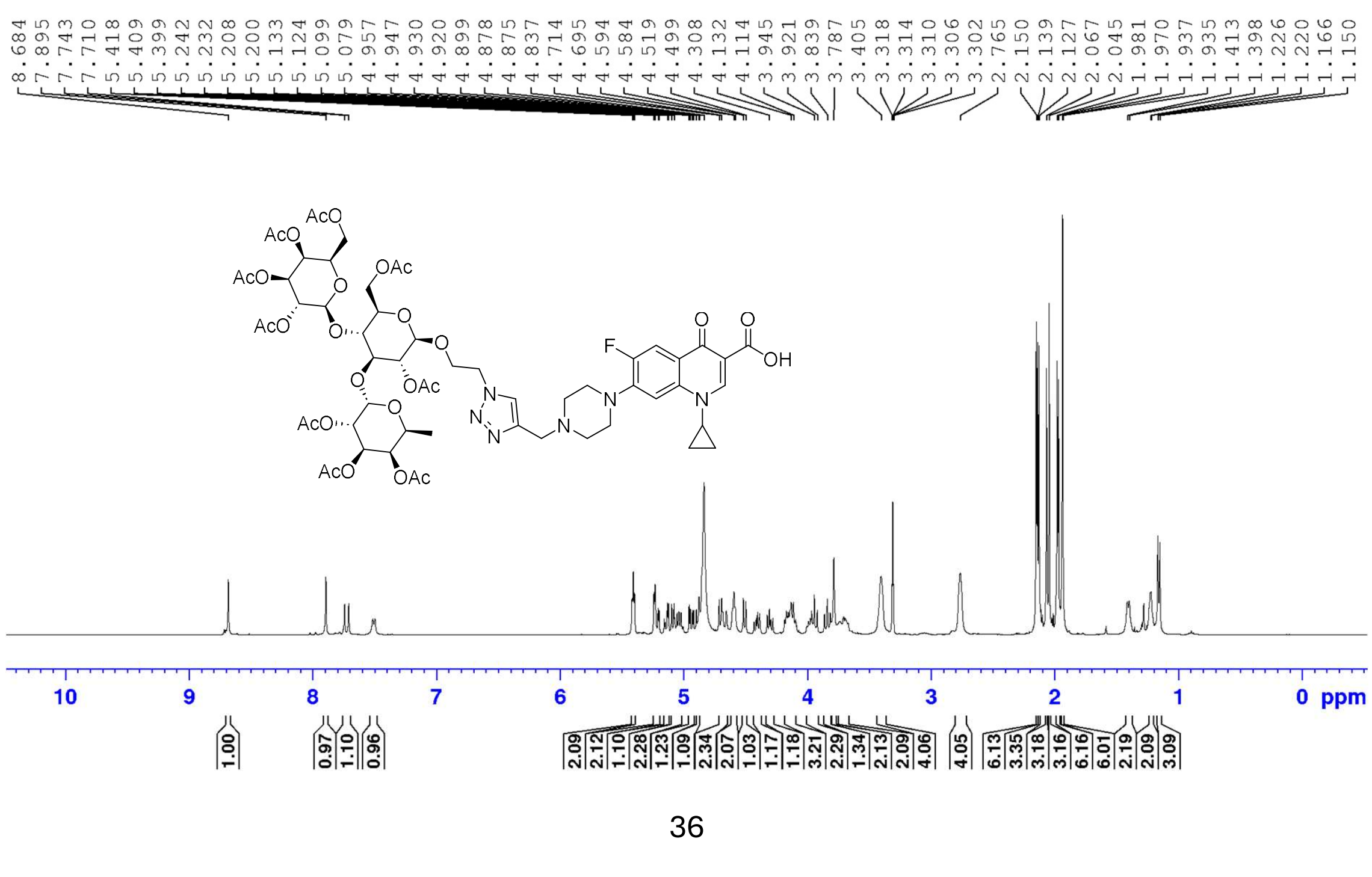

^{1}H NMR (400 MHz, CD_3OD) spectrum of compound **8c.**

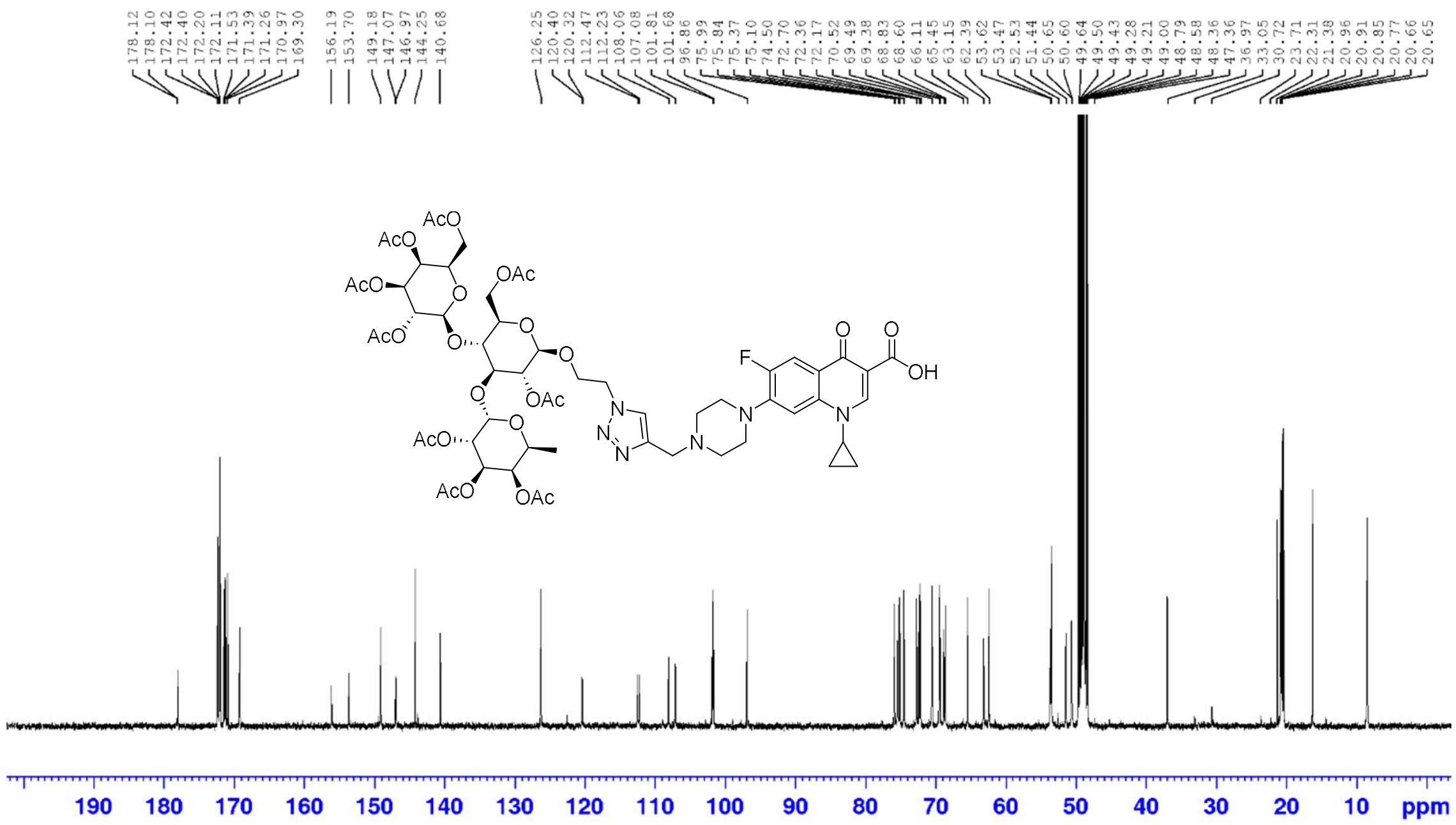

^{13}C NMR (100 MHz, CD_3OD) spectrum of compound **8c.**

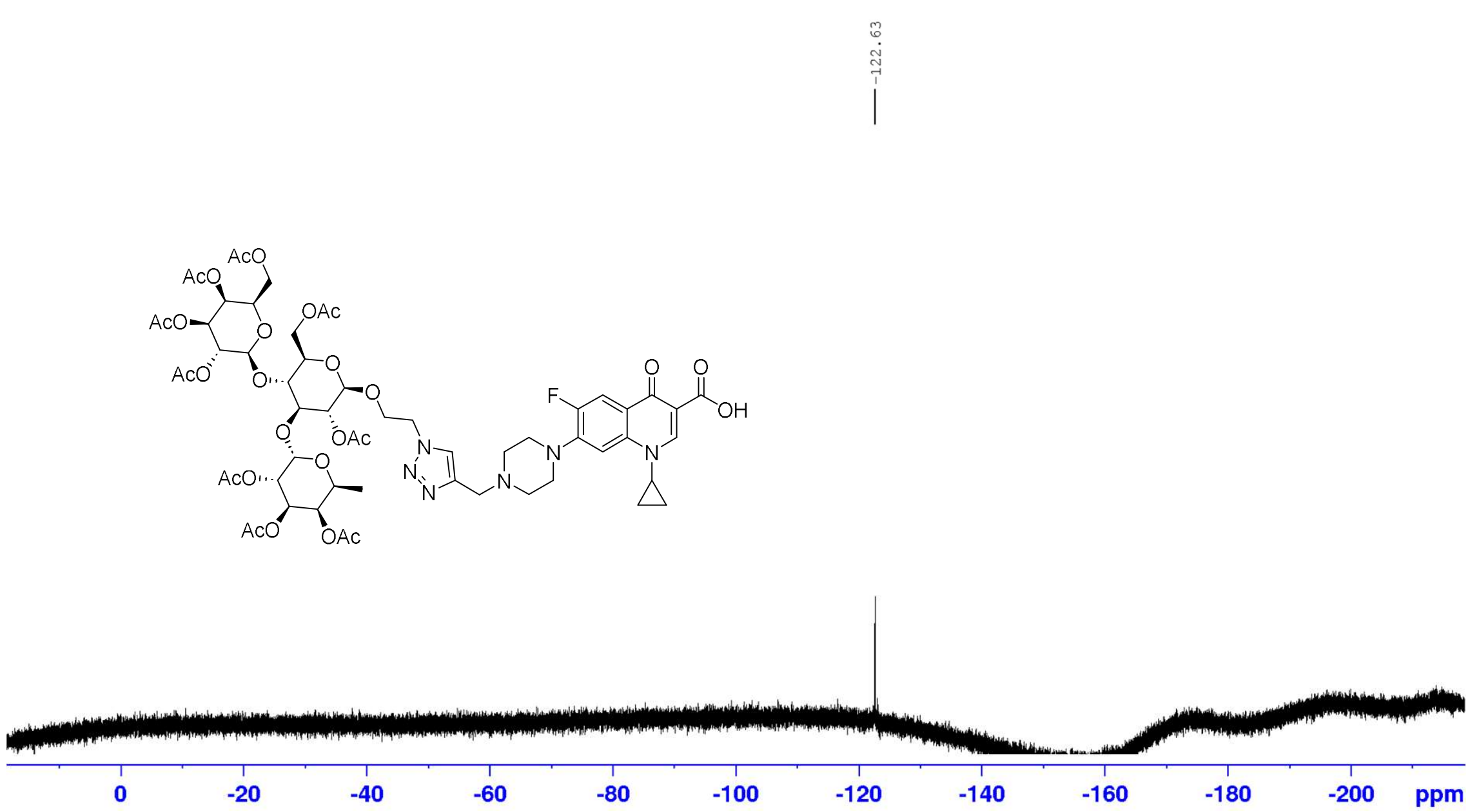

^{19}F NMR (376 MHz, CD_3OD) spectrum of compound **8c.**

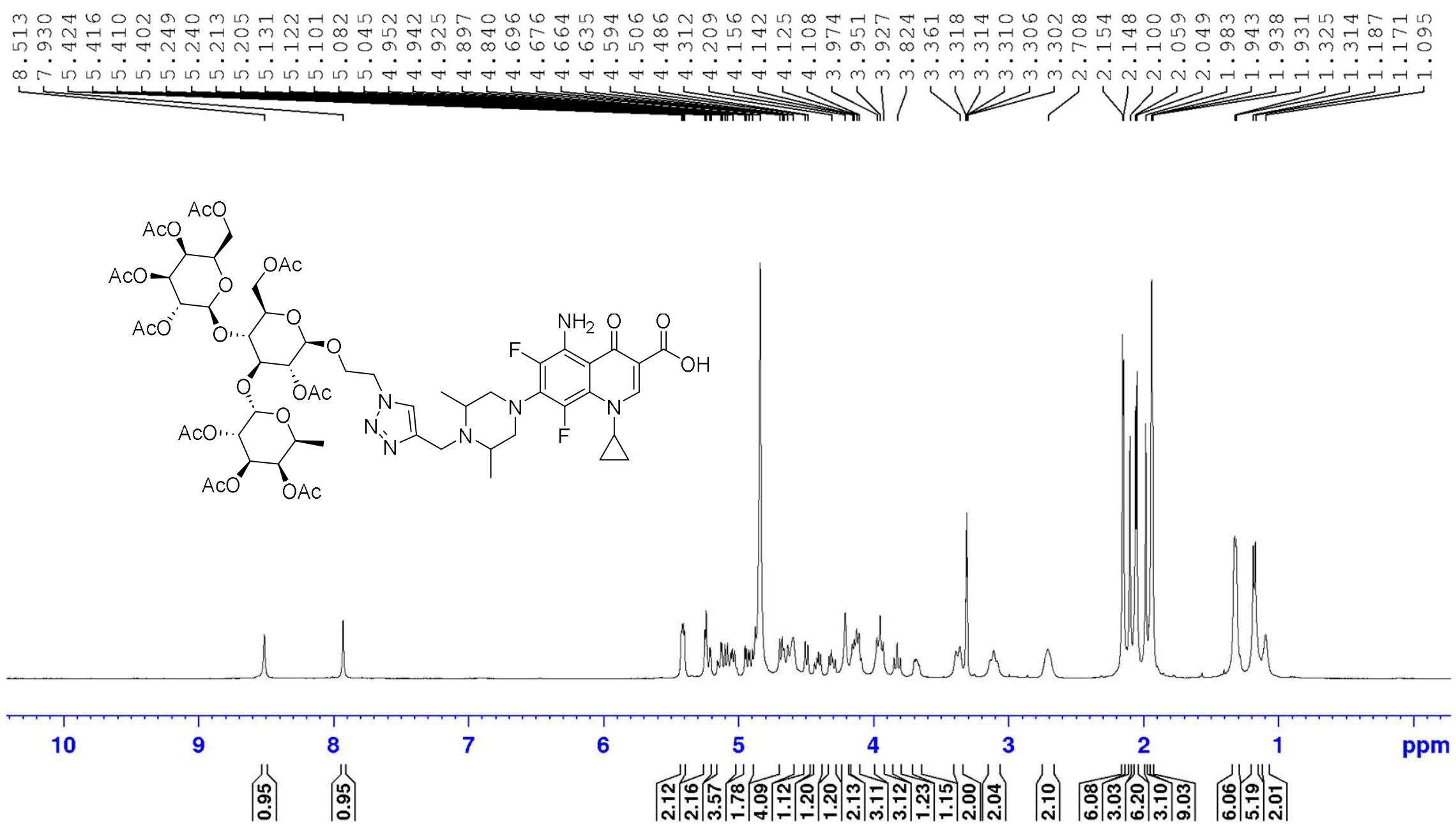

^{1}H NMR (400 MHz, CD_3OD) spectrum of compound **8d.**

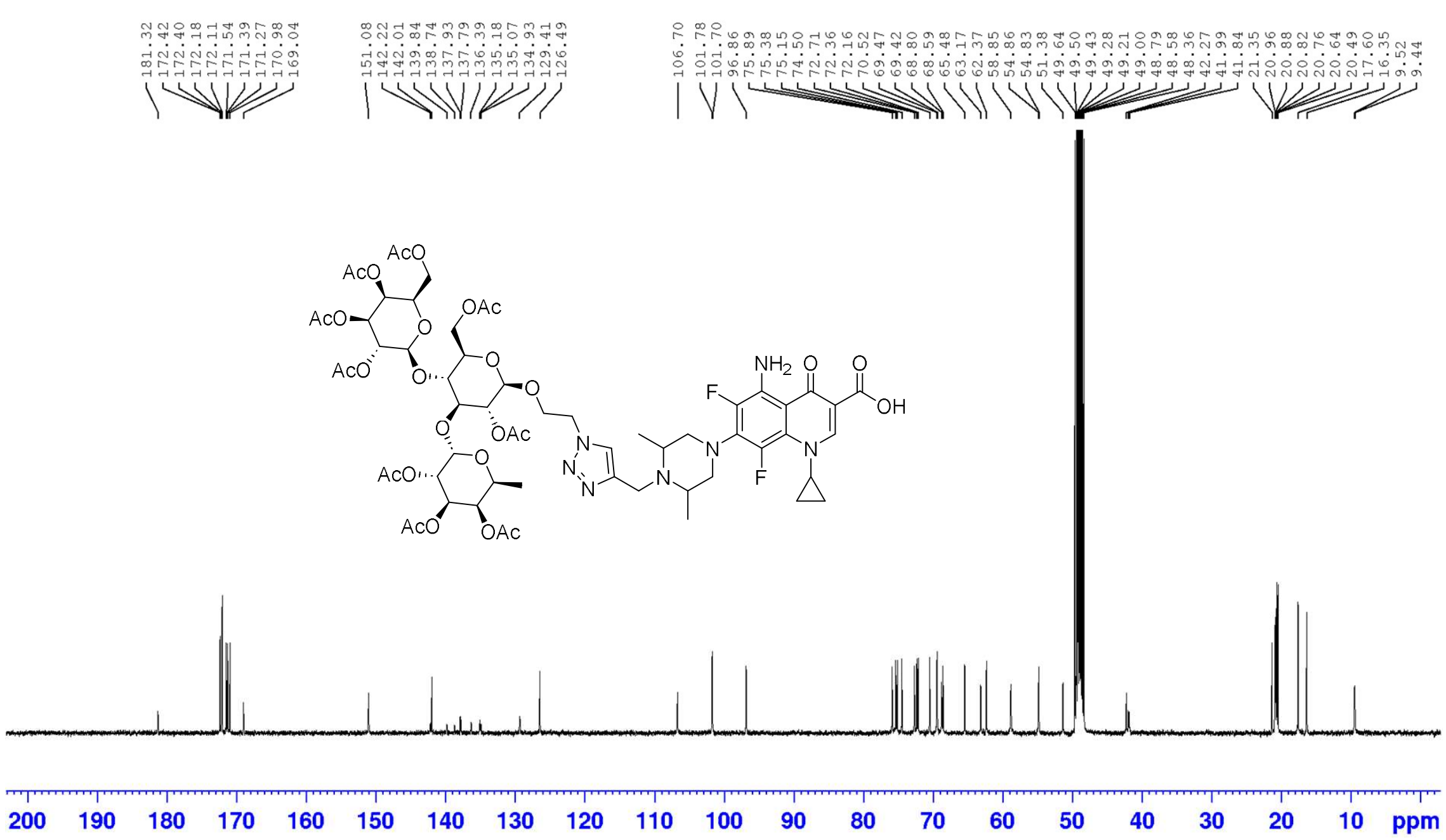

^{13}C NMR (100 MHz, CD_3OD) spectrum of compound **8d.**

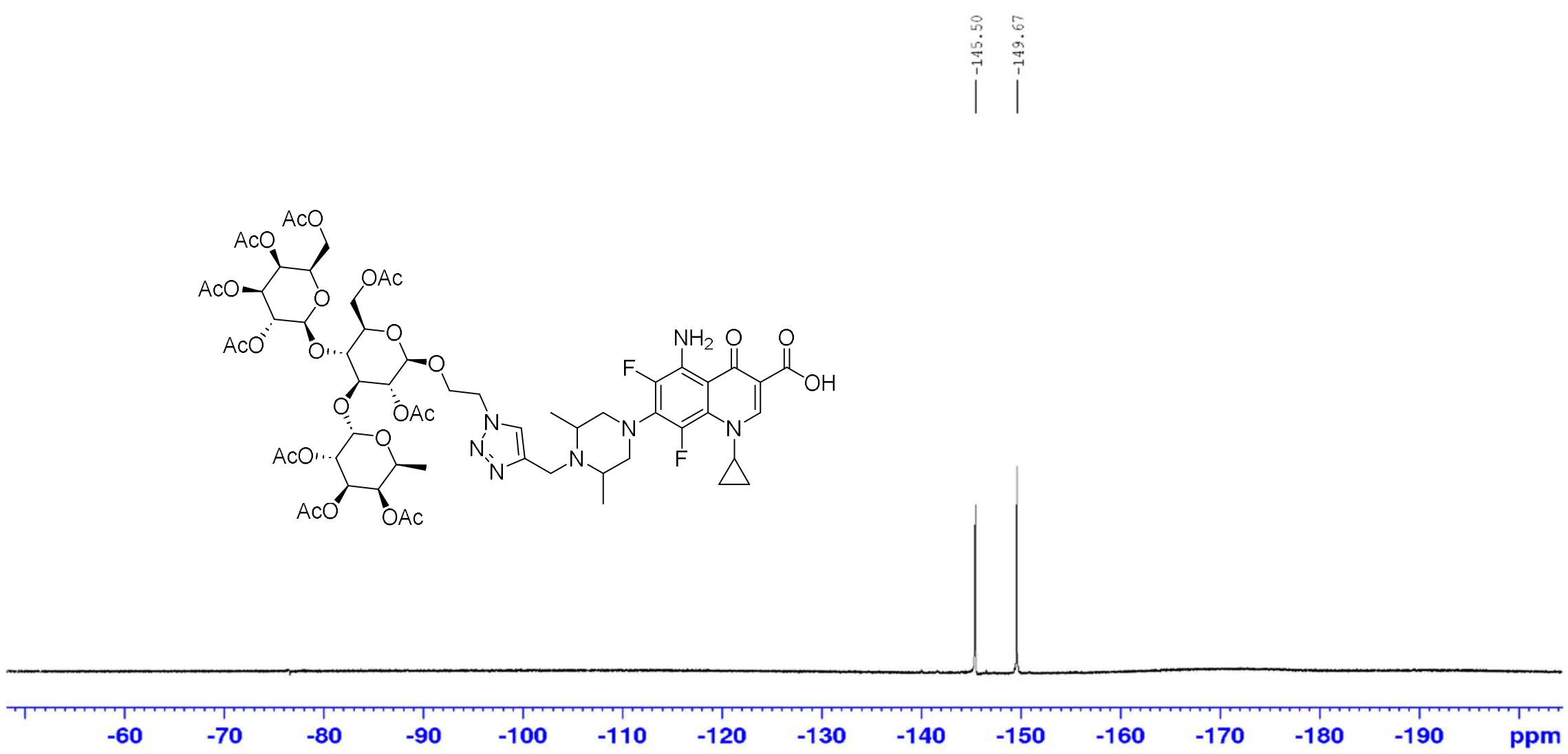

^{19}F NMR (376 MHz, CD_3OD) spectrum of compound **8d.**

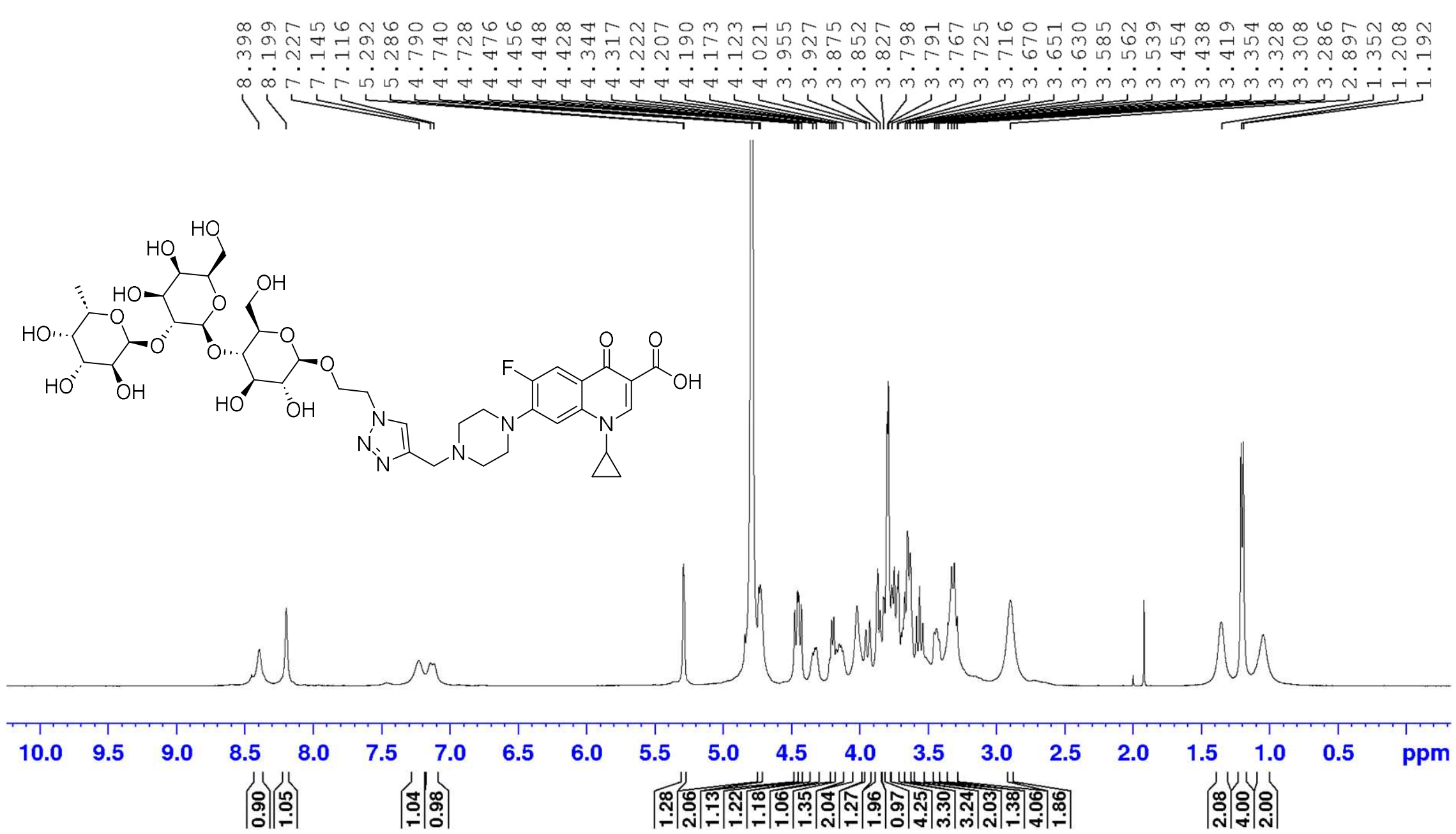

^{1}H NMR (400 MHz, D_2O) spectrum of compound **9a.**

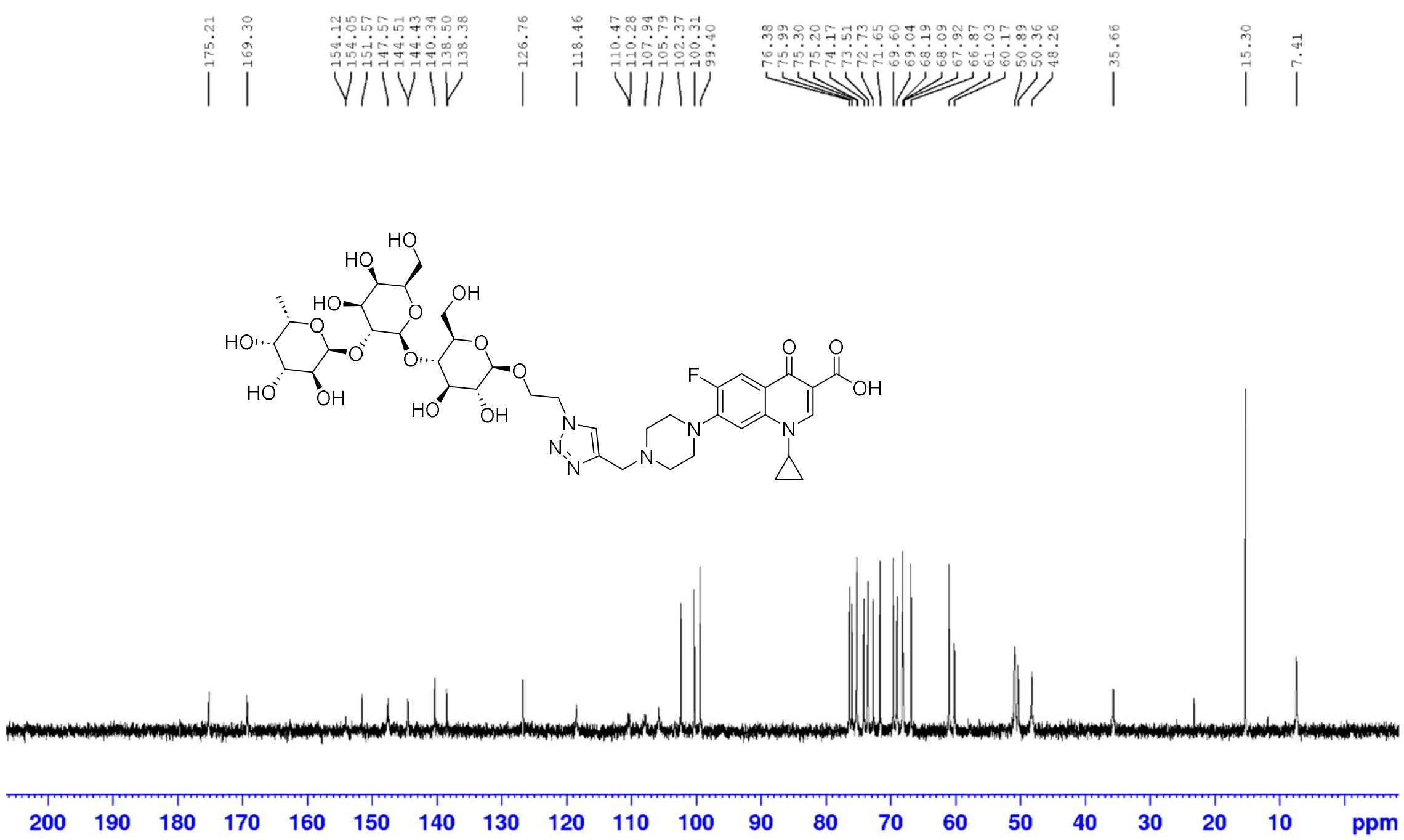

^{13}C NMR (100 MHz, D_2O) spectrum of compound **9a.**

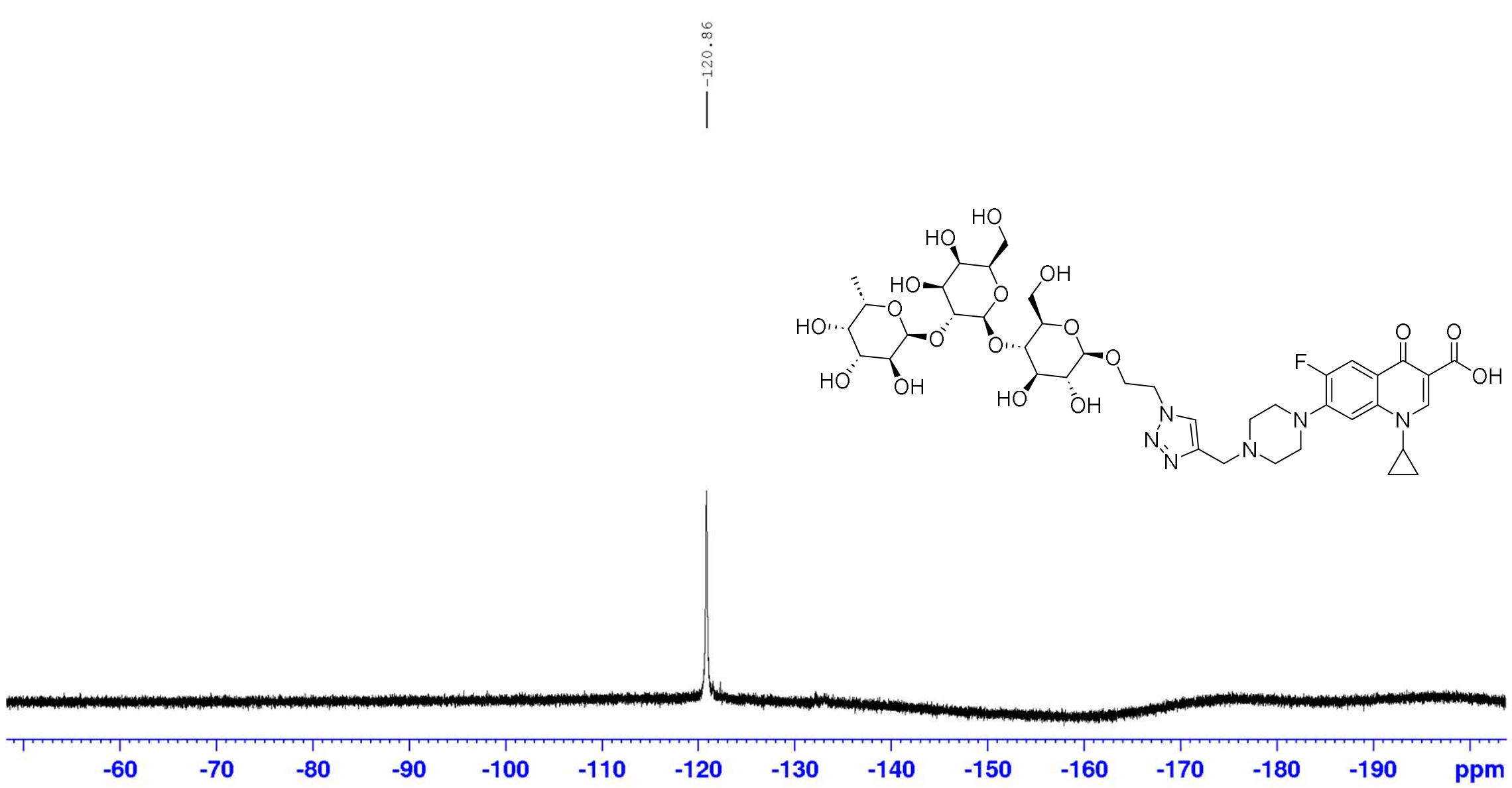

^{19}F NMR (376 MHz, D_2O) spectrum of compound **9a.**

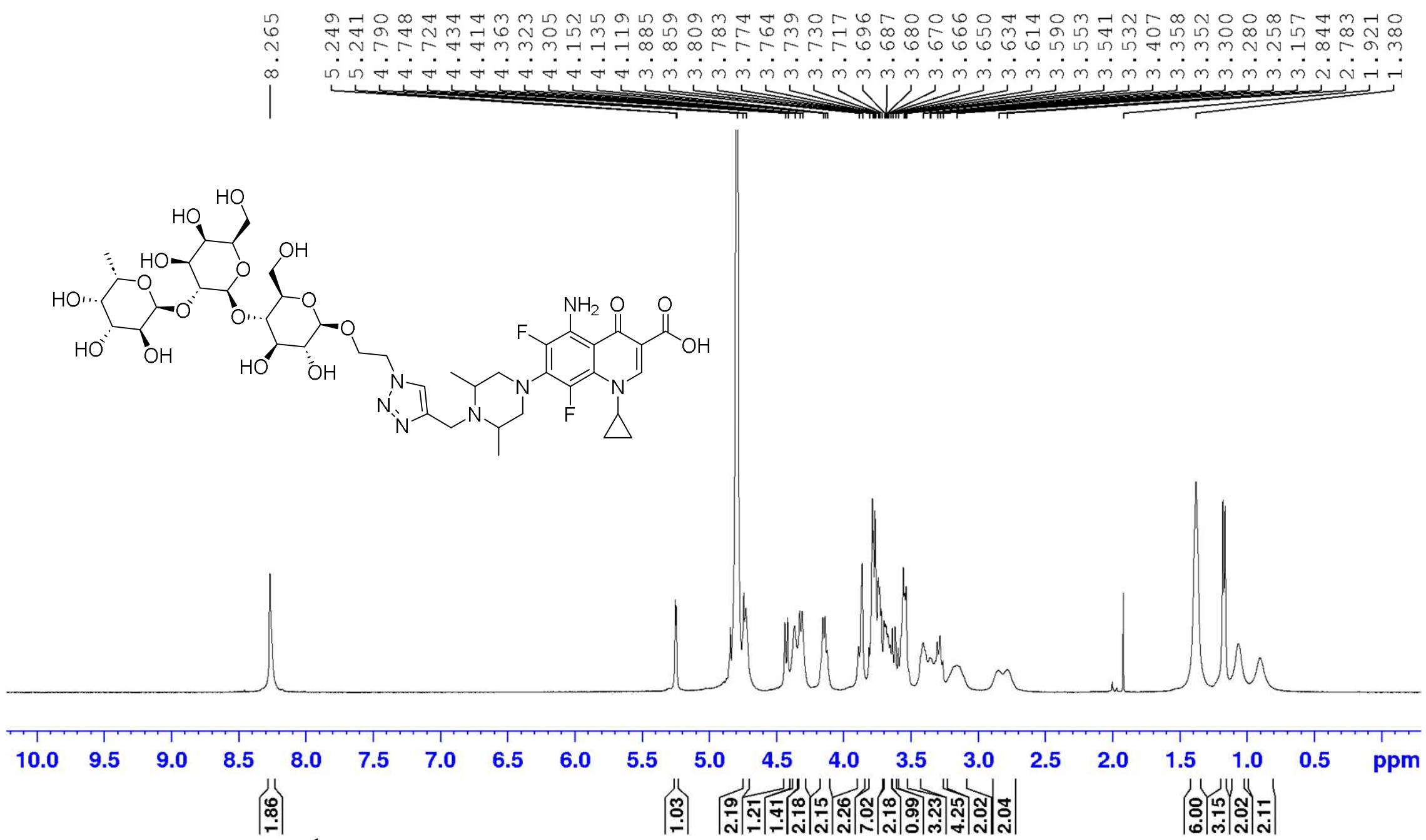

^{1}H NMR (400 MHz, D_2O) spectrum of compound **9b.**

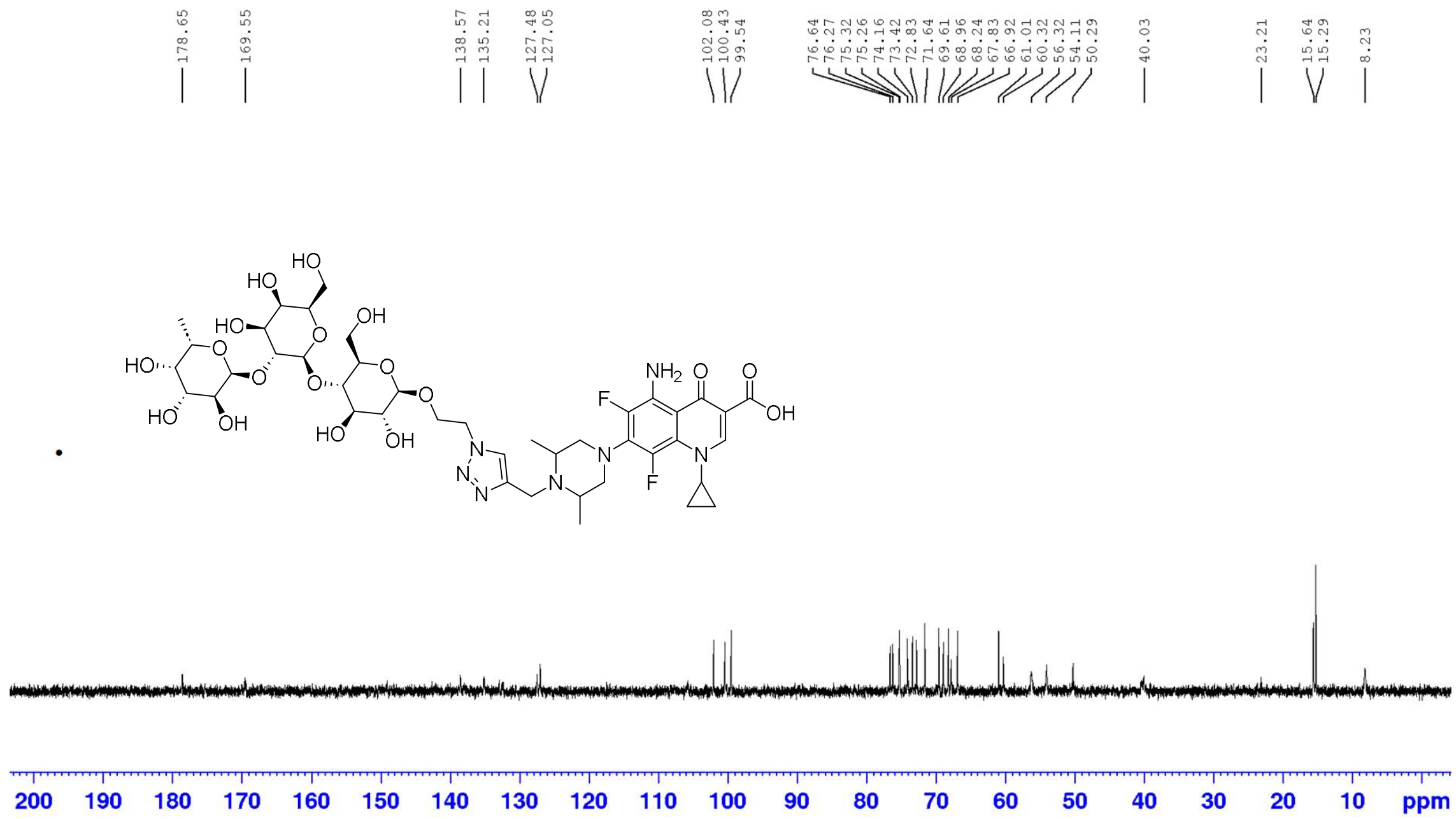

^{13}C NMR (100 MHz, D_2O) spectrum of compound **9b.**

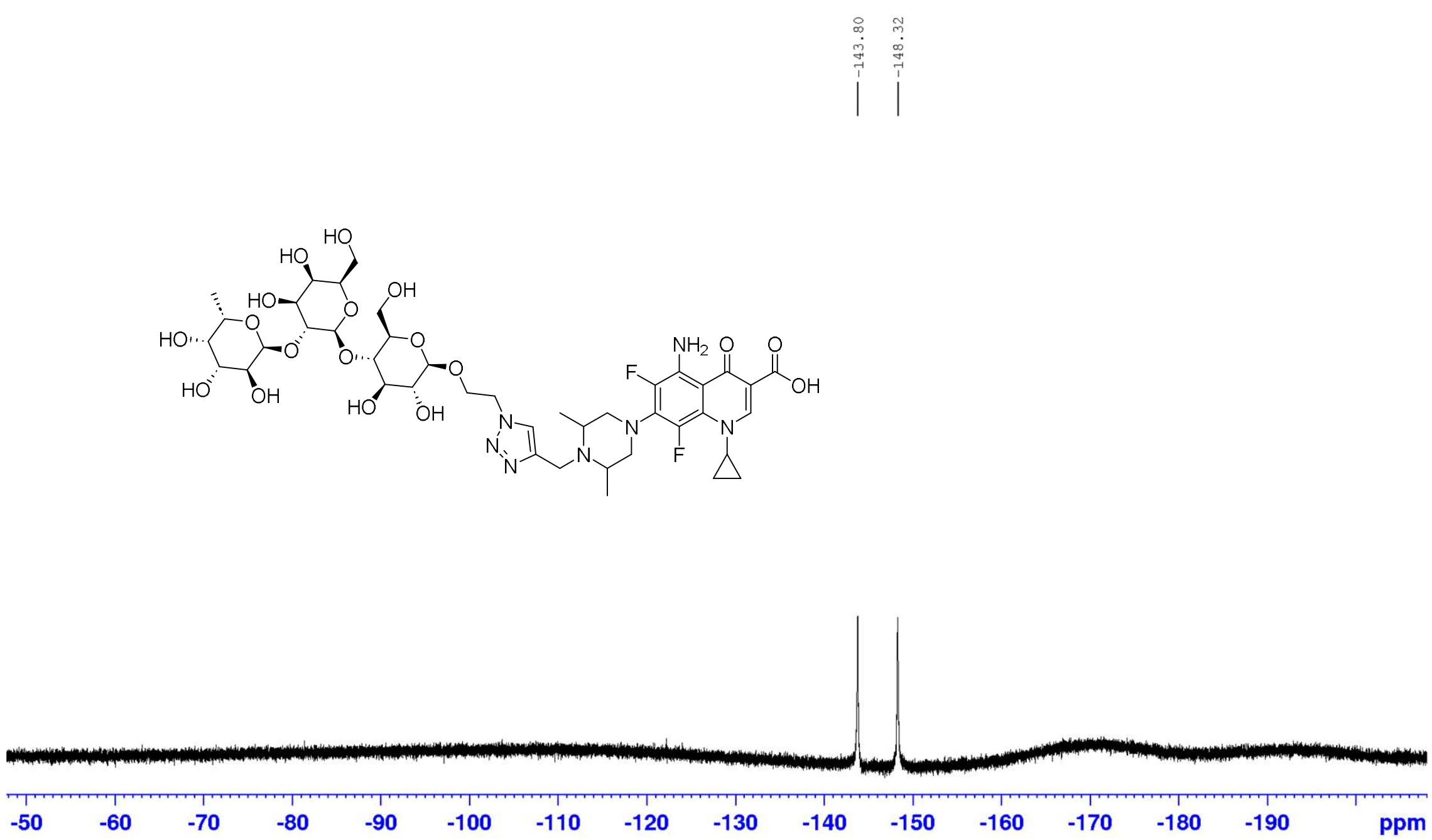

^{19}F NMR (376 MHz, D_2O) spectrum of compound **9b.**

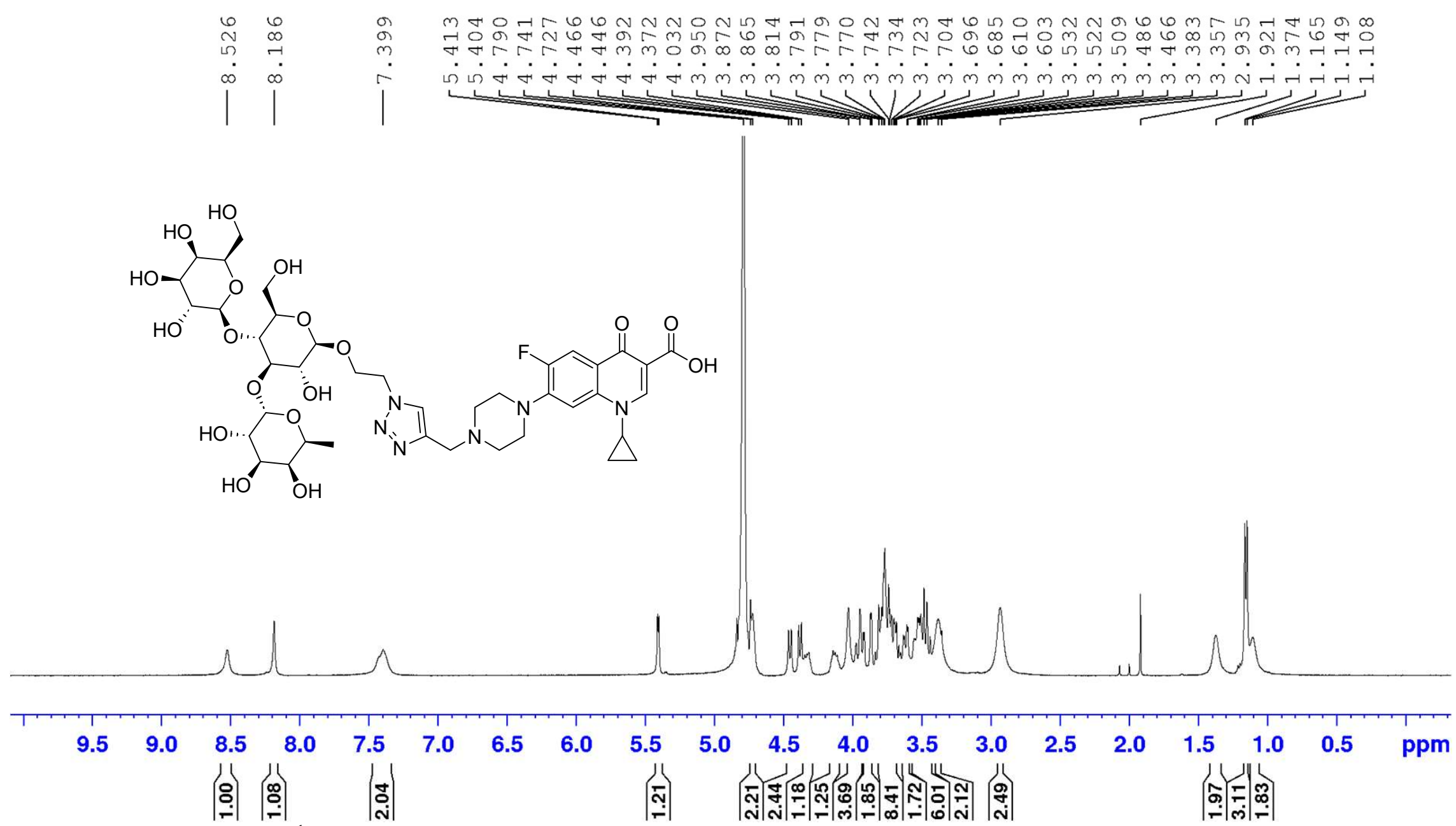

^{1}H NMR (400 MHz, D_2O) spectrum of compound **9c.**

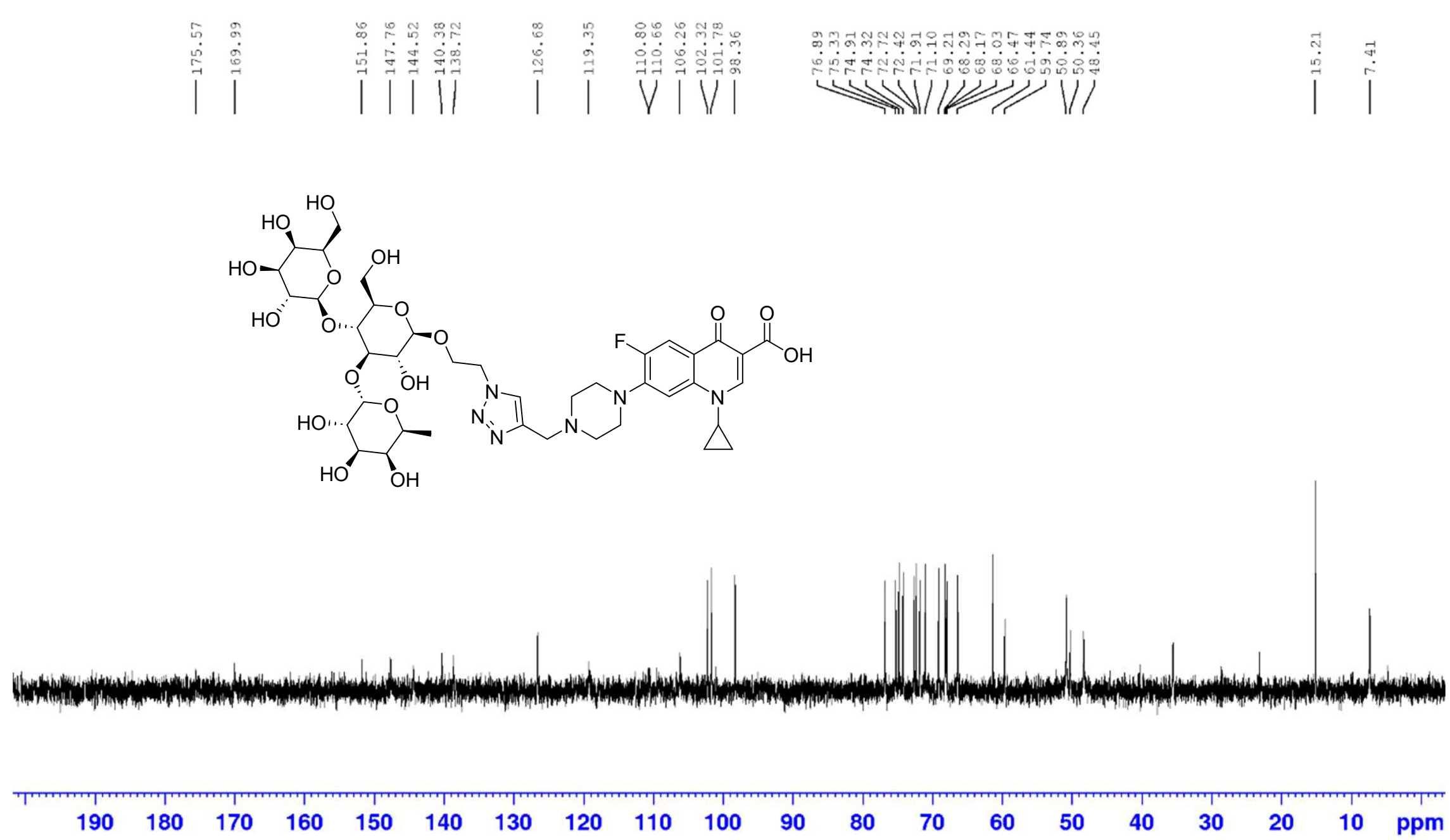

^{13}C NMR (100 MHz, D_2O) spectrum of compound **9c.**

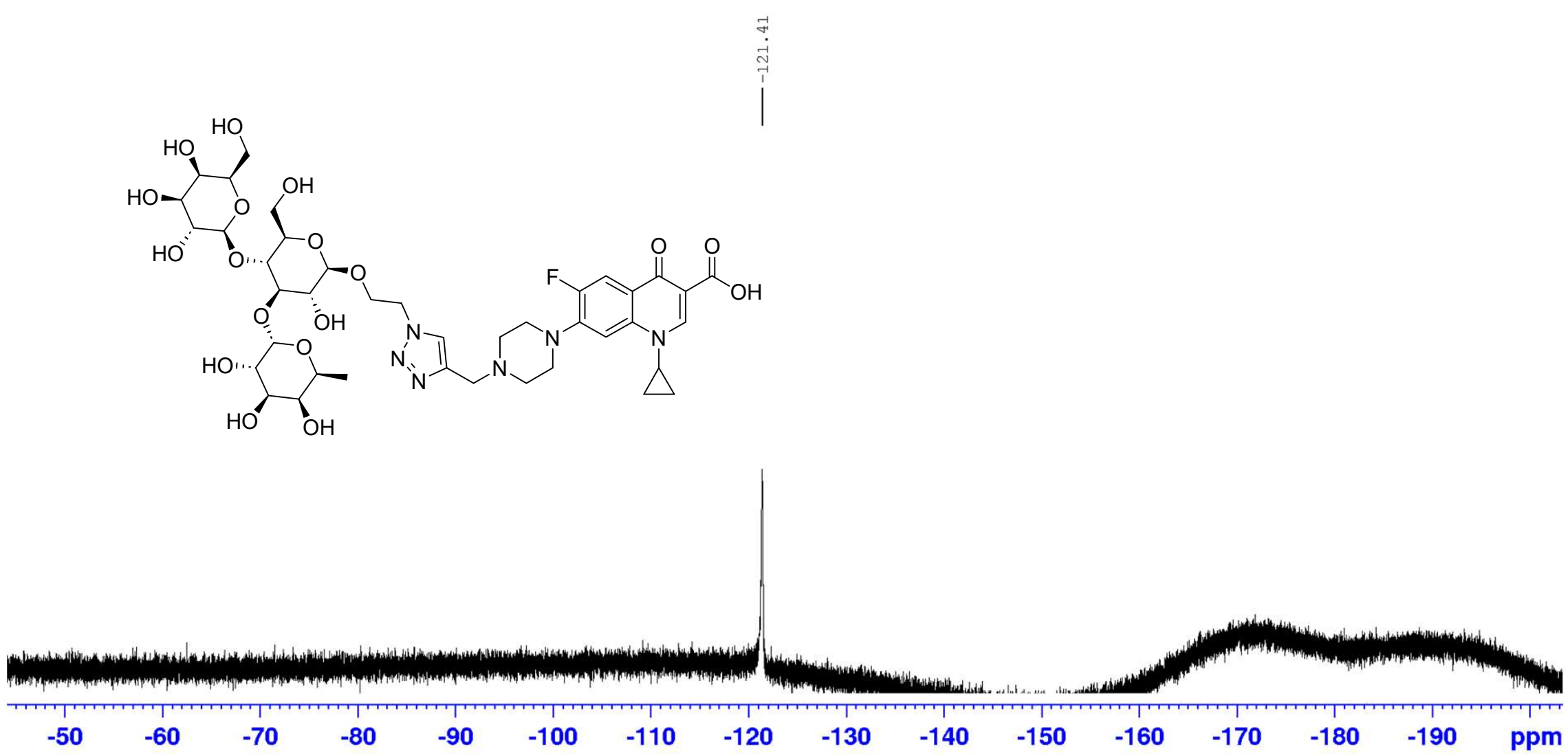

^{19}F NMR (376 MHz, D_2O) spectrum of compound **9c.**

^{1}H NMR (400 MHz, $(CD_3)_2SO$) spectrum of compound **9d.**

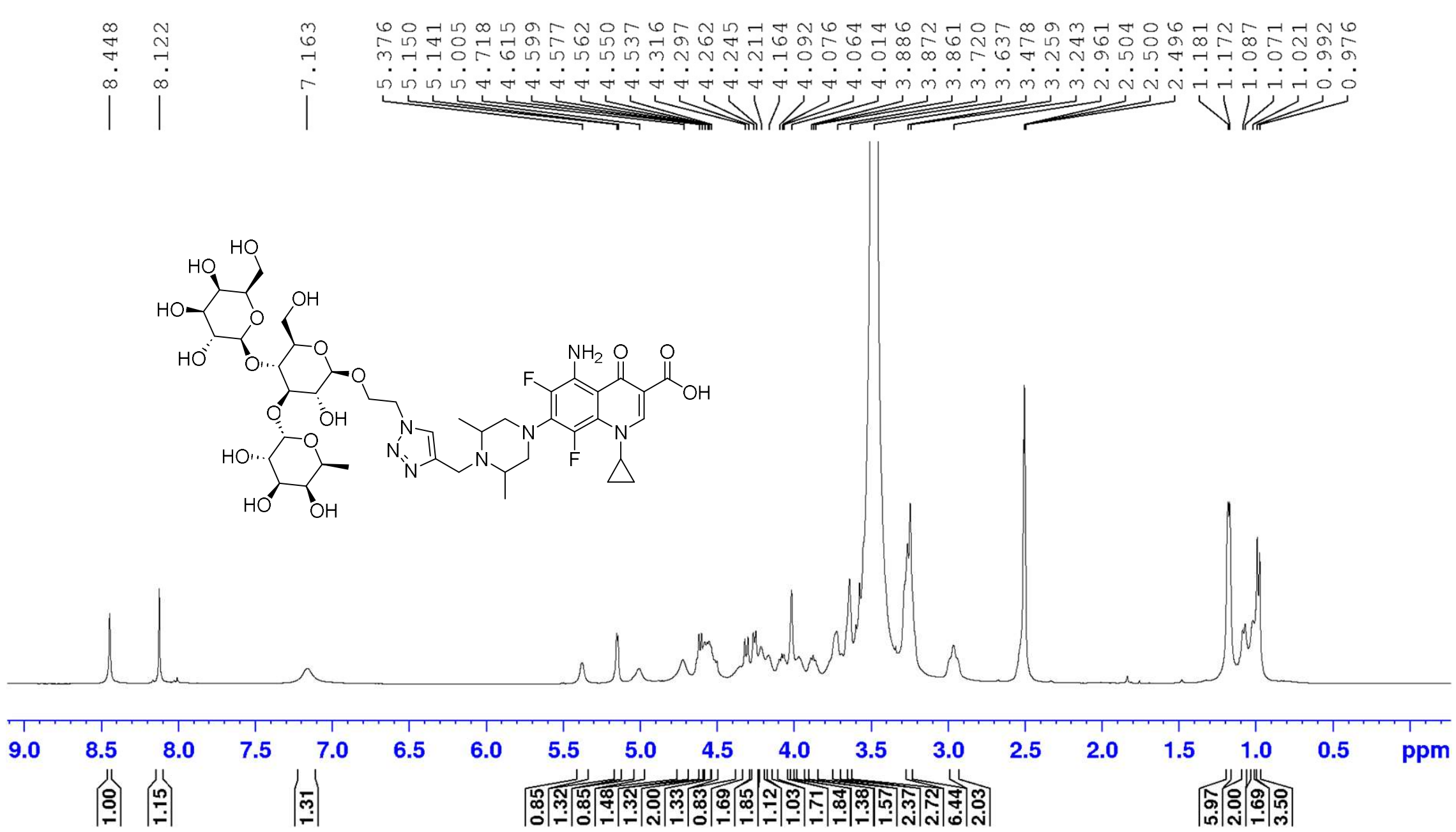

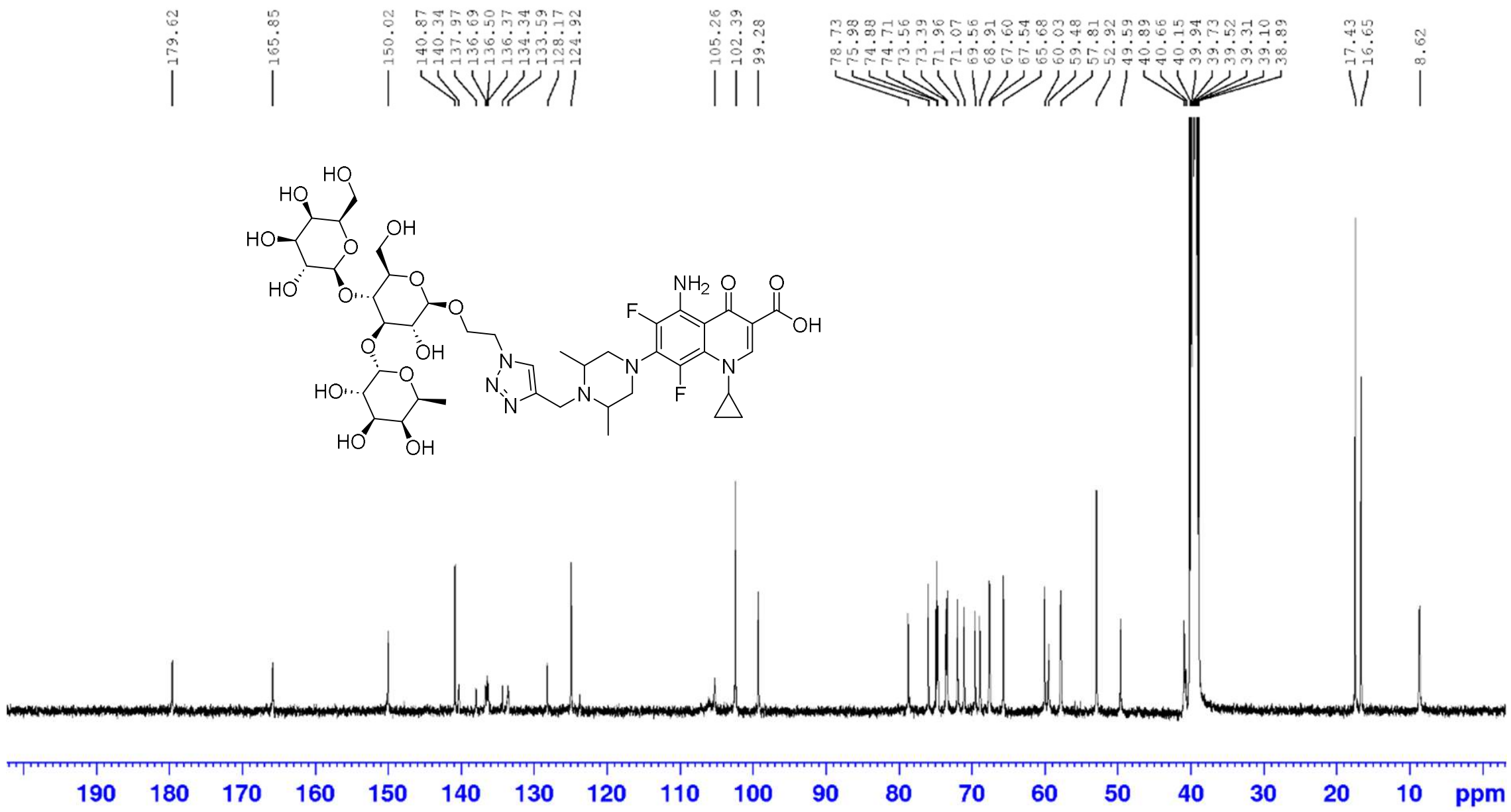

^{13}C NMR (100 MHz, $(CD_3)_2SO$) spectrum of compound **9d.**

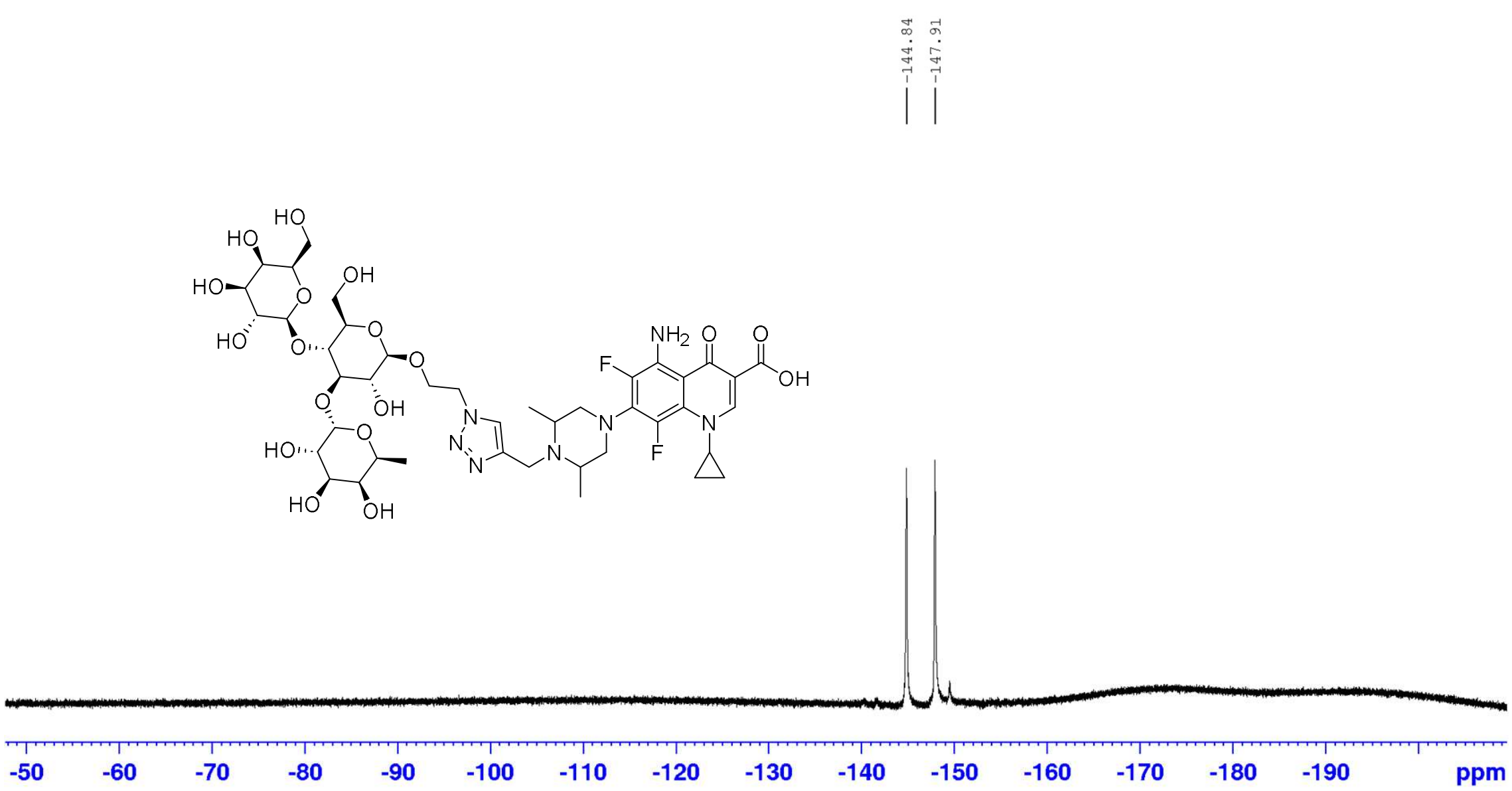

^{19}F NMR (376 MHz, $(CD_3)_2SO$) spectrum of compound **9d.**

^{1}H NMR (400 MHz, CDCl$_3$) spectrum of compound **10a.**

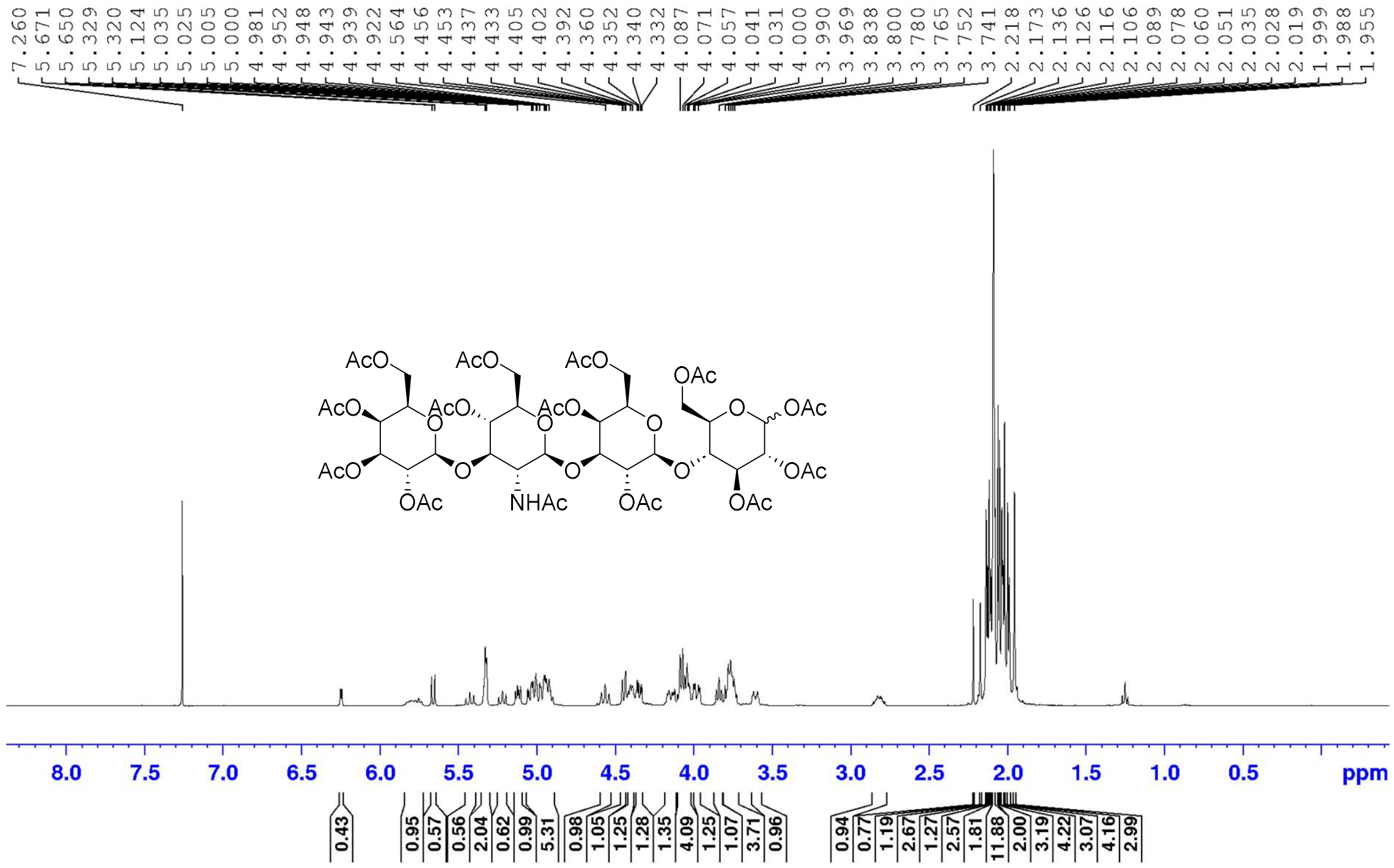

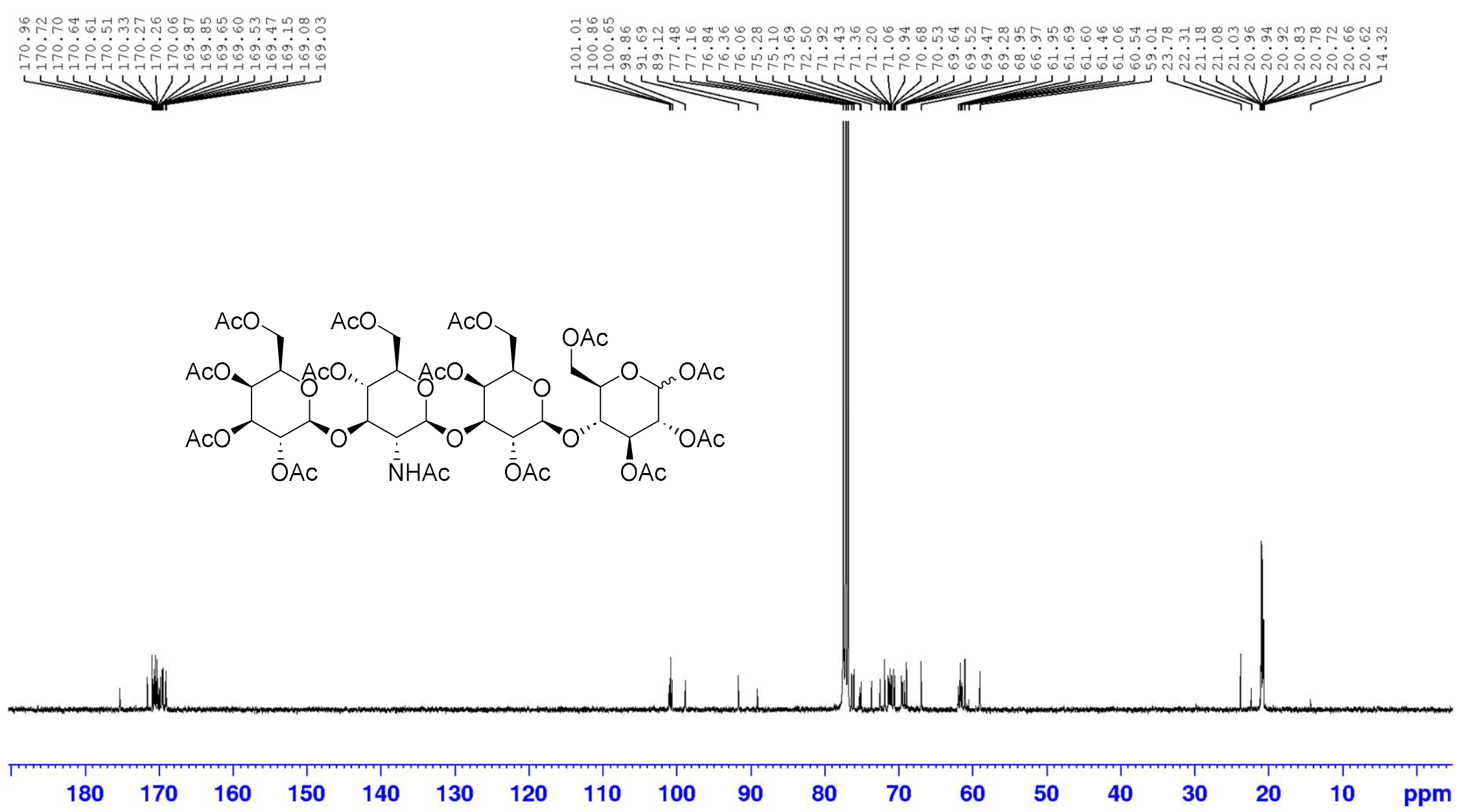

^{13}C NMR (100 MHz, CDCl$_3$) spectrum of compound **10a.**

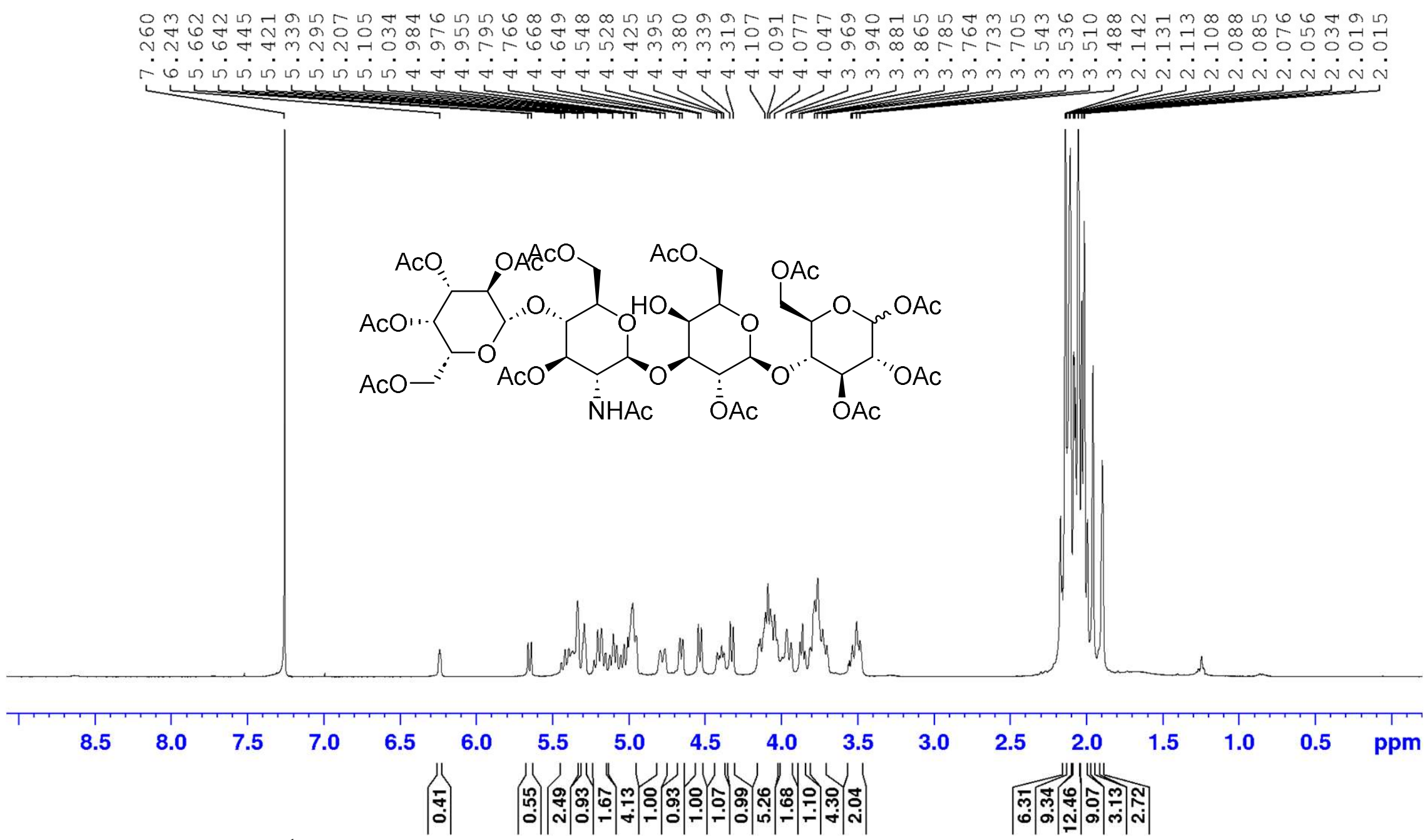

^{1}H NMR (400 MHz, $CDCl_3$) spectrum of compound **10b.**

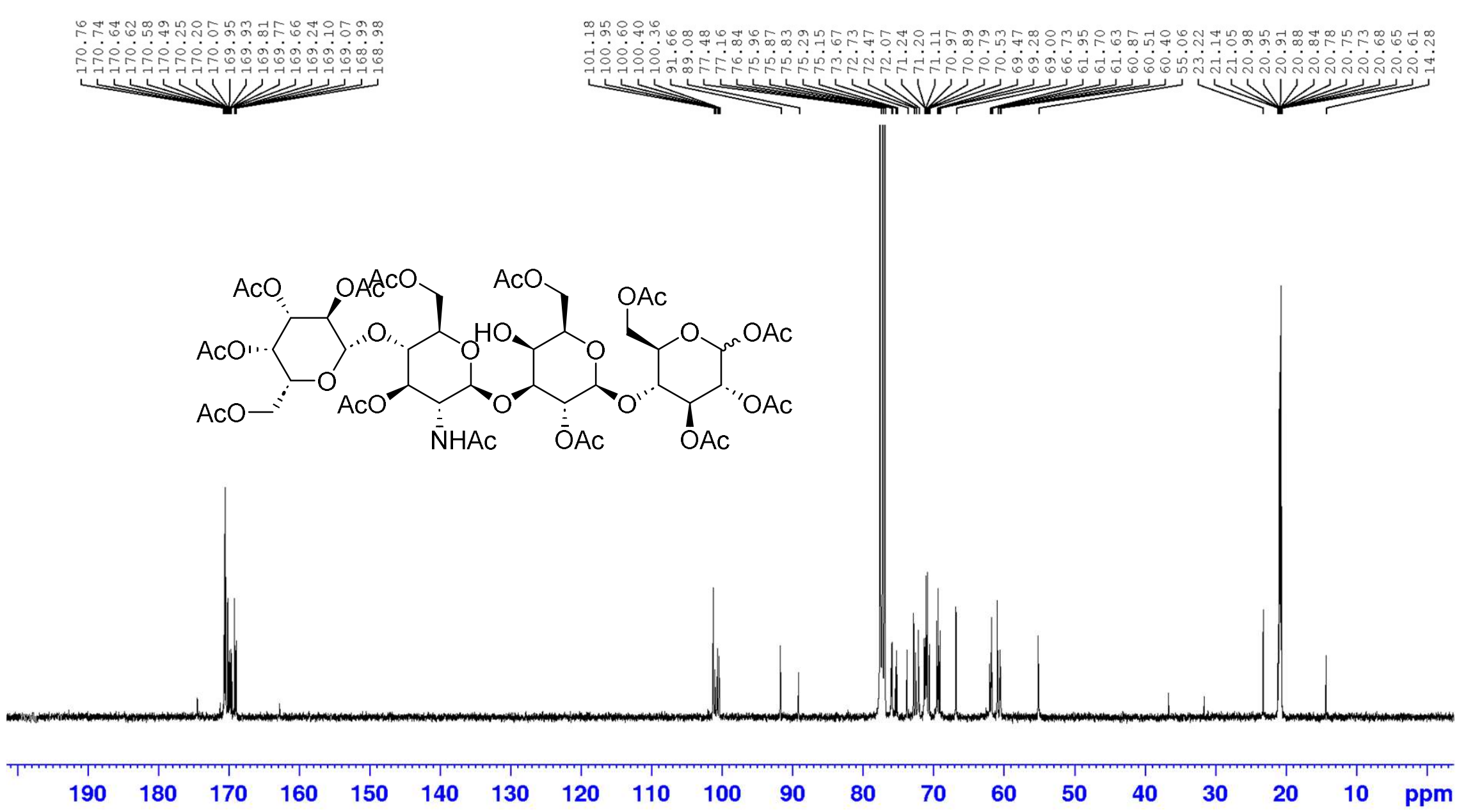

^{13}C NMR (100 MHz, $CDCl_3$) spectrum of compound **10b.**

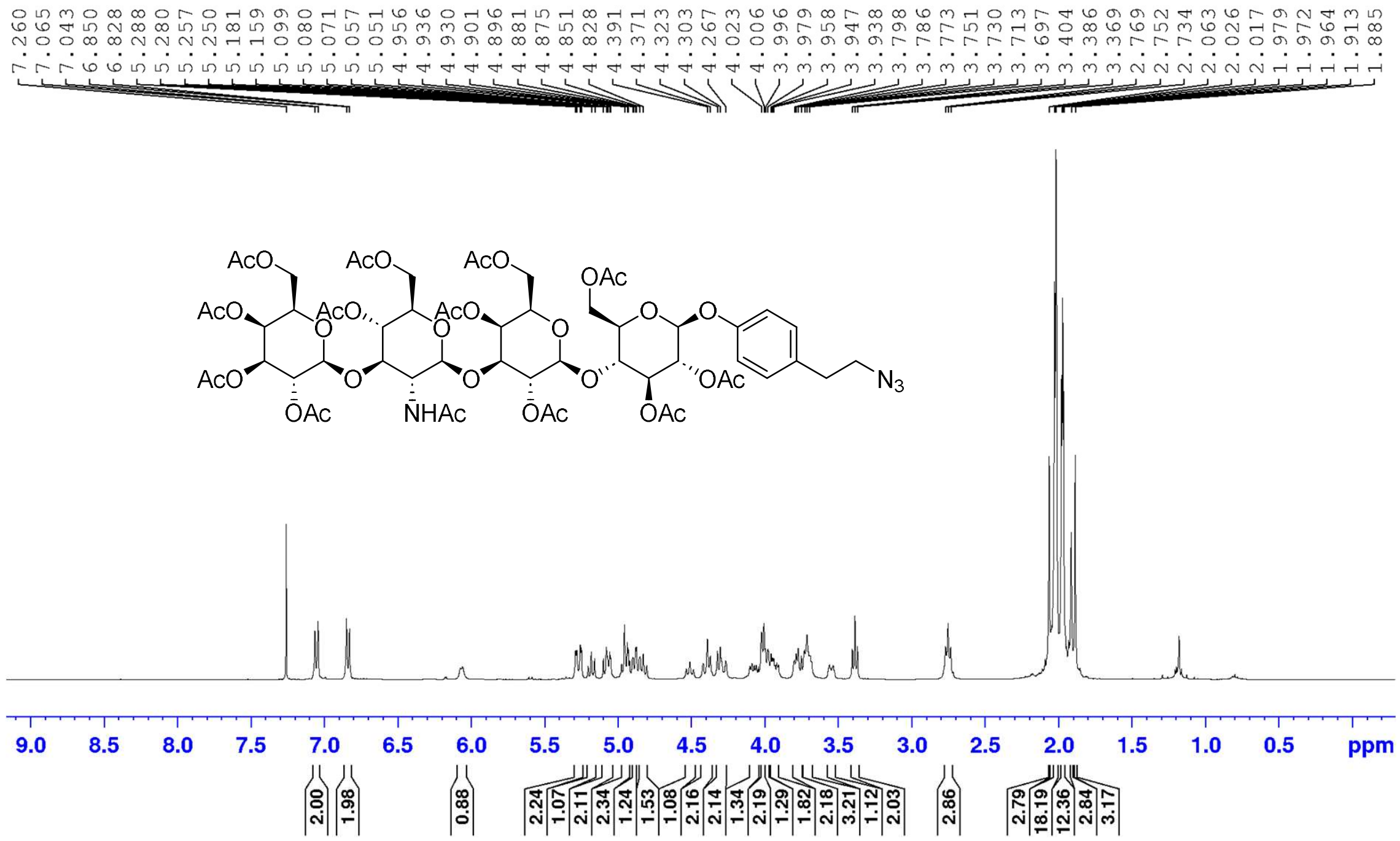

^{1}H NMR (400 MHz, $CDCl_3$) spectrum of compound **11a.**

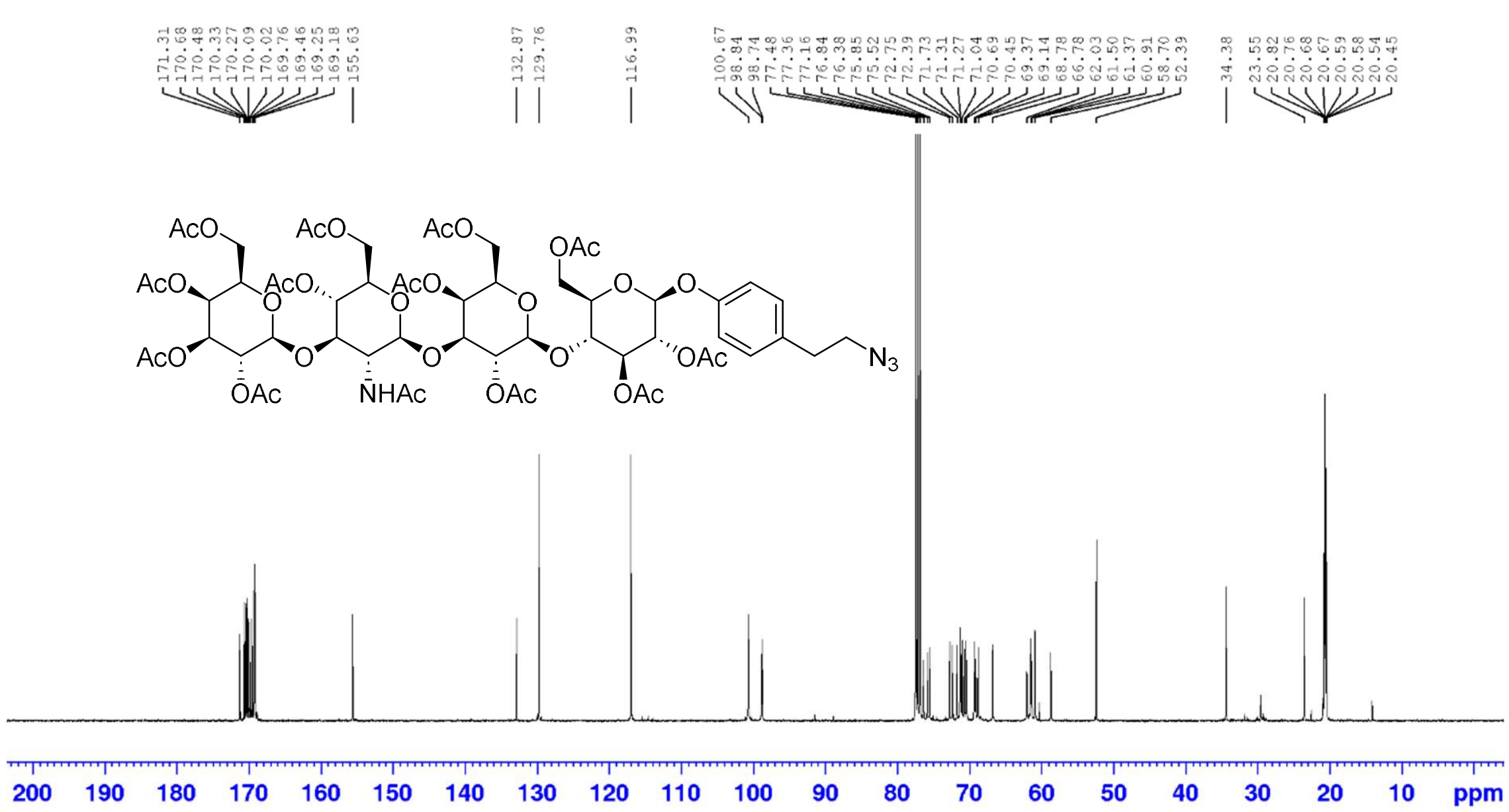

^{13}C NMR (100 MHz, $CDCl_3$) spectrum of compound **11a.**

^{1}H NMR (400 MHz, $CDCl_3$) spectrum of compound **11b.**

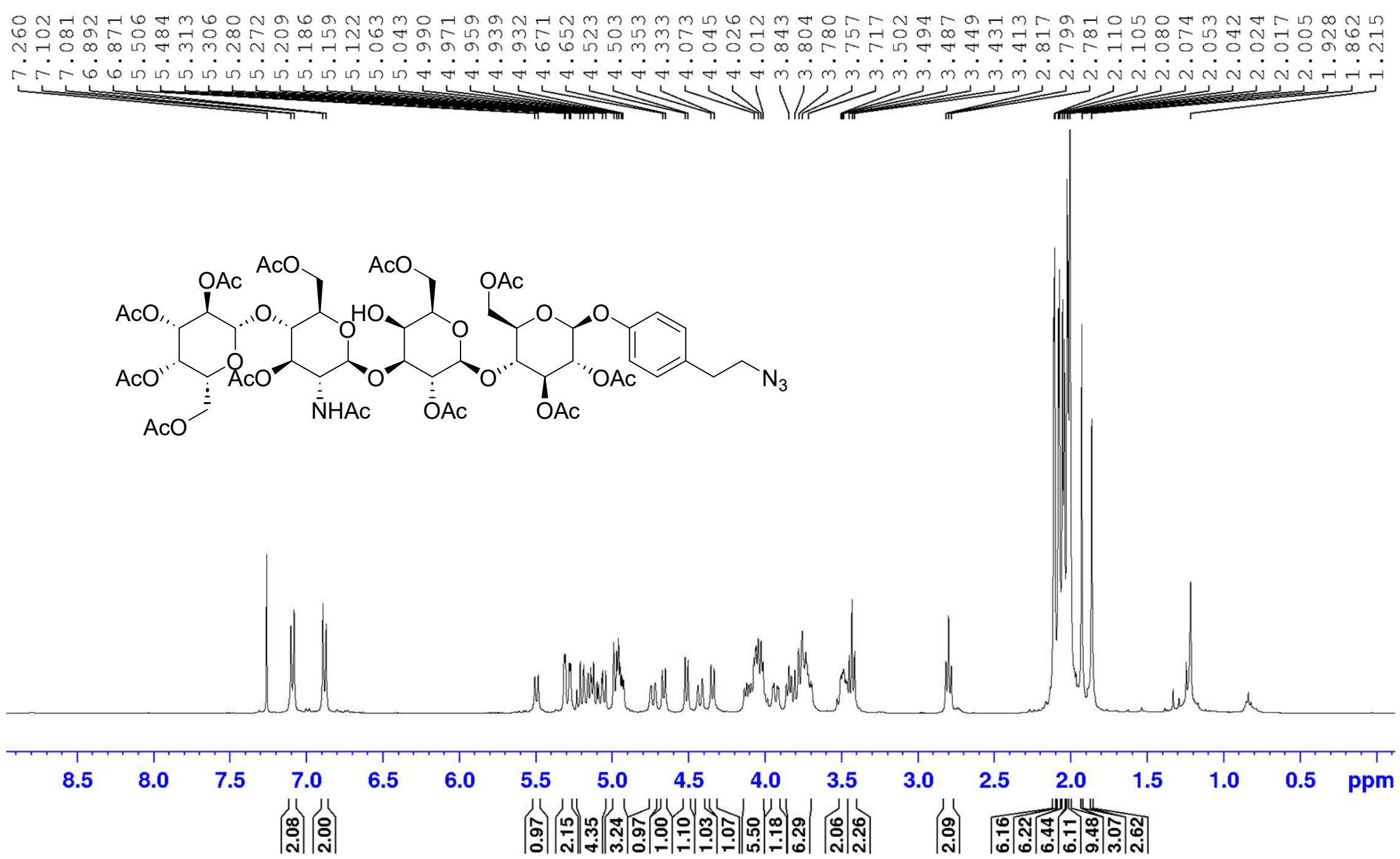

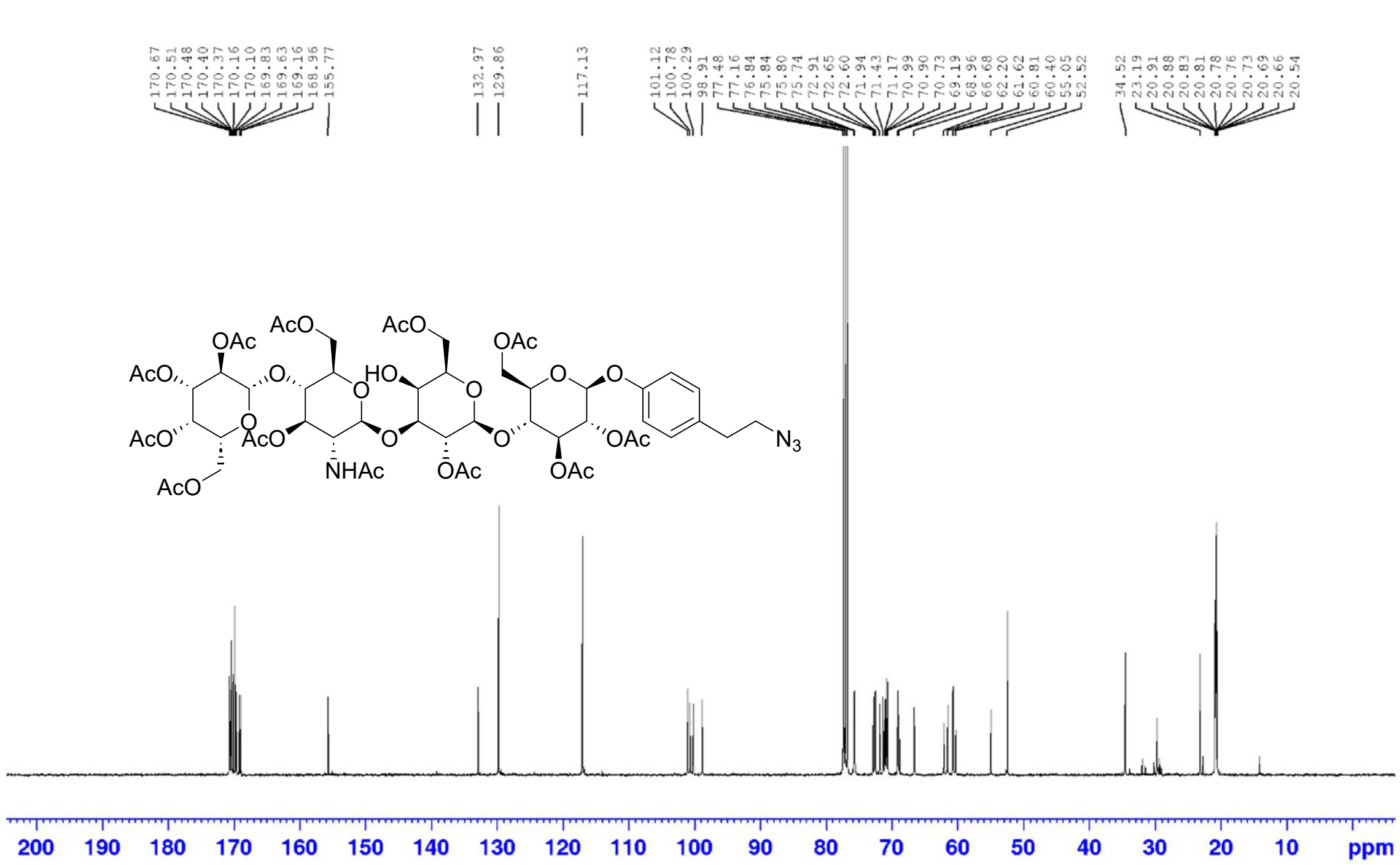

^{13}C NMR (100 MHz, $CDCl_3$) spectrum of compound **11b.**

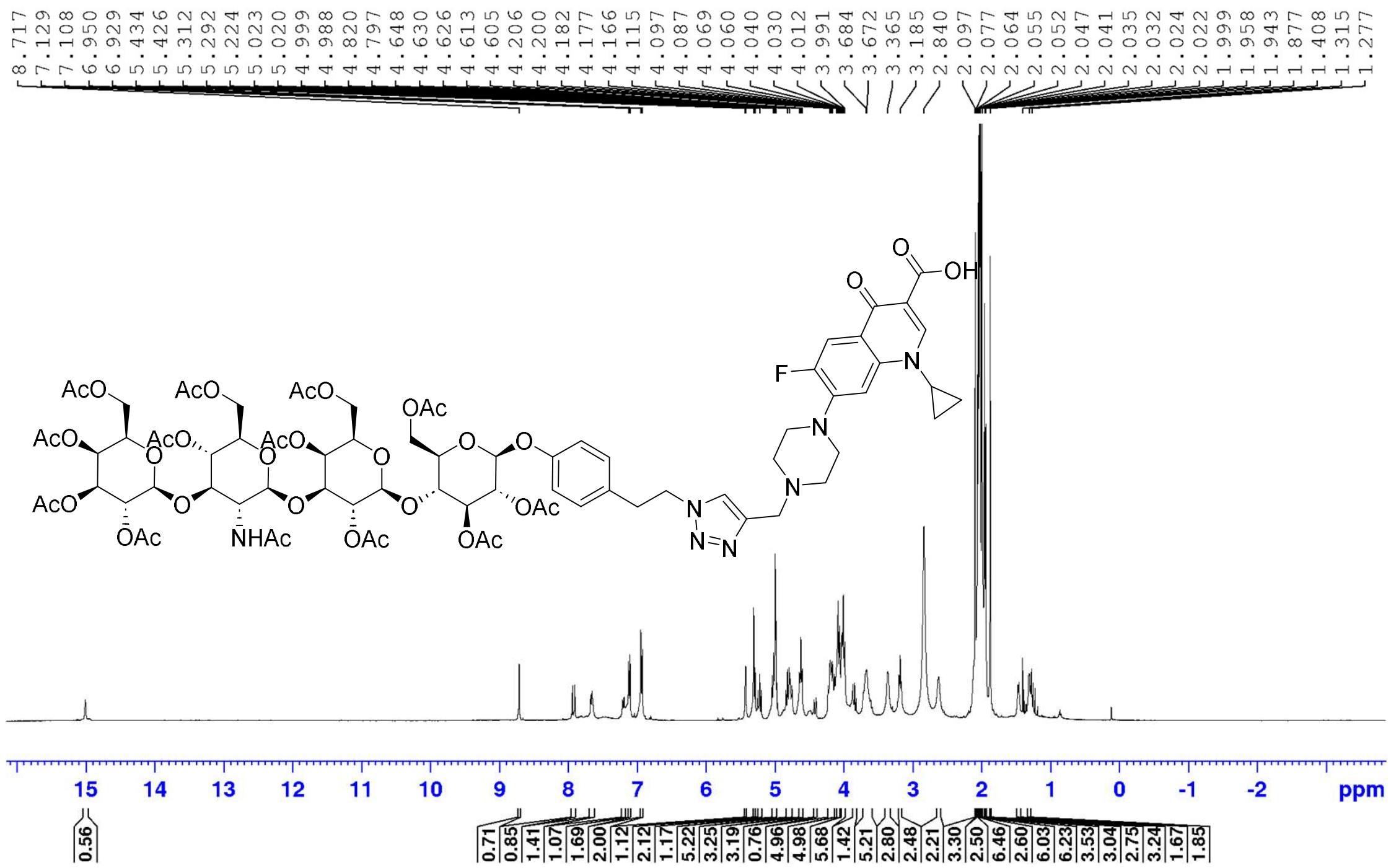

^{1}H NMR (400 MHz, CD_3OD) spectrum of compound **12a.**

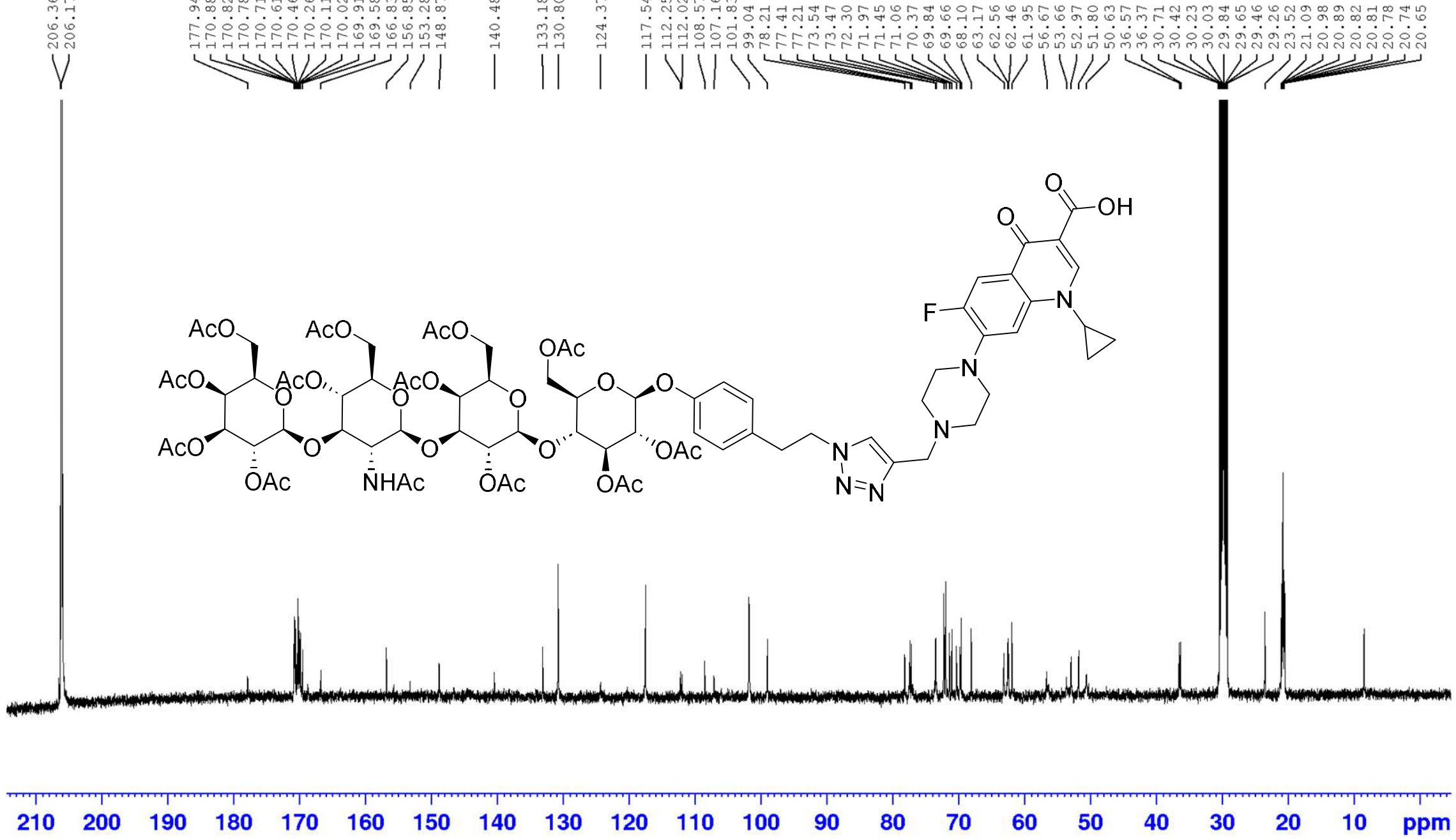

^{13}C NMR (100 MHz, CD_3OD) spectrum of compound **12a.**

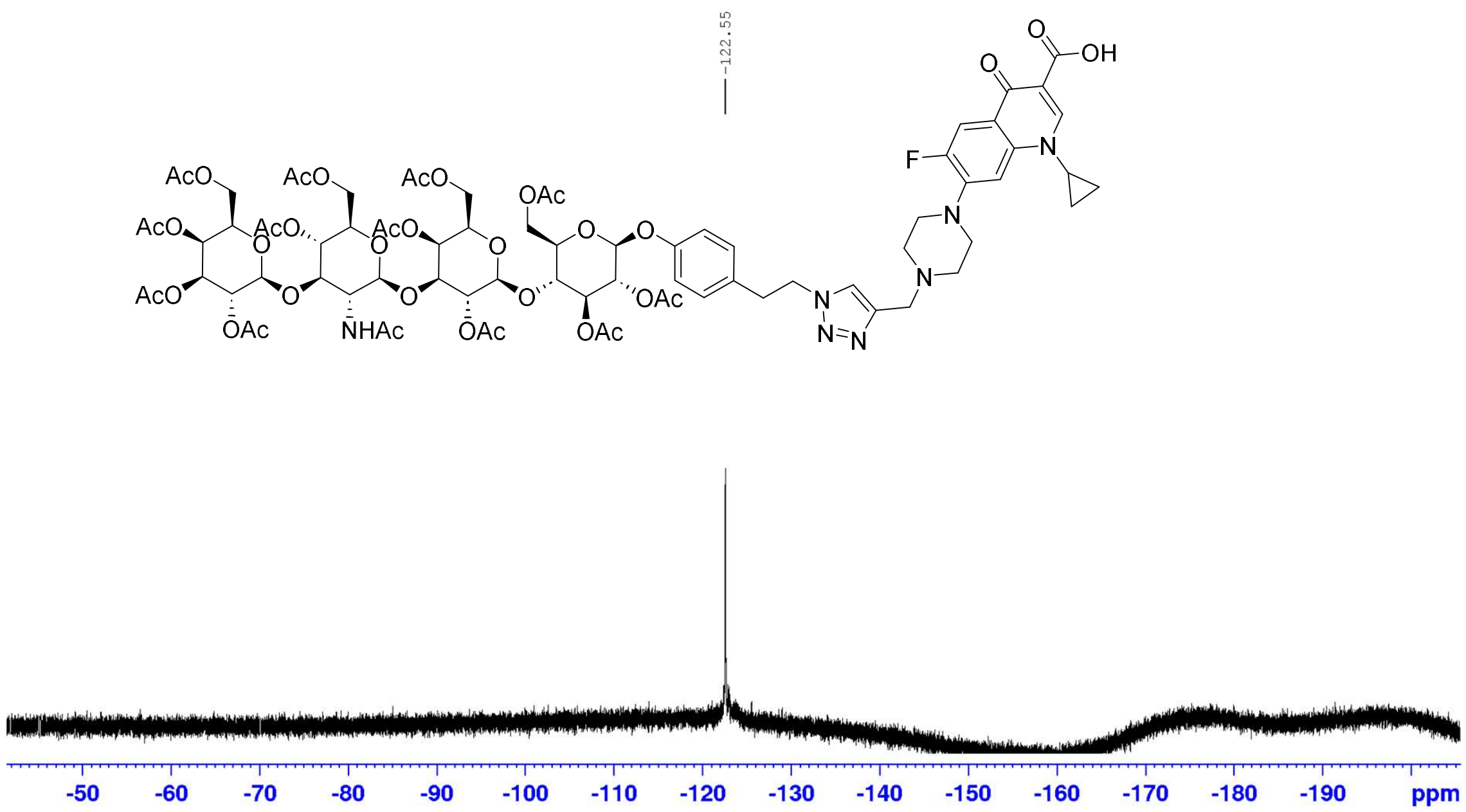

^{19}F NMR (376 MHz, CD_3OD) spectrum of compound **12a.**

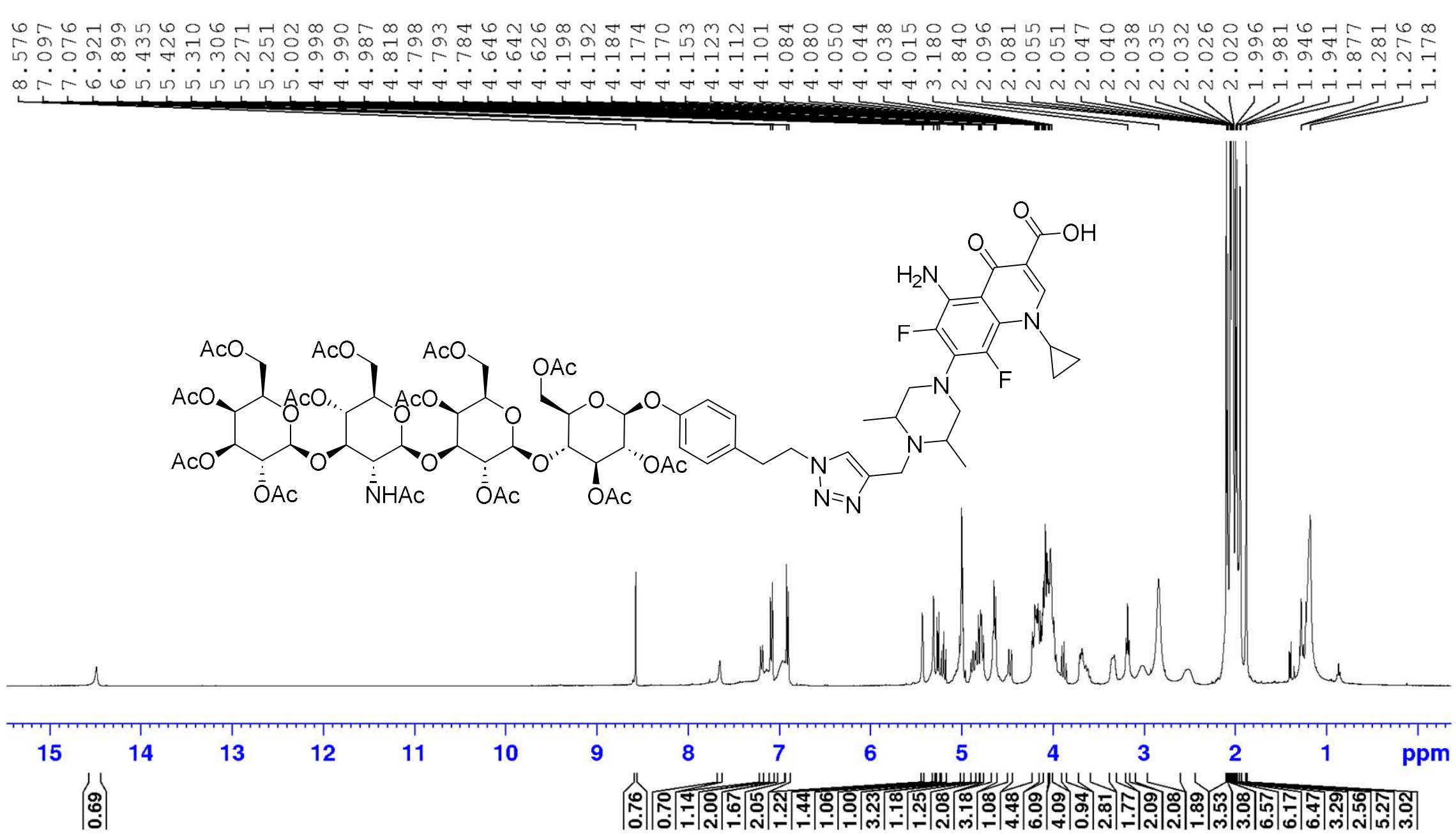

^{1}H NMR (400 MHz, CD_3OD) spectrum of compound **12b.**

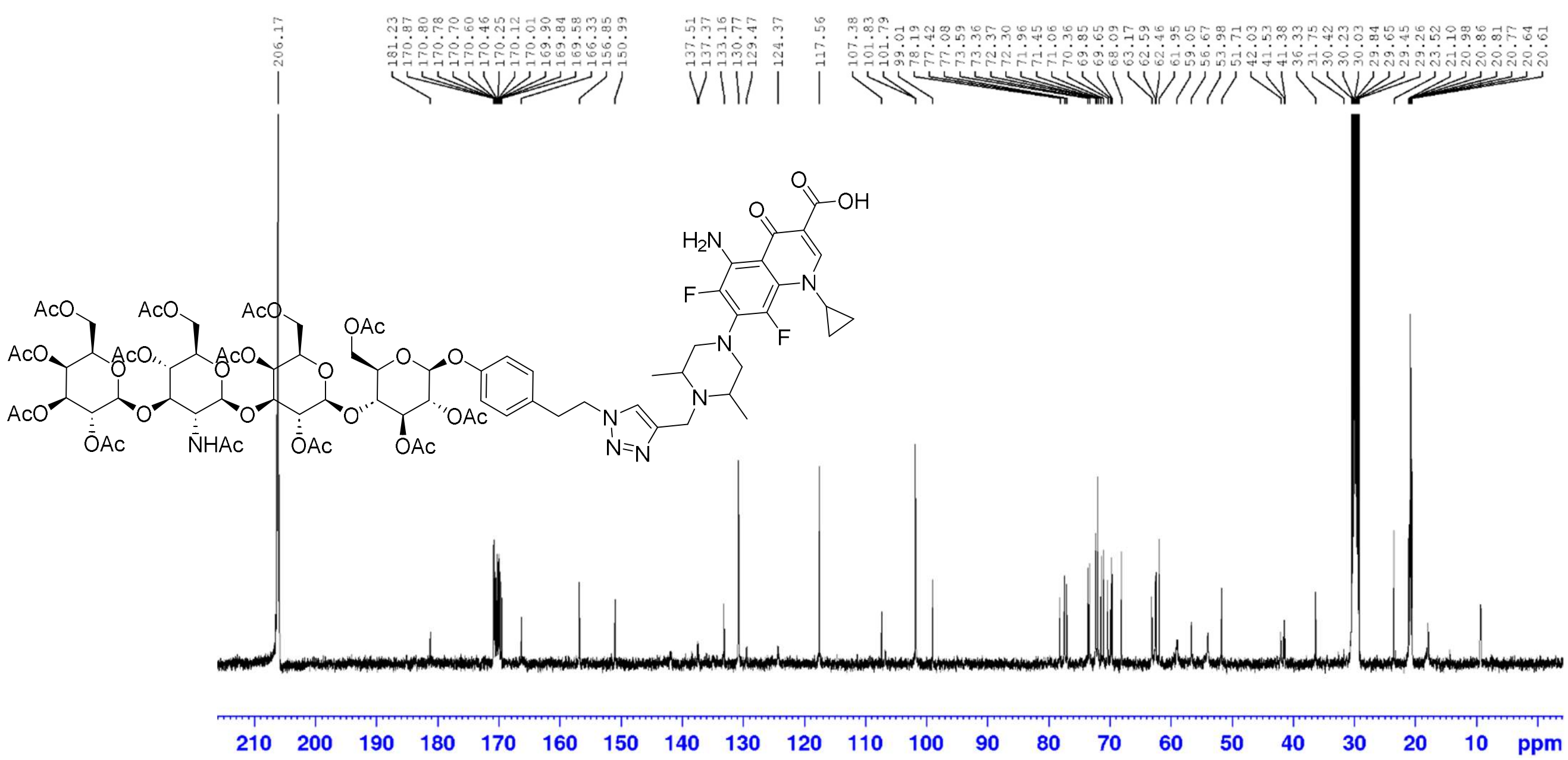

^{13}C NMR (100 MHz, CD_3OD) spectrum of compound **12b.**

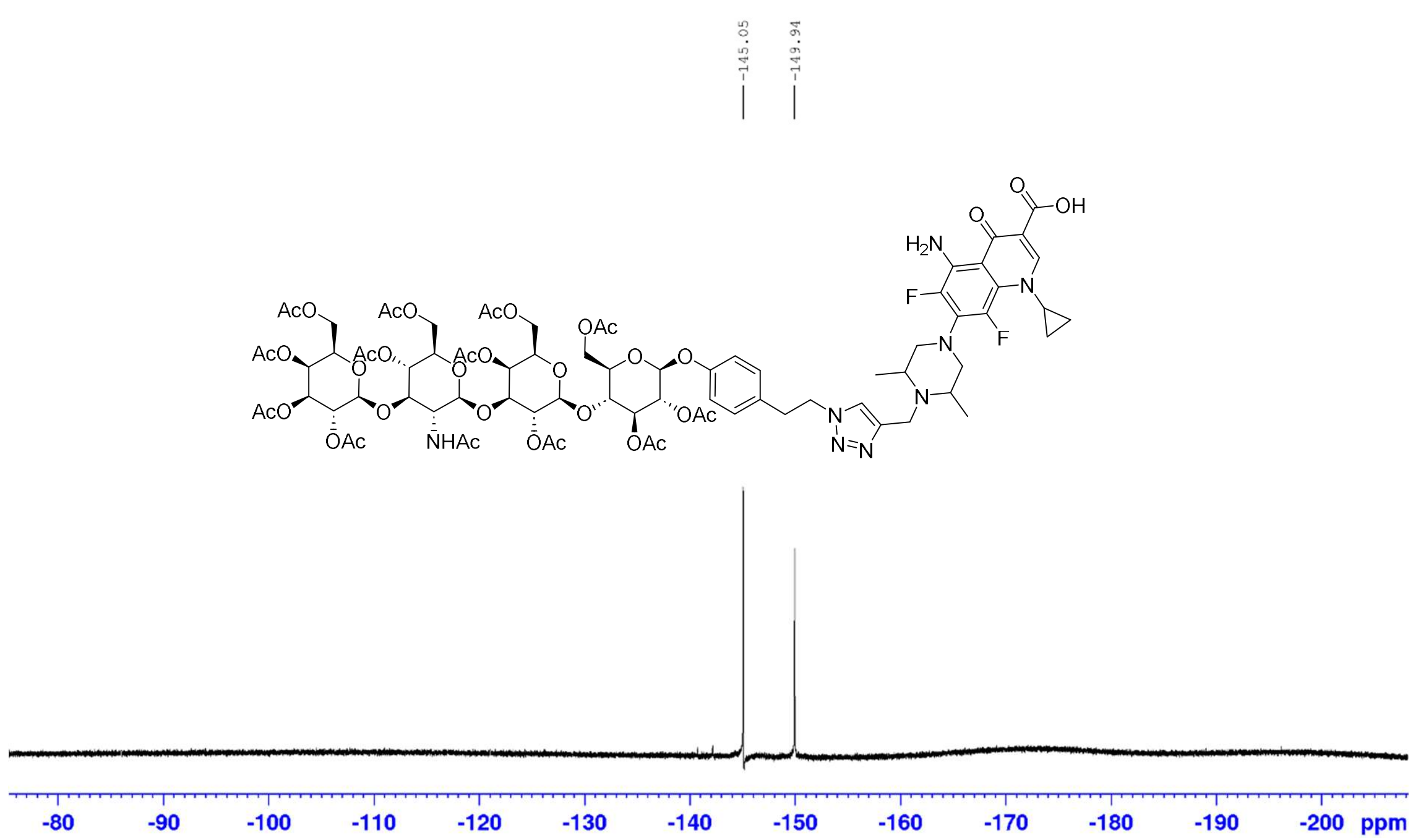

^{19}F NMR (376 MHz, CD_3OD) spectrum of compound **12b.**

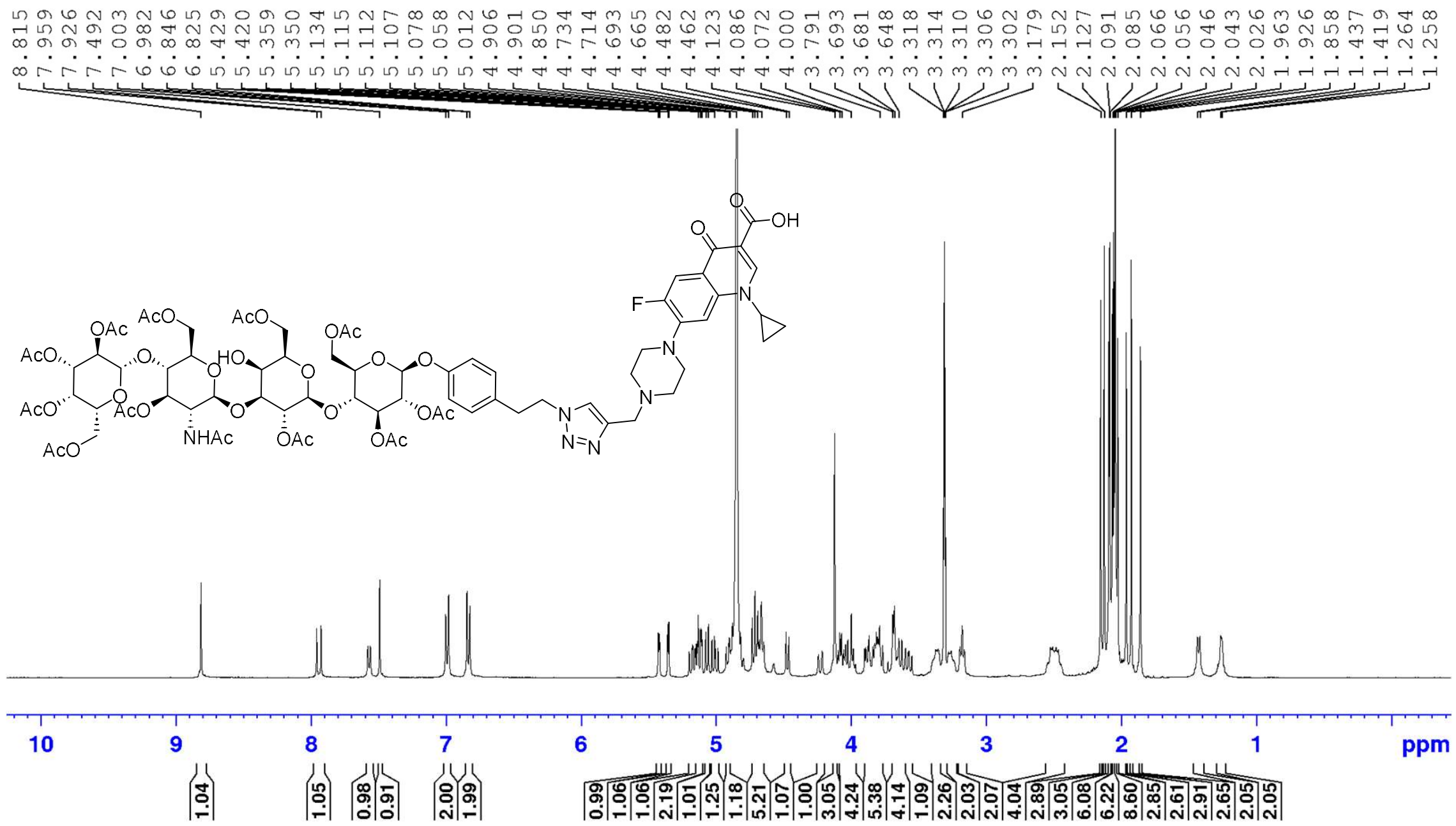

^{1}H NMR (400 MHz, CD_3OD) spectrum of compound **12c.**

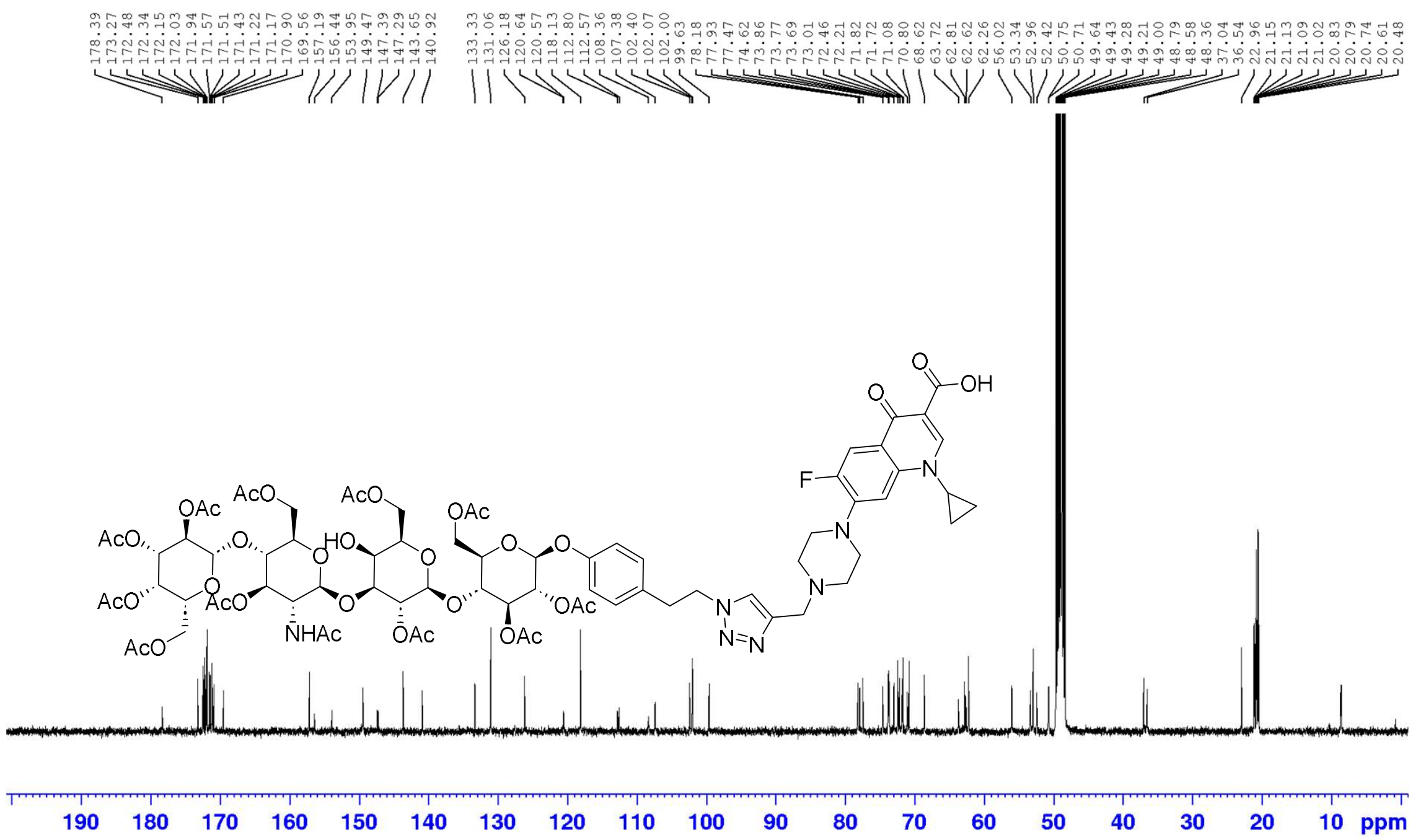

^{13}C NMR (100 MHz, CD_3OD) spectrum of compound **12c.**

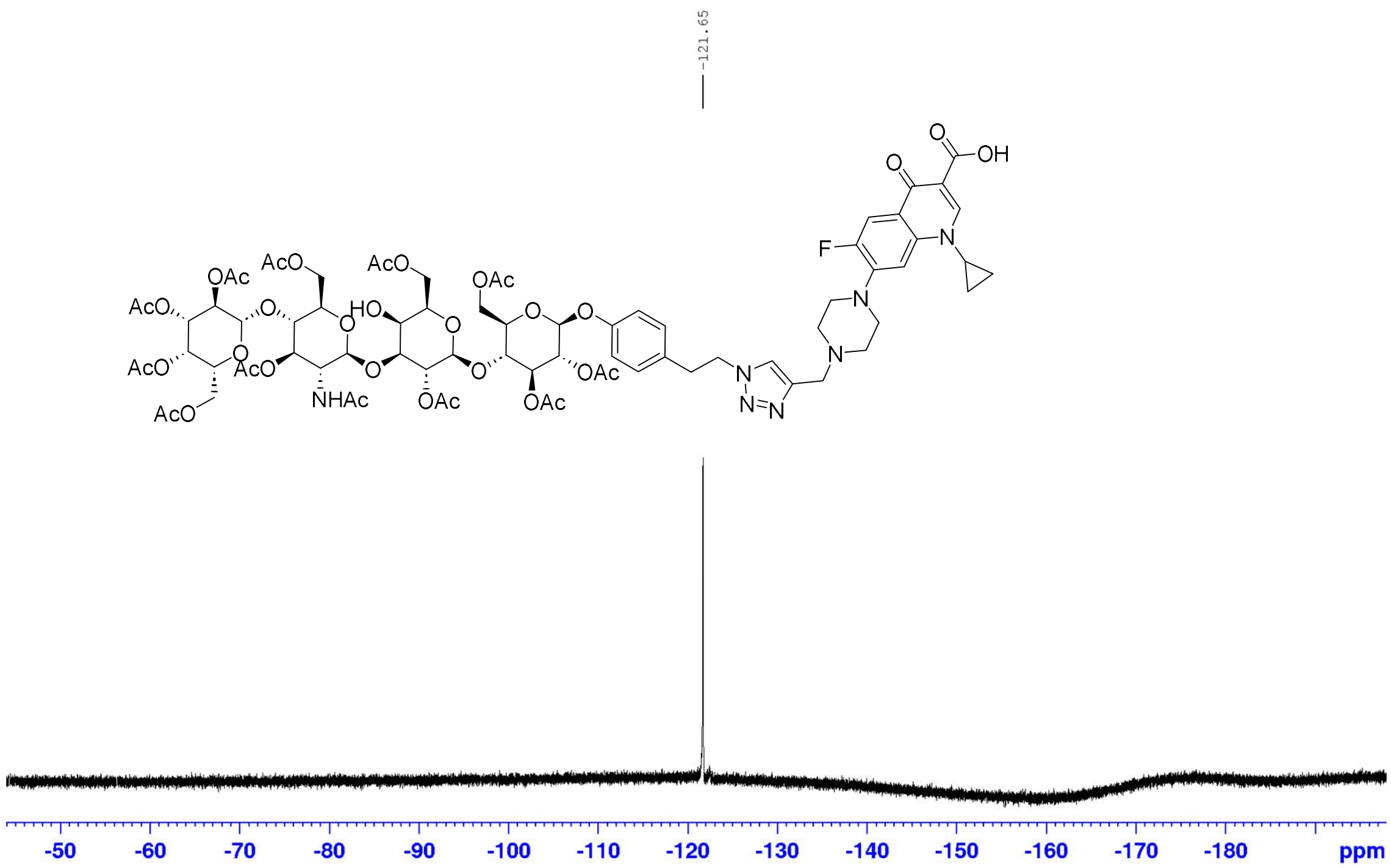

^{19}F NMR (376 MHz, CD_3OD) spectrum of compound **12c.**

^{1}H NMR (400 MHz, CD_3OD) spectrum of compound **12d.**

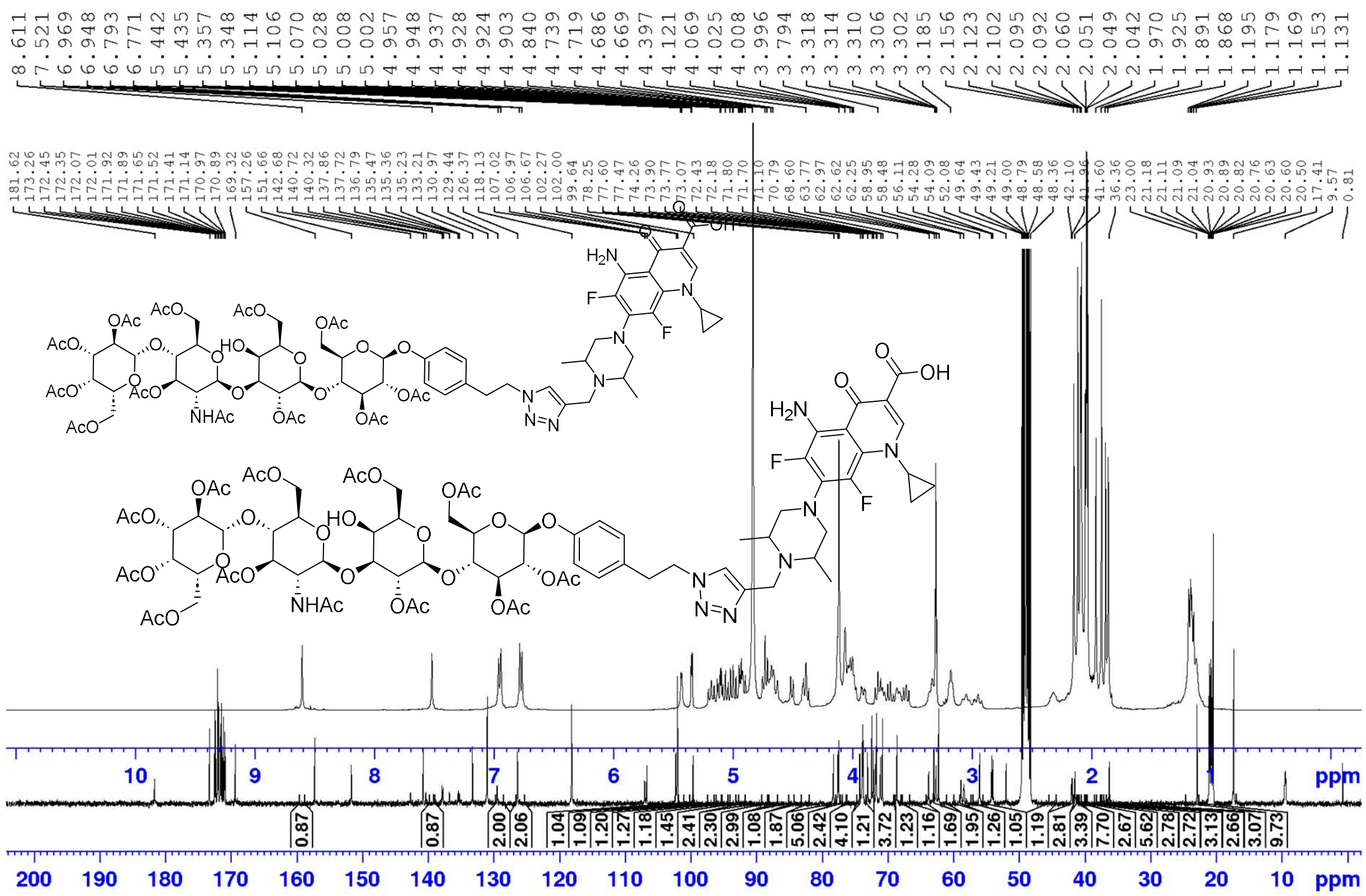

^{13}C NMR (100 MHz, CD_3OD) spectrum of compound **12d.**

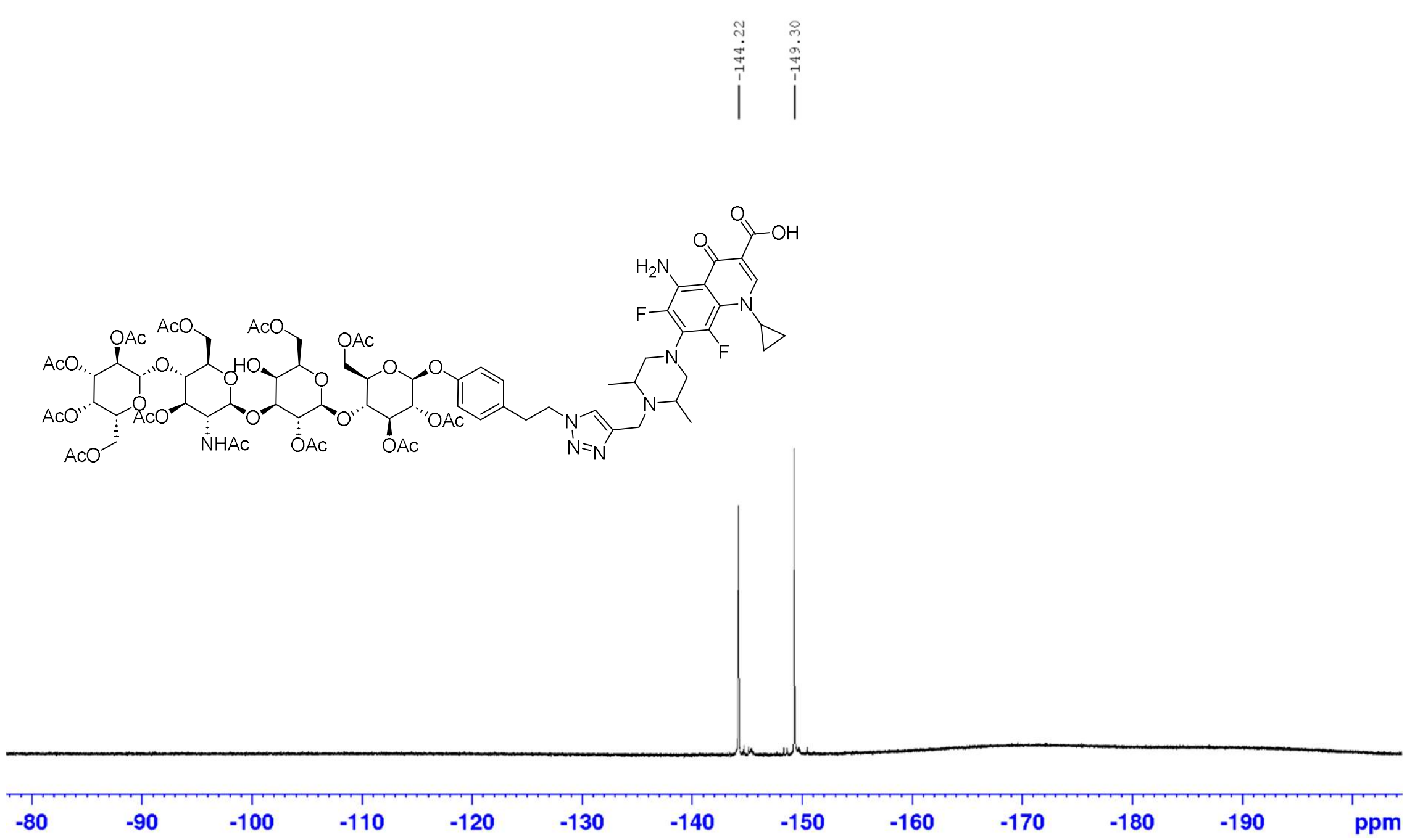

^{19}F NMR (376 MHz, CD_3OD) spectrum of compound **12d.**

References

(1) a) https://www.protocols.io/view/anti-bacterial-growth-inhibition-assay-81wgbjjk3vpk/v1 (accessed August 4, 2026; b) K. S. Håheim, M. A., K. Helland, J. H. Andersen, M. O. Sydnes. *Hererocycles* **2023**, *106* (9), 1559-1569.
(2) Cheng, H. C., X.; Xian, M.; Fang, S.; Cai, T. B.; Ji, J. J.; Tunac, J. B.; Sun, D.; Wang, P. G. *J. Med. Chem.* **2005**, *48*, 645-652.
(3) McPherson III, J. C. R., R.; Buston, T. B.; Hartmann, J. F.; Farcasiu, D.; Bereczki, I.; Roth, E.; Tollas, S.; Ostorhazi, E.; Rozgonyi, F.; Herczegh, P. *Eur. J. Med. Chem* **2012**, *47*, 615-618.
(4) Agarwal, A. S., P.; Maurya, A.; Patel, U. K.; Singh, A.; Nath, G. *ACS Omega* **2022**, *7*, 2725–2736.
(5) Yang, Y. S., J. M.; Hang, H. C. *J. Am. Chem. Soc.* **2010**, *132*, 3640-3641.
